# Molecular and morphological characterization of nine new *Gnomoniopsis* species (*Diaporthales*, *Gnomoniaceae*): Genomic insights into evolution and phylogeny

**DOI:** 10.3897/imafungus.17.208364

**Published:** 2026-10-23

**Authors:** Xingsheng Wang, Yang Jiang, Wenwen Liu, Xigang Yan, Jinyan Sheng, Congcong Ai, Xiaoyong Liu, Xiuguo Zhang, Zhe Meng, Shi Wang

**Affiliations:** 1 College of Life Sciences, Shandong Normal University, Jinan, 250358, China College of Life Sciences, Shandong Normal University Jinan China; 2 Shandong Provincial Key Laboratory for Biology of Vegetable Diseases and Insect Pests, College of Plant Protection, Shandong Agricultural University, Taian, 271018, China Shandong Provincial Key Laboratory for Biology of Vegetable Diseases and Insect Pests, College of Plant Protection, Shandong Agricultural University Taian China

**Keywords:** Fungal taxonomy, genome analysis, morphological traits, new taxa, species delimitation

## Abstract

*Gnomoniopsis* comprises parasitic, endophytic, and saprophytic fungi. Since its establishment, this genus has presented considerable taxonomic challenges owing to extensive morphological overlap among species, rendering traditional classification systems that rely predominantly on phenotypic characteristics no longer applicable. To resolve the current taxonomic confusion and clarify the species diversity, phylogenetic relationships, and genomic adaptive characteristics of *Gnomoniopsis*, the present study was carried out. In this study, the internal transcriptional spacer (ITS), translation extension factor 1-α (*tef1*), and partial beta-tubulin (*tub2*) were analyzed and combined to observe morphological characteristics of *Gnomoniopsis*. The isolates were identified from chestnut (*Castanea*), *Embelia vestita*, tea (*Camellia sinensis*), and other hosts in Sichuan Province, as well as from *Quercus* species and partial leaf rot from Fujian Province. Nine new species, namely *G. xiaoheijiangensis***sp. nov**., *G. embeliae***sp. nov**., *G. yunvfengensis***sp. nov**., *G. quercus***sp. nov**., *G. ledongensis***sp. nov**., *G. yonganensis***sp. nov**., *G. conghuaensis***sp. nov**., *G. loropetali***sp. nov**., and *G. qixiensis***sp. nov**., were obtained. Phylogenomic analysis based on 7,942 orthologous proteins’ clusters revealed distinct species boundaries within *Gnomoniopsis*. Phylogenomics has thus effectively resolved the ambiguous phylogenetic relationships in this genus. This work identifies nine previously undescribed *Gnomoniopsis* species, clarifies the species diversity and conserved genomic characteristics within the genus, and lays a foundation for subsequent taxonomic analyses of *Gnomoniopsis*.

## Introduction

The taxonomic study of *Gnomoniopsis* originated from early morphological investigations (Sogonov et al. 2008). Initially, Berlese treated it as a subgenus of *Gnomonia* based on septate ascospore features. Later, owing to the poor taxonomic stability of these morphological traits, this group was temporarily merged into *Gnomonia*. The year 2010 represented a crucial turning point for the systematics of this genus. Based on molecular phylogenetic analyses of ITS, *tub2* and *tef1*, Walker et al. (2010) confirmed its monophyly, established it as a distinct genus, proposed new species and new combinations, and compiled an identification key for 13 species, which laid a foundation for its systematic study (ME 1978; Walker et al. 2010). Subsequently, relevant studies expanded globally, and it was confirmed that *Gnomoniopsis* mainly colonizes host plants of *Fagaceae*, *Rosaceae* and other plant families. Since 2018, research on *Gnomoniopsis* in China has accelerated (Walker et al. 2010; Jiang and Tian 2019; Yang et al. 2020; Jiang et al. 2021b). Several studies integrated morphological and molecular phylogenetic evidence to discover multiple new species across several Chinese provinces, greatly enriching the domestic species record and advancing the taxonomic and distribution research of this genus in East Asia.

*Gnomoniopsis* comprises parasitic, endophytic, and saprophytic fungi within *Gnomoniaceae*, *Diaporthales*, *Sordariomycetes*, *Ascomycota*. *Gnomoniopsis* is widely distributed throughout the world and mainly colonizes plant tissues such as *Fagaceae*, *Salicaceae* and *Rosaceae*. (Voglmayr et al. 2012, 2017). To date, 54 species of *Gnomoniopsis* have been recorded in Index Fungorum (http://www.indexfungorum.org/, accessed on 25 March 2026), among which the most typical pathogens were *G. clavulata* (Sogonov et al. 2008), *G. smithogilvyi* (Crous et al. 2012), *G. daii* (Jiang and Tian 2019) and *G. chinensis* (Jiang et al. 2020). These pathogens can cause plant disasters such as white oak (*Quercus alba*) and chestnut (*C. mollissima*) in North America, causing great losses to the local economy (Yang et al. 2020; Jiang et al. 2021a).

The mycelium of *Gnomoniopsis* typically grows within plant tissues, is septate, hyaline or pale in color (Zhang et al. 2025b). The sexual stage produces the ascomata, which is generally buried in the host tissue or semi-buried, has an ostiole, is usually spherical or subspherical, and varies in color from light to dark. The ascus is clavate or cylindrical and contains multiple ascus spores, which are generally single-celled, colorless, or pale, oval to fusiform, and vary in size and morphology from species to species (Walker et al. 2010; Wang et al. 2022). The asexual stage can produce conidia. Conidia are morphologically variable, ranging from cylindrical to ellipsoid. Conidia are borne on conidiophores, which are solitary or fasciculate (Jiang et al. 2022).

Breakthroughs in modern sequencing approaches now enable genomics to effectively trace fungal evolutionary lineages and uncover how pathogens trigger infection processes (Farrer et al. 2017; Wang et al. 2023b; Ruan et al. 2024; Mondo and Grigoriev 2025; Naqvi et al. 2025; Zhang et al. 2025a). Functional genomic features, including carbohydrate-active enzymes (CAZymes) and secondary metabolite biosynthetic gene clusters (BGCs), are key genetic components that determine fungal host adaptation, cell wall degradation capacity, and pathogenic potential (Cantarel et al. 2009; Keller 2019). Nevertheless, genomic data focusing on the fungal genus *Gnomoniopsis* remain extremely scarce. As of December 7, 2025, only four described species in this genus have publicly available whole- genome sequences in the NCBI database. The lack of sufficient genomic resources severely limits our understanding of interspecific evolutionary divergence and functional differentiation within *Gnomoniopsis*. Given the unresolved taxonomic ambiguity, insufficient species investigation, and limited genomic information for *Gnomoniopsis*, the present study was conducted to explore the species diversity, phylogenetic relationships, and functional genomic characteristics of endemic *Gnomoniopsis* fungi in China through integrated morphological, multi-locus phylogenetic, and comparative genomic analyses.

## Materials and methods

### Sample processing and morphological analysis

To broaden sampling coverage and obtain diverse plant materials, diseased and decaying leaf samples were collected in 2025 from forested areas of Yunnan, Hainan, Fujian, Northeast China, and other provinces across China. This study included fungal strains isolated from two types of plant materials: fresh symptomatic leaf tissues with active necrotic lesions and naturally decaying senescent leaves. Isolates obtained from symptomatic materials were collected from fresh, pathogen-damaged leaf tissues with typical necrotic spots and lesion browning, representing parasitic or pathogenic fungal populations. Other strains were isolated from naturally senescent and decaying leaves collected in the field, which represent saprophytic or endophytic fungal resources colonizing dead plant tissues. Completely decomposed fragments with ambiguous symptoms were excluded during sampling to ensure accurate isolation sources. From these collections, nine new fungal species described in the present study were isolated and identified from lesions on *Embelia vestita*, *Lindera communis* Hemsl., *Quercus griffithii* and *Castanopsis chinensis*. Lesion samples (5 mm × 5 mm) were cut from diseased leaves with scissors. The samples were soaked in 75% ethanol for 1 minute, then rinsed with sterile water for 30 seconds, followed by immersion in 5% hypochlorous acid solution for 1 minute, and finally rinsed with sterile water three times. After cleaning, the samples were placed on sterilized clean filter paper to absorb moisture, and then transferred to the surface of potato dextrose agar (PDA) medium (composed of 200 g potato, 20 g agar powder, 20 g glucose, 1000 mL distilled water, with pH adjusted to 7.0). After marking the date and sample number, the medium was incubated in a dark environment at 25°C for 3–5 days. The growth of hyphae around the leaf samples was observed regularly; once a certain number of hyphae appeared around the small leaf fragments the outgrowing marginal hyphae with no visible contamination were selected, a 5 mm × 5 mm agar block with hyphae was cut using an inoculating needle and transferred to a new PDA plate for further cultivation to obtain pure cultures. Single-hypha purification was repeated 2–3 times until axenic cultures without contaminant fungi or bacteria were confirmed. On the 7^th^, 14^th^, and 21^st^ days, images of the front and back sides of the colonies were captured using a digital camera (Canon PowerShot G7X; Canon Inc., Beijing, China). A stereomicroscope (Olympus SZX10; Olympus Optical Co., Ltd., Beijing, China) and an optical microscope (Olympus BX53; Olympus Optical Co., Ltd., Japan) were used to observe the growth status of hyphae and sporulation. A high-definition digital camera (Olympus DP80; Olympus Optical Co., Ltd., Tokyo, Japan) was employed to photograph fungal structures such as conidiophores, conidial structures, and conidia. The microscopic structures of each strain were measured using Digimizer, with 20–30 samples measured for each structure. All strains were preserved in a 10% sterile glycerol solution and stored at 4°C. The type specimens were deposited in the Herbarium of Shandong Agricultural University (SAUCC). Voucher specimens were deposited in the Herbarium of the Department of Plant Pathology, Shandong Agricultural University (HSAUP, Taian, China), and the Herbarium Mycologicum Academiae Sinicae (HMAS, Beijing, China) of the Institute of Microbiology, Chinese Academy of Sciences. The living type cultures were deposited in the Culture Collection Center of Shandong Agricultural University (SAUCC).

### DNA extraction and sequencing

Genomic DNA of the strains grown on PDA plates was extracted using the CTAB (cetyltrimethylammonium bromide) method. When the hyphae grew adequately on PDA plates, 0.2 g of hyphae was scraped off with a sterile scalpel under aseptic conditions and transferred to a 1.5 mL centrifuge tube. Pre-warmed CTAB buffer was added to the tube, followed by homogenization of the hyphae using the steel bead grinding method. After complete homogenization, the centrifuge tube containing the lysate was placed in a 65°C water bath for 2 hours to facilitate nucleic acid release and increase cell membrane permeability. A centrifuge was used to separate the aqueous phase (4°C, 12,000 rpm, 10 minutes). The clear supernatant was transferred to a new sterile centrifuge tube, and an equal volume of chloroform-isoamyl alcohol mixture (24:1) was added to induce phase separation, allowing genomic DNA to be selectively partitioned into the aqueous layer (Wang et al. 2023a; Zhang et al. 2025b). After thorough vortexing, a second centrifugation was performed. Subsequently, the upper aqueous phase was collected and subjected to another round of chloroform-isoamyl alcohol extraction to improve DNA purity, preparing for the subsequent precipitation step.

In this study, the extracted genomic DNA was amplified via polymerase chain re-action (PCR) using the ITS, *tef1*, and *tub2* genes (see Table 1). The primers used were as follows: ITS: ITS4/ITS5; *tef1*: EF1-728F/EF2; *tub2*: Bt-2a/Bt-2b. The PCR amplification system had a total volume of 25 µL, which contained 9.5 µL of double-distilled water (ddH_2_O), 12.5 µL of 2×Taq Plus Master Mix (with dye, purchased from Yeasen Bio-technology Co., Ltd., Shanghai, China; product No.: 10154ES03), 1 µL of forward and reverse primers, and 1 µL of the strain DNA extract. The PCR amplification products were detected by 2% agarose gel electrophoresis, and the amplification effect was observed under an ultraviolet lamp. Subsequent primer synthesis and gene sequencing were entrusted to Sangon Biotech (Shanghai) Co., Ltd. After receiving the sequencing results, MEGA v.7.0 software was used for sequence analysis, comparison, and assembly. The processed sequences were compared using BLAST (http://blast.ncbi.nlm.nih.gov/). The sequences of the five taxa introduced in this study, obtained after comparison, have been uploaded to and deposited in GenBank of the National Center for Biotechnology Information (NCBI, USA). The GenBank accessions referenced in this study were collated and summarized in the Table 2.

**Table 1.** Molecular markers and their PCR primers and programs used in this study.

**Loci**	**PCR Primers**	**Sequence (5’—3’)**	**PCR Cycles**	**References**
ITS	ITS4	TCC TCC GCT TAT TGA TAT GC	(95°C: 30 s, 55°C: 30 s, 72°C: 1 min) ×35 cycles	White et al. (1990)
	ITS5	GGA AGT AAA AGT CGT AAC AAG G		
*tef1*	EF1-728F	CAT CGA GAA GTT CGA GAA GG	(95°C: 30 s, 48°C: 30 s, 72°C: 1 min) ×35 cycles	Carbone and Kohn (1999)
	EF2	GGA RGT ACC AGT SAT CAT GTT		O’Donnell et al. (1998)
*tub2*	Bt-2a	GGT AAC CAA ATC GGT GCT GCT TTC	(95°C: 30 s, 53°C: 30 s, 72°C: 1 min) ×35 cycles	Glass and Donaldson (1995)
	Bt-2b	ACC CTC AGT GTA GTG ACC CTT GGC		

**Table 2.** GenBank numbers used in the phylogenetic analysis of *Gnomoniopsis*.

**Taxon**	**Strain**	**GenBank Accession Numbers**			
		**ITS**	***tef1***	***tub2***	**References**
*Apiognomonia errabunda*	AR 2813	DQ313525	DQ313565	DQ862014	Sogonov et al. (2007)
*Gnomoniopsis alderdunensis*	CBS 125679	GU320826	GU320813	GU320788	Walker et al. (2010)
*G. alderdunensis*	CBS 125680*	GU320825	GU320801	GU320787	Walker et al. (2010)
*G. alderdunensis*	CBS 125681	GU320827	GU320802	GU320789	Walker et al. (2010)
*G. angolensis*	CBS 145057*	MK047428	NA	NA	Crous et al. (2018)
*G. annonae*	CGMCC3.28195*	PQ325623	PQ336654	PQ336678	Zhang et al. (2025b)
*G. annonae*	SAUCC6333	PQ325624	PQ336655	PQ336679	Zhang et al. (2025b)
*G. annonae*	CGMCC3.28194	PQ325625	PQ336656	PQ336680	Zhang et al. (2025b)
*G. annonae*	SAUCC6423	PQ325626	PQ336657	PQ336681	Zhang et al. (2025b)
*G. castanopsidis*	CFCC 54437*	MZ902909	MZ936385	NA	Jiang et al. (2021b)
*G. castanopsidis*	CFCC 55878	MZ902910	MZ936386	NA	Jiang et al. (2021b)
*G. chamaemori*	CBS 804.79	GU320817	GU320809	GU320777	Walker et al. (2010)
*G. chinensis*	CFCC 52286*	MG866032	MH545370	MH545366	Jiang et al. (2020)
*G. chinensis*	CFCC 52287	MG866033	MH545371	MH545367	Jiang et al. (2020)
*G. chinensis*	CFCC 52288	MG866034	MH545372	MH545368	Jiang et al. (2020)
*G. chinensis*	CFCC 52289	MG866035	MH545373	MH545369	Jiang et al. (2020)
*G. clavulata*	CBS 121255	EU254818	GU320807	EU219211	Sogonov et al. (2008)
*G. comari*	CBS 806.79	EU254821	GU320810	EU219156	Sogonov et al. (2008)
*G. comari*	CBS 807.79	EU254822	GU320814	GU320779	Sogonov et al. (2008)
*G. comari*	CBS 809.79	EU254823	GU320794	GU320778	Sogonov et al. (2008)
***G. conghuaensis***	SAUCC1614603*	PZ169404	PZ191632	PZ189102	**This study**
***G. conghuaensis***	SAUCC1614604	PZ169405	PZ191633	PZ189103	**This study**
*G. daii*	CFCC 54043*	MN598671	MN605519	MN605517	Jiang and Tian (2019)
*G. daii*	CMF002B	MN598672	MN605520	MN605518	Jiang and Tian (2019)
*G. daii*	CFCC 55517	MZ902911	MZ936387	MZ936403	Jiang and Tian (2019)
*G. daii*	CFCC 55294B	MZ902912	MZ936388	MZ936404	Jiang and Tian (2019)
*G. diaoluoshanensis*	SAUCC DL0963*	ON753744	ON759769	ON759777	Wang et al. (2022)
*G. diaoluoshanensis*	SAUCC DL0964	ON753743	ON759768	ON759776	Wang et al. (2022)
***G. embeliae***	SAUCC925701*	PV134370	PZ207028	PX409985	**This study**
***G. embeliae***	SAUCC925702	PV134371	PZ207029	PX409986	**This study**
*G. euryae*	CGMCC3.28234*	PQ325629	PQ336660	PQ336684	Zhang et al. (2025b)
*G. euryae*	SAUCC3977	PQ325630	PQ336661	PQ336685	Zhang et al. (2025b)
*G. fagacearum*	CFCC 54316*	MZ902916	MZ936392	MZ936408	Jiang et al. (2021b)
*G. flava*	CFCC 71563*	PV257808	PV268106	PV339811	Li et al. (2025)
*G. flava*	CFCC 71566	PV257809	PV268107	PV339812	Li et al. (2025)
*G. flava*	CFCC 71567	PV257810	PV268108	PV339813	Li et al. (2025)
*G. fragariae*	CBS 121226	EU254824	GU320792	EU219144	Udayanga et al. (2021)
*G. fragariae*	CBS 208.34	EU254826	GU320808	EU219149	Udayanga et al. (2021)
*G. fragariae*	CBS 125671	GU320816	GU320793	GU320776	Udayanga et al. (2021)
*G. fujianensis*	CGMCC3.28229*	PQ325641	PQ336672	PQ336696	Zhang et al. (2025b)
*G. fujianensis*	SAUCC1260	PQ325642	PQ336673	PQ336697	Zhang et al. (2025b)
*G. guangdongensis*	CFCC 54443*	MZ902918	MZ936394	MZ936410	Jiang et al. (2021b)
*G. guangdongensis*	CFCC 54331	MZ902919	MZ936395	MZ936411	Jiang et al. (2021b)
*G. guangdongensis*	CFCC 54282	MZ902920	MZ936396	MZ936412	Jiang et al. (2021b)
*G. guttulata*	MS 0312	EU254812	NA	NA	Walker et al. (2010)
*G. hainanensis*	CFCC 54376*	MZ902921	MZ936397	MZ936413	Jiang et al. (2021b)
*G. hainanensis*	CFCC 55877	MZ902922	MZ936398	MZ936414	Jiang et al. (2021b)
*G. idaeicola*	CBS 125672	GU320823	GU320797	GU320781	Walker et al. (2010)
*G. idaeicola*	CBS 125673	GU320824	GU320798	GU320782	Walker et al. (2010)
*G. idaeicola*	CBS 125674	GU320820	GU320796	GU320780	Walker et al. (2010)
*G. idaeicola*	CBS 125675	GU320822	GU320799	GU320783	Walker et al. (2010)
*G. idaeicola*	CBS 125676	GU320821	GU320811	GU320784	Walker et al. (2010)
*G. juglandis*	CGMCC3.28233*	PQ325637	PQ336668	PQ336692	Zhang et al. (2025b)
*G. juglandis*	SAUCC2375	PQ325638	PQ336669	PQ336693	Zhang et al. (2025b)
*G. lanceolata*	CGMCC3.28232*	PQ325639	PQ336670	PQ336694	Zhang et al. (2025b)
*G. lanceolata*	SAUCC1277	PQ325640	PQ336671	PQ336695	Zhang et al. (2025b)
***G. ledongensis***	SAUCC1528003*	PX353467	PZ207036	PZ195445	**This study**
***G. ledongensis***	SAUCC1528004	PX353468	PZ207037	PZ195446	**This study**
*G. lithocarpi*	SAUCC YN0743*	ON753749	ON759765	ON759783	Wang et al. (2022)
*G. lithocarpi*	SAUCC YN0742	ON753750	ON759764	ON759782	Wang et al. (2022)
***G. loropetali***	SAUCC1994101*	PZ169406	PZ191634	PZ189104	**This study**
***G. loropetali***	SAUCC1994102	PZ169407	PZ191635	PZ189105	**This study**
*G. luodingensis*	CGMCC 3.29462*	PX611876	PX655142	NA	Guo et al. (2026)
*G. luodingensis*	GXCM RG119	PX611877	PX655141	NA	Guo et al. (2026)
*G. macounii*	CBS 121468	EU254762	GU320804	EU219126	Sogonov et al. (2008)
*G. melastomatis*	CGMCC3.28231*	PQ325631	PQ336662	PQ336686	Zhang et al. (2025b)
*G. melastomatis*	SAUCC0031	PQ325632	PQ336663	PQ336687	Zhang et al. (2025b)
*G. melastomatis*	SAUCC3040	PQ325635	PQ336666	PQ336690	Zhang et al. (2025b)
*G. melastomatis*	SAUCC0051	PQ325636	PQ336667	PQ336691	Zhang et al. (2025b)
*G. mengyinensis*	SAUCC MY0293*	ON753741	ON759766	ON759774	Wang et al. (2022)
*G. mengyinensis*	SAUCC MY0296	ON753742	ON759767	ON759775	Wang et al. (2022)
*G. occulta*	CBS 125677	GU320828	GU320812	GU320785	Walker et al. (2010)
*G. occulta*	CBS 125678	GU320829	GU320800	GU320786	Walker et al. (2010)
*G. paraclavulata*	CBS 123202	GU320830	GU320815	GU320775	Sogonov et al. (2008)
***G. qixiensis***	SAUCC1995302*	PZ169408	PZ191636	PZ189106	**This study**
***G. qixiensis***	SAUCC1995303	PZ169409	PZ191637	PZ189107	**This study**
***G. quercus***	SAUCC1204202a*	PV134366	PZ207034	PZ195443	**This study**
***G. quercus***	SAUCC1204202b	PV134367	PZ207035	PZ195444	**This study**
*G. racemula*	CBS 121469*	EU254841	GU320803	EU219125	Sogonov et al. (2008)
*G. rosae*	CBS 145 085*	MK047451	NA	NA	Crous et al. (2018)
*G. rosae*	CFCC 57557	ON564617	NA	NA	Crous et al. (2018)
*G. rossmaniae*	CFCC 54307*	MZ902923	MZ936399	MZ936415	Jiang et al. (2021b)
*G. rossmaniae*	CFCC 55876	MZ902924	MZ936400	MZ936416	Jiang et al. (2021b)
*G. sanguisorbae*	CBS 858.79	GU320818	GU320805	GU320790	Walker et al. (2010)
*G. saprophytica*	CGMCC3.28192*	PQ325633	PQ336664	PQ336688	Zhang et al. (2025b)
*G. saprophytica*	SAUCC3342	PQ325634	PQ336665	PQ336689	Zhang et al. (2025b)
*G. silvicola*	CFCC 54304	MZ902925	MZ936401	MZ936417	Jiang et al. (2021b)
*G. silvicola*	CFCC 54418*	MZ902926	MZ936402	MZ936418	Jiang et al. (2021b)
*G. smithogilvyi*	CBS 130190*	JQ910642	KR072534	JQ910639	Crous et al. (2012)
*G. smithogilvyi*	CBS 130189	JQ910644	KR072535	JQ910641	Crous et al. (2012)
*G. smithogilvyi*	CBS 130188	JQ910643	KR072536	JQ910640	Crous et al. (2012)
*G. smithogilvyi*	MUT 401	HM142946	KR072537	KR072532	Crous et al. (2012)
*G. smithogilvyi*	MUT 411	HM142948	KR072538	KR072533	Crous et al. (2012)
*G. taishanensis*	CGMCC3.28230*	PQ325645	PQ336676	PQ336700	Zhang et al. (2025b)
*G. taishanensis*	SAUCC1033	PQ325646	PQ336677	PQ336701	Zhang et al. (2025b)
*G. tormentillae*	CBS 904.79	EU254856	GU320795	EU219165	Sogonov et al. (2008)
***G. xiaoheijiangensis***	SAUCC921604*	PV134372	PZ191630	PZ189100	**This study**
***G. xiaoheijiangensis***	SAUCC921605	PV134373	PZ191631	PZ189101	**This study**
*G. xishuangbannaensis*	CGMCC3.25980*	PQ325643	PQ336674	PQ336698	Zhang et al. (2025b)
*G. xishuangbannaensis*	CGMCC3.25979	PQ325644	PQ336675	PQ336699	Zhang et al. (2025b)
*G. xunwuensis*	CFCC 53115*	MK432667	MK578141	MK578067	Yang et al. (2020)
*G. xunwuensis*	CFCC 53116	MK432668	MK578142	MK578068	Yang et al. (2020)
***G. yonganensis***	SAUCC1167501*	PV167928	PZ207032	PZ195441	**This study**
***G. yonganensis***	SAUCC1167502	PV167929	PZ207033	PZ195442	**This study**
*G. yunnanensis*	SAUCC YN1659*	ON753746	ON759771	ON759779	Wang et al. (2022)
*G. yunnanensis*	SAUCC YN1657	ON753747	ON759772	ON759780	Wang et al. (2022)
***G. yunvfengensis***	SAUCC1132501*	PV134368	PZ207030	PX412667	**This study**
***G. yunvfengensis***	SAUCC1132502	PV134369	PZ207031	PX412668	**This study**

Notes: New species established in this study are shown in bold. The ex-type strain is indicated using “*”. N/A: Not available.

### Whole-genome sequencing and genome assembly and annotation

The strain was inoculated into PDB medium (35 g of PDB powder was weighed and dissolved in 1000 mL of distilled water, then aliquoted and autoclaved at 121 °C for 30 min; Beijing Solarbio Science & Technology Co., Ltd., Cat# P9240), and cultured in shake flasks at 25 °C and 100 rpm for 7 days. Mycelia were collected under aseptic conditions, frozen in liquid nitrogen for 5 min, and stored at −80 °C. The samples were sent to Wuhan Benagen Technology Co., Ltd. for library construction and high-throughput sequencing.

Raw data were subjected to quality control using fastp v1.0 (Chen 2025). The filtered reads were subjected to de novo assembly using SPAdes v3.15.5 with k-mers set to 21, 33, and 55 (Prjibelski et al. 2020). All data have been deposited in the National Center for Biotechnology Information (NCBI) database under the BioProject accession number PRJNA1456304; the corresponding GenBank accession numbers for the genome sequences are listed in Table 3.

**Table 3.** BioSample and SRA NCBI number of the taxa used in phylogenomic analyses in this study.

**Species**	**Strains**	**BioSample**	SRA NCBI*	**References**
*Discula destructiva*	AS111	SAMEA118781011	ERS25271958	From NCBI
*Gnomoniopsis castaneae*	MUT401	SAMN31615005	NA	Turco et al. (2023)
*G. castaneae*	ICMP14040	SAMN31615006	NA	Turco et al. (2023)
*G. castaneae*	GN01	SAMN31615007	NA	Turco et al. (2023)
***G. conghuaensis***	**SAUCC1614603**	**SAMN57401259**	SRR38217291	**This study**
***G. embeliae***	**SAUCC925701**	**SAMN57401254**	SRR38217296	**This study**
***G. ledongensis***	**SAUCC1528003**	**SAMN57401257**	SRR38217292	**This study**
***G. loropetali***	**SAUCC1994101**	**SAMN57401260**	SRR38217290	**This study**
***G. qixiensis***	**SAUCC1995302**	**SAMN57401261**	SRR38217289	**This study**
***G. quercus***	**SAUCC1204202a**	**SAMN57401256**	SRR38217293	**This study**
*G. smithogilvyi*	IMI 355082	SAMN30184414	SRS14569010	From NCBI
*Gnomoniopsis* sp.	YN1632	SAMN40373142	SRS20766660	From NCBI
*Gnomoniopsis* sp.	YN0743	SAMN40373141	SRS20766659	From NCBI
*Gnomoniopsis* sp.	IMI 355080	SAMN30184413	SRS14569009	From NCBI
***G. yonganensis***	**SAUCC1167501**	**SAMN57401258**	SRR38217294	**This study**
***G. yunvfengensis***	**SAUCC1132501**	**SAMN57401255**	SRR38217295	**This study**
***G. xiaoheijiangensis***	**SAUCC921604**	**SAMN57401253**	SRR38217297	**This study**

* Species information described in this study is marked in bold. N/A: Not available.

Repetitive sequence families of the genome were annotated de novo using RepeatModeler v.2.0.5.(Flynn et al. 2020) The predicted results were combined with the RepBase and Dfam databases. Subsequently, genome-wide repetitive sequence annotation was performed using RepeatMasker v.4.1.6 (Chen 2004) with the parameters: -e rmblast -gff -xsmall -html -norna).

Ribosomal RNA (rRNA) genes were predicted by combining RNAmmer v.1.2 and HMMER v.2.0(Lagesen et al. 2007). Transfer RNA (tRNA) genes in the genome were identified using tRNAscan-SE v.2.0.(Chan et al. 2021). The Infernal software bundled with Rfam v.15.1 was employed to predict miRNA and snRNA sequences (Kalvari et al. 2018).

Protein-coding gene prediction was performed in three steps: first, de novo gene prediction was conducted using Augustus v3.3.3 (Stanke et al. 2004); second, protein-coding genes in the target genome were predicted based on protein-coding genes of homologous species using GeMoMa v1.9 (Keilwagen et al. 2016; Keilwagen et al. 2018); finally, EVidenceModeler v2.0.0 (Haas et al. 2008; Haas et al. 2011) was used to integrate the results from de novo prediction and homological based prediction.

### Phylogenetic and phylogenomic analysis

Nucleic acid data of *Gnomoniopsis* were obtained from the National Center for Bi-otechnology Information (NCBI) database (https://www.ncbi.nlm.nih.gov/, accessed 20 May 2025), and related GenBank accession codes were taken from the latest version of a relevant research paper. Multiple sequence alignment for nucleotide sequences of the new *Gnomoniopsis* taxa and publicly accessible reference data was done using MAFFT 7 (http://mafft.cbrc.jp/alignment/server/, accessed 21 May 2025). The concatenated multi-locus alignment was manually inspected and trimmed in MEGA to remove poorly aligned and ambiguous flanking regions; intron regions of *tef1* and *tub2* were fully retained, and only ambiguous terminal regions were deleted. Phylogenetic studies of the aligned data were carried out via maximum likelihood (ML) and Bayesian inference (BI) methods. Both analytical ways were run on their own after signing up on the CIPRES website. ML-based phylogenetic inference was performed with RAxML-HPC2 v.8.2.12 on XSEDE resources, adopting the GTRGAMMA nucleotide substitution model and using 1000 rapid bootstrap replicates to assess confidence. MrModeltest v.2.3 software was used to screen for the optimal evolutionary model, while BI analysis was conducted with MrBayes 3.2.7a on the XSEDE platform. The inferred phylogenetic tree was visualized and re-rooted with an outgroup using FigTree v1.4.3 (http://tree.bio.ed.ac.uk/software/figtree/, accessed 24 May 2025). The final tree was prepared with Adobe Illustrator CC 2019, where names of the new species were highlighted in red for easy visual distinction.

### Prediction of secondary metabolite biosynthetic gene clusters and carbohydrate-active enzymes

The FASTA-formatted scaffold assembly and GFF-formatted Predicted_Genes file derived from second-generation whole-genome sequencing were submitted to the antiSMASH fungal web server (https://fungismash.secondarymetabolites.org); analyses were performed under default parameters, and biosynthetic gene clusters encoding secondary metabolites of *Gnomoniopsis* were retrieved from the generated result link, while carbohydrate-active enzymes (CAZymes) were annotated using dbCAN3 implemented via run_dbcan by combining Diamond homology search and HMM profile analysis, with predicted protein sequences as input and CAZyme domains identified following the default criteria of the dbCAN pipeline.

### Split network inference and pairwise homoplasy index (PHI) test

Sequence alignments concatenated from ITS, *tef1*, and *tub2* loci covering nine undescribed *Gnomoniopsis* species and closely related reference strains were imported into SplitsTree v.4.16.1 (www.splitstree.org) for recombination screening (Huson 1998; Huson and Bryant 2006). The LogDet distance transformation coupled with the NeighborNet algorithm was adopted to generate split networks for visualizing genetic differentiation among all operational taxonomic units. Meanwhile, pairwise homoplasy index (PHI) statistical tests were performed on the unified alignment matrix to identify signals of interspecific homologous recombination. All newly isolated strains obtained in this study were marked in red text within the network graph, which was exported as Suppl. material 3 to provide supporting evidence for species boundary delimitation (Quaedvlieg et al. 2014).

### Pathogenicity tests

To assess the pathogenic potential of the nine undescribed *Gnomoniopsis* taxa, artificial inoculation trials were carried out on detached plant foliage. Given the practical obstacles to consistently obtaining sufficient intact, disease-free fresh tissues from each fungus’s respective native host, healthy mature leaves harvested from cultivated Chinese chestnut (*Castanea mollissima*) were selected as a universal substrate for all inoculation treatments.

All fungal strains were revitalized on potato dextrose agar (PDA) medium and cultured under constant temperature conditions (25 °C) for seven days to generate uniform mycelial mats. Intact chestnut leaves were surface disinfected using sequential ethanol and sodium hypochlorite rinses, followed by triple washes with sterile distilled water and air-drying under aseptic conditions. Sterile fine needles were used to create minor uniform wounds on the leaf epidermis. Circular mycelial plugs with a diameter of 5 mm were cut from the actively growing edge of each pure fungal colony and placed tightly onto the wounded leaf regions. For negative control groups, blank PDA plugs devoid of fungal mycelium were placed on identically scratched leaf tissues without fungal inoculum.

All treated leaf samples were placed into sealed plastic moist chambers and incubated at 25 °C with a 12 h light / 12 h dark alternating photoperiod. The progression of leaf discoloration and necrotic lesions was observed and recorded daily. To fulfill the criteria of Koch’s postulates, leaf segments bearing obvious necrotic lesions were excised at 14 days post inoculation. Tissue fragments collected at the boundary between healthy leaf tissue and lesion areas were subjected to surface sterilization and tissue isolation. Purified isolates recovered from diseased materials were identified via morphological observation and multi-locus sequencing of ITS, *tef1* and *tub2* loci to confirm their identity with the original inoculated strains (Li et al. 2025).

## Results

### Phylogenetic analyses

**Figure 1.**
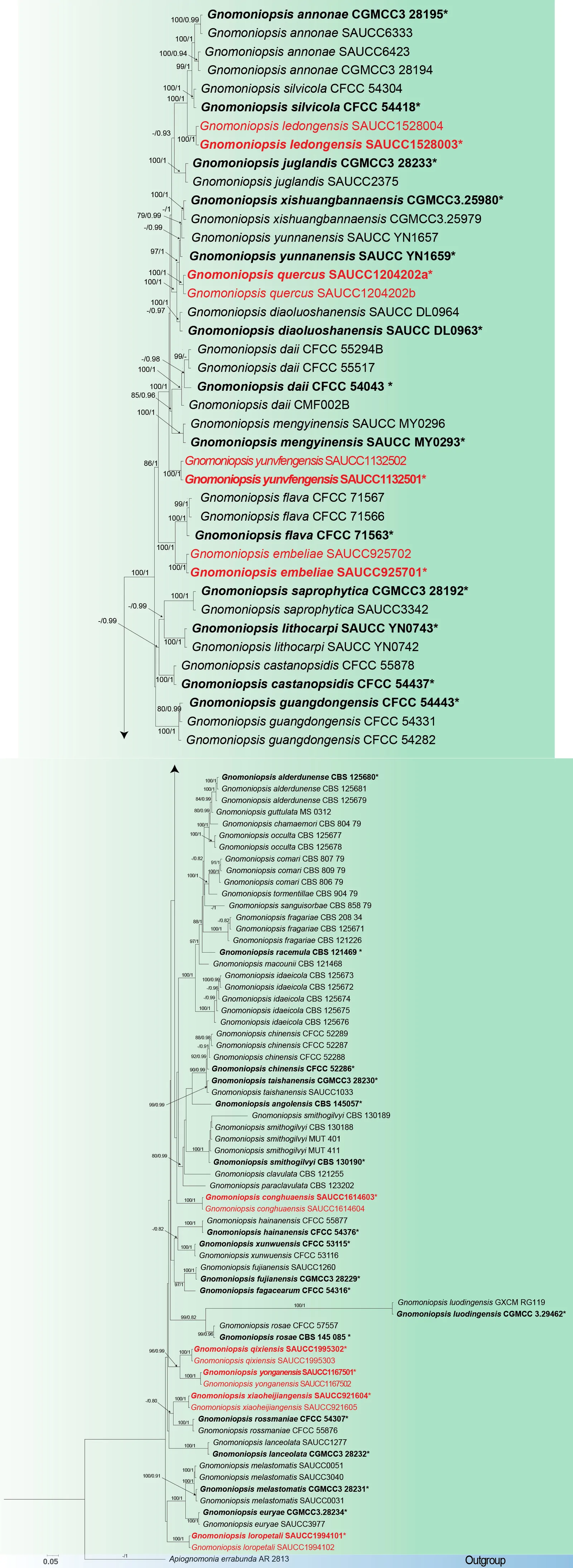
Maximum likelihood inference tree based on a combined dataset of analyzed ITS, *tef1*, *tub2* sequences. The Bayesian inference posterior probability (right, BIPP ≥ 0.80) and the maximum likelihood bootstrap value (left, MLBV ≥ 70%) are shown as MLBV/BIPP above the nodes. Those marked “*” in the tree are represented as ex-type or epitype strains. Strains isolated in this study are indicated in red. The scale bar at the bottom indicates 0.05 substitutions per site.

Phylogenetic analysis clarified the evolutionary relationships among species groups within the genus *Gnomoniopsis*. Multi-gene concatenated sequences were retrieved from GenBank and assembled, with *Apiognomonia errabunda* AR 2813 designated as the outgroup to construct the phylogenetic sequence matrix. The matrix comprised 50 isolates representing *Gnomoniopsis* and its allied taxa, containing a total of 2,450 sequence sites: the ITS region (sites 1–665), the *tef1* region (sites 666–1620), and the *tub2* region (sites 1621–2450). Among these sites, 1,191 were conserved, 241 were parsimony-uninformative, and 1,018 were parsimony-informative. MrModelTest analyses indicated that the Dirichlet base frequencies and the GTR+I+G evolutionary model should be adopted for Bayesian inference across all three partitions. Figure 1 presents the maximum likelihood (ML) phylogenetic tree with the highest score, and nodal supports are annotated with maximum likelihood bootstrap values and Bayesian posterior probabilities (marked as MLBS / BPP). A total of nine novel *Gnomoniopsis* isolates were incorporated into the phylogenomic analyses, and the results were consistent with the maximum likelihood (ML) tree mentioned above (Fig. 1). Based on the phylogenetic analyses, nine new taxa of the genus *Gnomoniopsis* were introduced in the present study for the first time, including *G. xiaoheijiangensis* sp. nov., *G. embeliae* sp. nov., *G. yunvfengensis* sp. nov., *G. quercus* sp. nov., *G. ledongensis* sp. nov., *G. yonganensis* sp. nov., *G. conghua­ensis* sp. nov., *G. loropetali* sp. nov., *G. qixiensis* sp. nov.

To clarify the phylogenetic independence of novel species in the genus *Gnomoniopsis*, species delimitation analyses were performed following the Genealogical Concordance Phylogenetic Species Recognition (GCPSR) criterion, and the Pairwise Homoplasy Index (PHI) test was conducted to evaluate genetic recombination among closely related taxa. A Φw value of 0.05 was set as the critical threshold: a Φw value below 0.05 indicates significant genetic recombination between taxa, whereas values exceeding this threshold reflect effective genetic differentiation and distinct species boundaries. The PHI tests performed on the novel *Gnomoniopsis* species all yielded Φw values greater than 0.05, with no significant signals of genetic recombination detected. Collectively, the PHI recombination analyses verify that these novel taxa are markedly differentiated from their close relatives at both phylogenetic and evolutionary levels, and thus can be recognized as valid, independent species. All PHI test plots for the novel *Gnomoniopsis* species are provided in Suppl. material 3.

### Phylogenomic analysis in *Gnomoniopsis*

**Figure 2.**
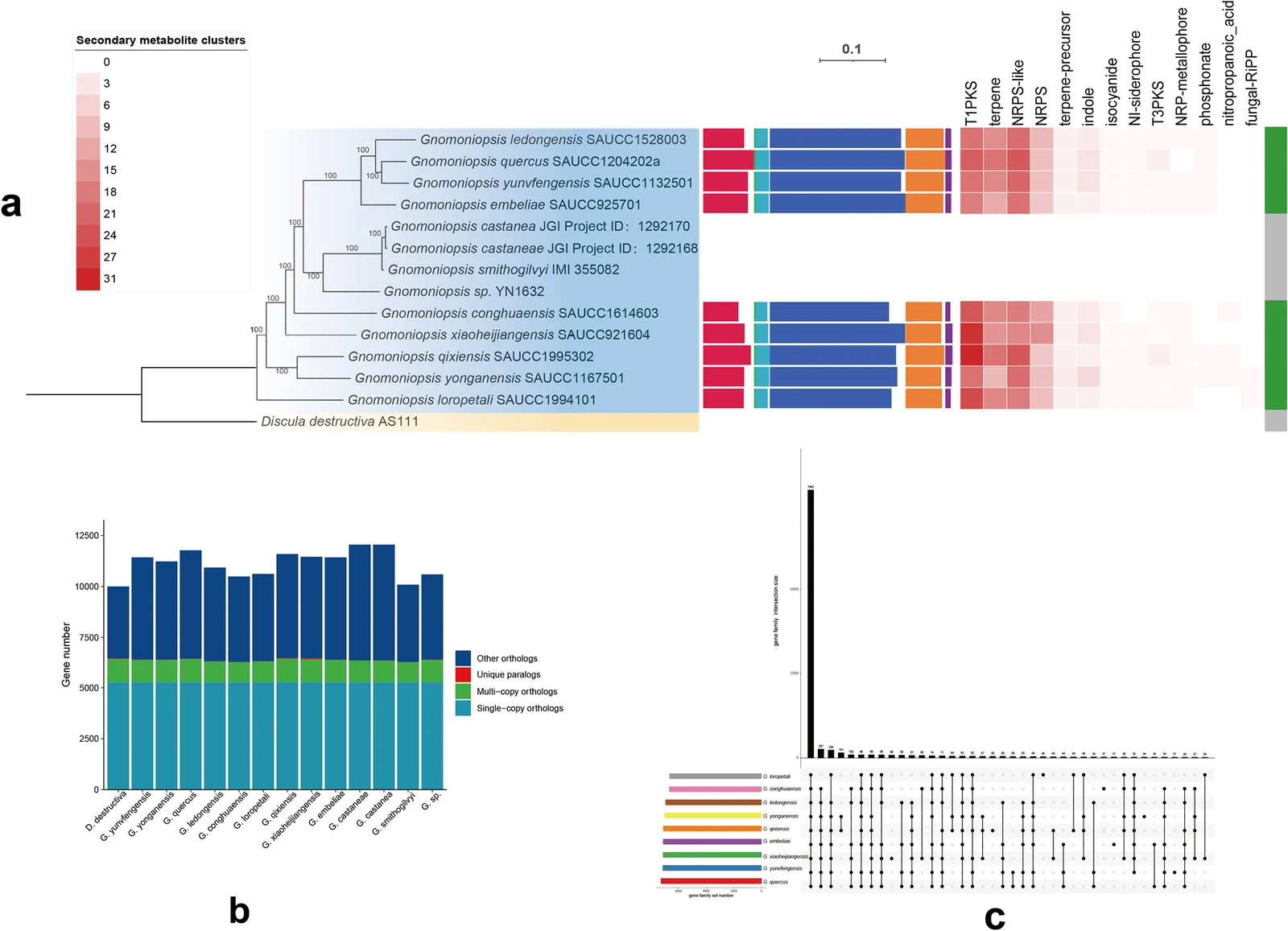
*Gnomoniopsis* genome analysis. **a**. Phylogenetic relationships, composition of carbohydrate-active enzymes (CAZymes), and distribution of secondary metabolite biosynthetic gene clusters among nine novel *Gnomoniopsis* strains. **b**. Bar chart of homologous genes in each strain. **c**. UpSet plot of nine strains showing intersection counts among different strains in the form of bar charts.

In this study, we sequenced the genomes of nine strains of *Gnomoniopsis*, together with four published genomes of closely related species obtained from the NCBI (https://www.ncbi.nlm.nih.gov/datasets/) and JGI (https://genome.jgi.doe.gov/portal/) databases. Using *Discula destructiva* (AS111) as the outgroup, a maximum likelihood (ML) phylogenetic tree was constructed based on 7,942 clusters of orthologous protein-coding genes (Fig. 2a).

The RAxML phylogenetic tree constructed from CDS sequences clearly resolved the phylogenetic relationships among strains of the genus *Gnomo­niopsis*. All taxa formed well-supported monophyletic clades with high bootstrap support values. The outgroup was located at the base of the tree, supporting the robustness of the phylogenetic topology. Branch lengths reflected the level of genetic divergence, indicating distinct interspecific differentiation and strong intraspecific conservation within *Gnomoniopsis*.

In the gene family number bar chart of the genus *Gnomoniopsis* (Fig. 2b), the composition of homologous genes among strains shows both commonalities and differences. Single-copy orthologous genes account for a relatively large proportion, indicating that these species in the genus *Gnomoniopsis* possess numerous conserved single-gene functions in the basic genetic framework. The presence of strain-specific unique genes may endow each strain with specific ecological adaptation or functional differentiation potential, such as specificity in host interaction and environmental stress.

Consistent with this evolutionary pattern, CAZyme profiles exhibited moderate interspecific variation among *Gnomoniopsis* strains, with total CAZyme counts ranging from 505 to 596. Glycoside hydrolases (GHs) constituted the predominant family, whereas carbohydrate-binding modules (CBMs) were completely conserved with four copies in all isolates. In total, 727 secondary-metabolite biosynthetic gene clusters (BGCs) were predicted across the nine strains, which varied from 67 to 94 per genome and were predominantly represented by T1PKS, NRPS-like, terpene and NRPS clusters. No strong correlation was detected between CAZyme gene count and BGC abundance, implying that carbohydrate-degrading capability and secondary-metabolite diversity are shaped by different selective forces. Several strain-specific BGC classes were also detected, which may further contribute to lineage-specific host-interaction and stress-adaptation traits (Fig. 2a).

### Taxonomy

#### Gnomoniopsis conghuaensis

Taxon classificationAnimaliaDiaporthalesGnomoniaceae

X.S. Wang, Y. Jiang, S. Wang & X.G. Zhang sp. nov.

71DC3042-847E-5B46-9E24-D0845F2B3E5C

863465

Fig. 3

##### Holotype.

CHINA • Guangdong Province, Guangzhou City, Conghua District, 23°38'26"N, 113°45'56"E, from decaying leaves, 1 March 2025, X.S. Wang, holotype HMAS 354534, ex-type living culture SAUCC1614603; paratype, ex-paratype living culture SAUCC1614604.

**Figure 3.**
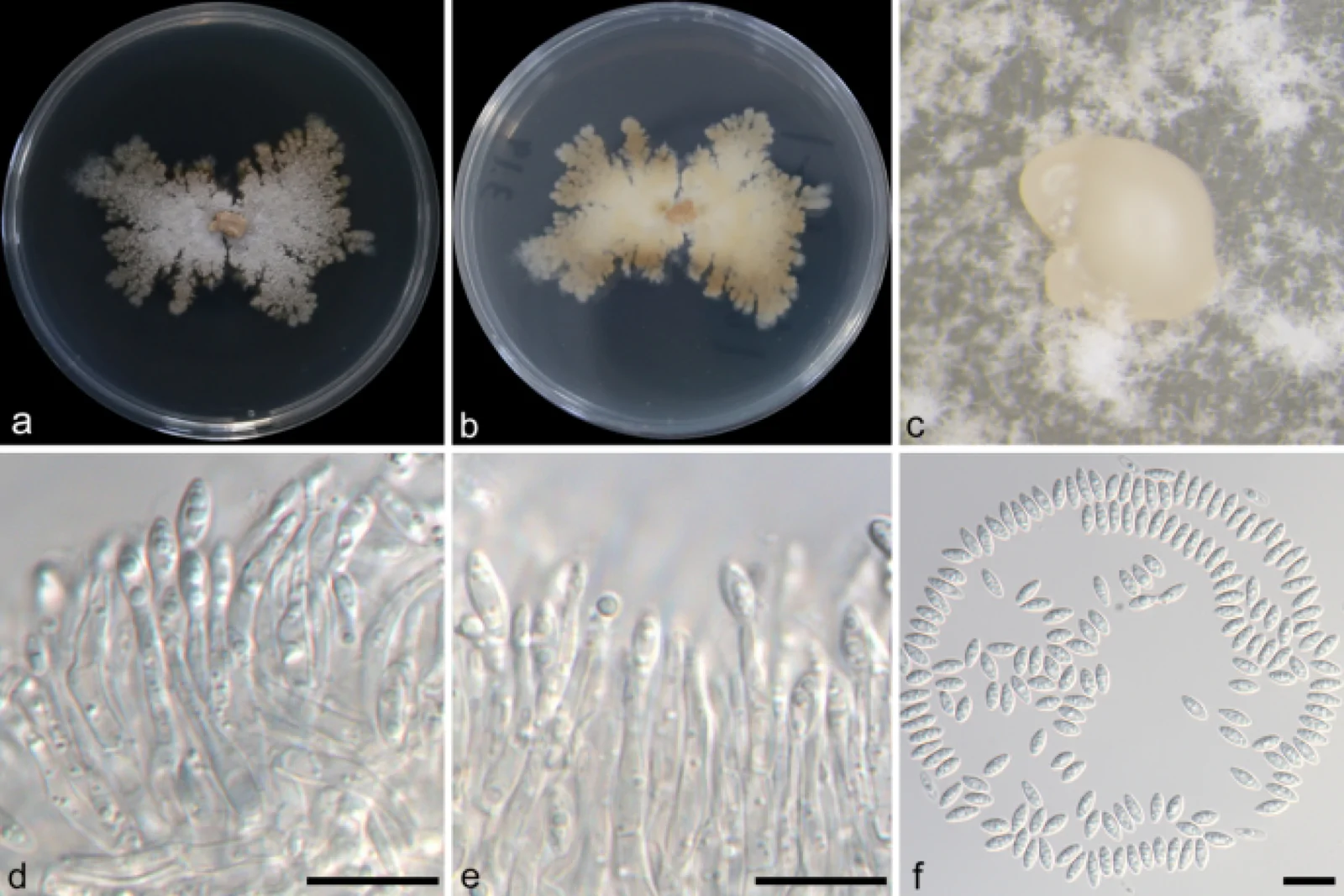
*Gnomoniopsis conghuaensis* (holotype: HMAS 354534). **a**. Surface of colony after 14 days on PDA; **b**. Reverse side of colony after 14 days on PDA; **c**. Colony overview; **d, e**. Conidiophores with attached conidia; **f**. Conidia. Scale bars: 10 µm (**d–f**).

##### Etymology.

The specific epithet is named after Conghua District, the locality of its host plant collection.

##### Description.

The sporodochia are convex, globose or irregularly massive, mostly solitary and rarely gregarious, white in color. After incubation at 25 °C in darkness for 14 days, abundant whitish, droplet-like, slimy conidial masses exude from the surface. Conidiophores are mostly cylindrical, tapering from the base to the apex, hyaline and smooth-walled, densely arranged with immature conidia at the tip; dimensions 10.4–22.0 × 1.2–2.6 µm (mean = 16.2 × 2.0 µm, L/W = 4.0–18.3 µm, n = 20). Conidia are fusiform, hyaline, smooth, slightly curved in part, measuring 5.6–8.1 × 2.0–3.4 µm (mean = 6.8 × 2.6 µm, L/W = 1.6–4.1 µm, n = 25). Sexual morph was not observed.

##### Culture characteristics.

After incubation for 14 days in darkness at 25 °C on PDA medium, the colony diameter reaches 20.2–55.3 mm, with a growth rate of 1.4–3.9 mm per day. Colonies are flat and irregular in overall shape. The inner mycelium grows luxuriantly while the peripheral mycelium is sparse; the colony is overall off-white with pale brown margins. Off-white sporodochia are scattered irregularly on the colony surface.

##### Notes.

Phylogenetic analysis based on the combined sequences of ITS, *tef1* and *tub2* genes revealed that *Gnomoniopsis conghuaensis* is closely related to *G. hainanensis* (CFCC 55877) and *G. paraclavulata* (CBS 123202). Meanwhile, nucleotide sequence differences exist among them: the nucleotide divergences between *G. conghuaensis* and *G. hainanensis* are ITS: 45/515 (8.74%), *tef1*: 75/240 (31.25%), *tub2*: 63/364 (17.31%), and those between *G. conghuaensis* and *G. paraclavulata* are ITS: 39/518 (7.53%), *tef1*: 67/352 (19.03%), *tub2*: 54/344 (15.70%). Morphologically, the conidiophores of both species are hyaline and smooth-walled; however, those of *G. hainanensis* are longer than those of *G. conghuaensis* (16.5–26.0 × 2.5–4.5 µm, L/W = 3.7–10.4 µm vs. 10.4–22.0 × 1.2–2.6 µm, L/W = 4.0–18.3 µm). The conidia of *G. hainanensis* are ovate, whereas those of *G. conghuaensis* are fusiform. In addition, the conidia of *G. hainanensis* are larger than those of *G. conghuaensis* (8–10 × 3.4–3.9 µm, L/W = 2.1–2.9 µm vs. 5.6–8.1 × 2.0–3.4 µm, L/W = 1.6–4.1 µm).

#### Gnomoniopsis embeliae

Taxon classificationAnimaliaDiaporthalesGnomoniaceae

X.S. Wang, Y. Jiang, S. Wang & X.G. Zhang sp. nov.

D5CBC320-501E-5740-8C35-39CC44F20F96

860858

Fig. 4

##### Holotype.

CHINA • Yunnan Province, Pu’er City, Ning’er Hani and Yi Autonomous County, Xiaoheijiang Forest Park, 23°12'21"N, 100°55'31"E, from diseased leaves of *Castanea mollissima*, 16 May 2024, X.S. Wang, holotype HMAS 354062, ex-type living culture SAUCC925701; paratype, ex-paratype living culture SAUCC925702.

**Figure 4.**
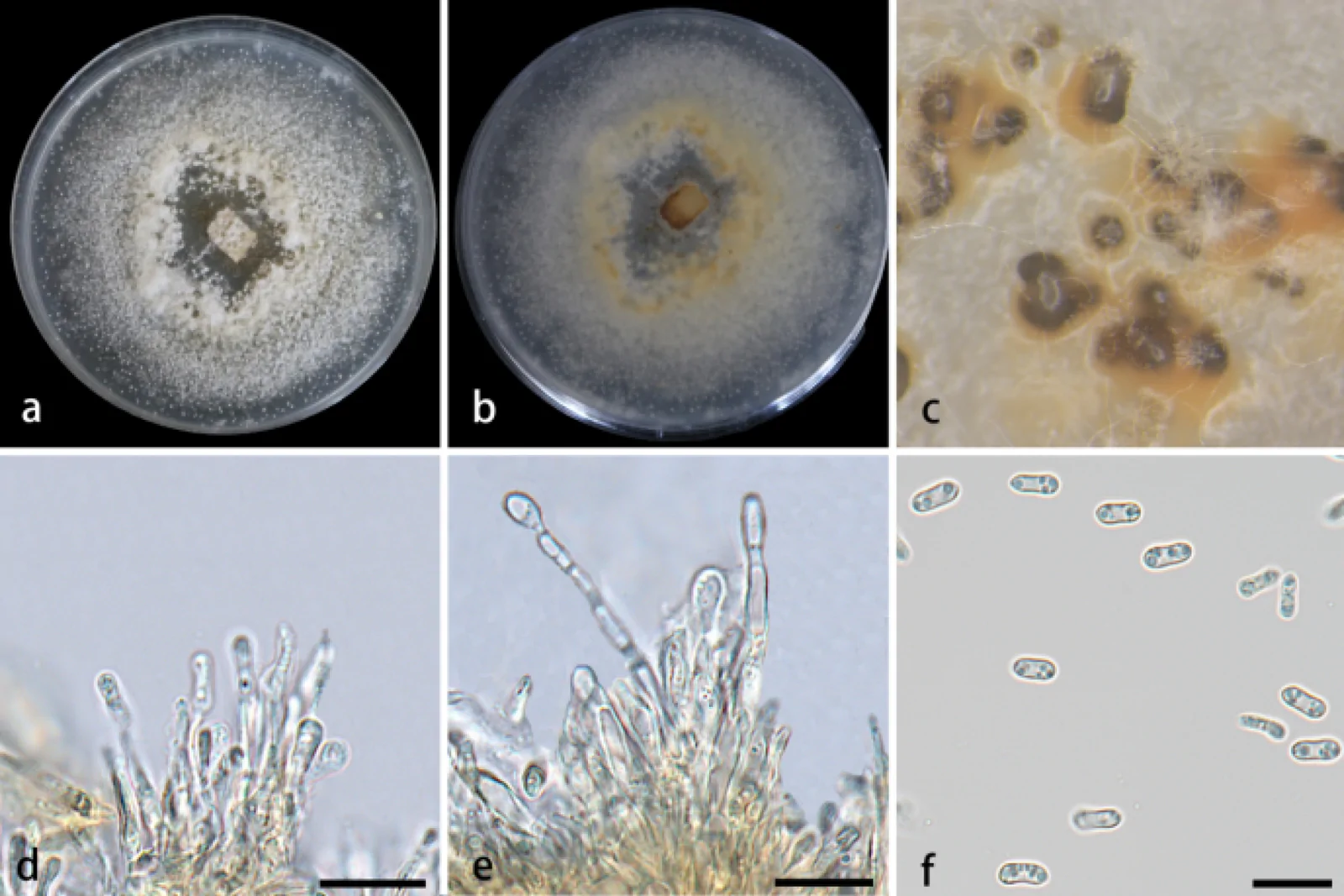
*Gnomoniopsis embeliae* (holotype: HMAS 354062). **a**. Surface of colony after 14 days on PDA; **b**. Reverse side of colony after 14 days on PDA; **c**. Colony overview; **d, e**. Conidiophores with attached conidia; **f**. Conidia. Scale bars: 10 µm (**d–f**).

##### Etymology.

The specific epithet “*embeliae*” refers to the host genus *Embelia*.

##### Description.

Sporodochium protrudes, forming spherical or blocky shapes, aggregates or solitary, is light brown in color, and its surface exudes pale yellow mucus-like conidia aggregates. Conidiophores are mostly tubular, gradually narrowing from the base towards the top. The cells are colorless and transparent, with a smooth surface, closely and orderly arranged, and gradually thinning towards the top 12.4–33.2 × 1.8–2.3 µm (mean = 22.8 × 2.0 µm, L/W = 5.4–18.4 µm, n = 20). Conidia are oval or spindle-shaped, with some individuals slightly curved, transparent and smooth 4.2–7.4 × 1.8–2.7 µm (mean = 6.1 × 2.2 µm, L/W = 1.6–4.1 µm, n = 25). Sexual morph was not observed.

##### Culture characteristics.

After 14 days of cultivation at 25°C in the dark, the colony on PDA reaches a diameter of 80.6–82.7 mm, with a growth rate of 5.7–5.9 mm per day. The colony is relatively flat overall: the hyphae inside the colony are sparse, while those in the outer region grow uniformly, giving the colony an overall grayish-white appearance. Light brown conidiomata are distributed in the central area of the colony.

##### Notes.

Phylogenetic analysis based on the combined gene sequences of ITS, *tef1*, and *tub2* shows that *Gnomoniopsis embeliae* forms a distinct clade (100%/1) and is closely related to *G. flava* (CFCC 71563). The differences in their nucleic acid sequences are 13/552 (2.36%) for ITS, 47/550 (8.55%) for *tef1*, and 26/465 (5.59%) for *tub2*. Morphologically, the conidia of *G. flava* are longer than those of *G. embeliae* (6.5–8 × 2.5–3 µm, L/W = 2.2–3.2 µm in *G. flava* vs. 4.2–7.4 × 1.8–2.7 µm, L/W = 1.6–4.1 µm in *G. embeliae*).

#### Gnomoniopsis ledongensis

Taxon classificationAnimaliaDiaporthalesGnomoniaceae

X.S. Wang, Y. Jiang, S. Wang & X.G. Zhang sp. nov.

2D5172C9-5B57-517B-9590-4D2DE3C37EB4

860862

Fig. 5

##### Holotype.

CHINA • Hainan Province, Ledong Li Autonomous County, Township Road 001, 18°43'31"N, 108°50'6"E, from diseased leaves of *Quercus griffithii*, 4 December 2024, X.S. Wang, holotype HMAS 354066, ex-type living culture SAUCC1528003; paratype, ex-paratype living culture SAUCC1528004.

**Figure 5.**
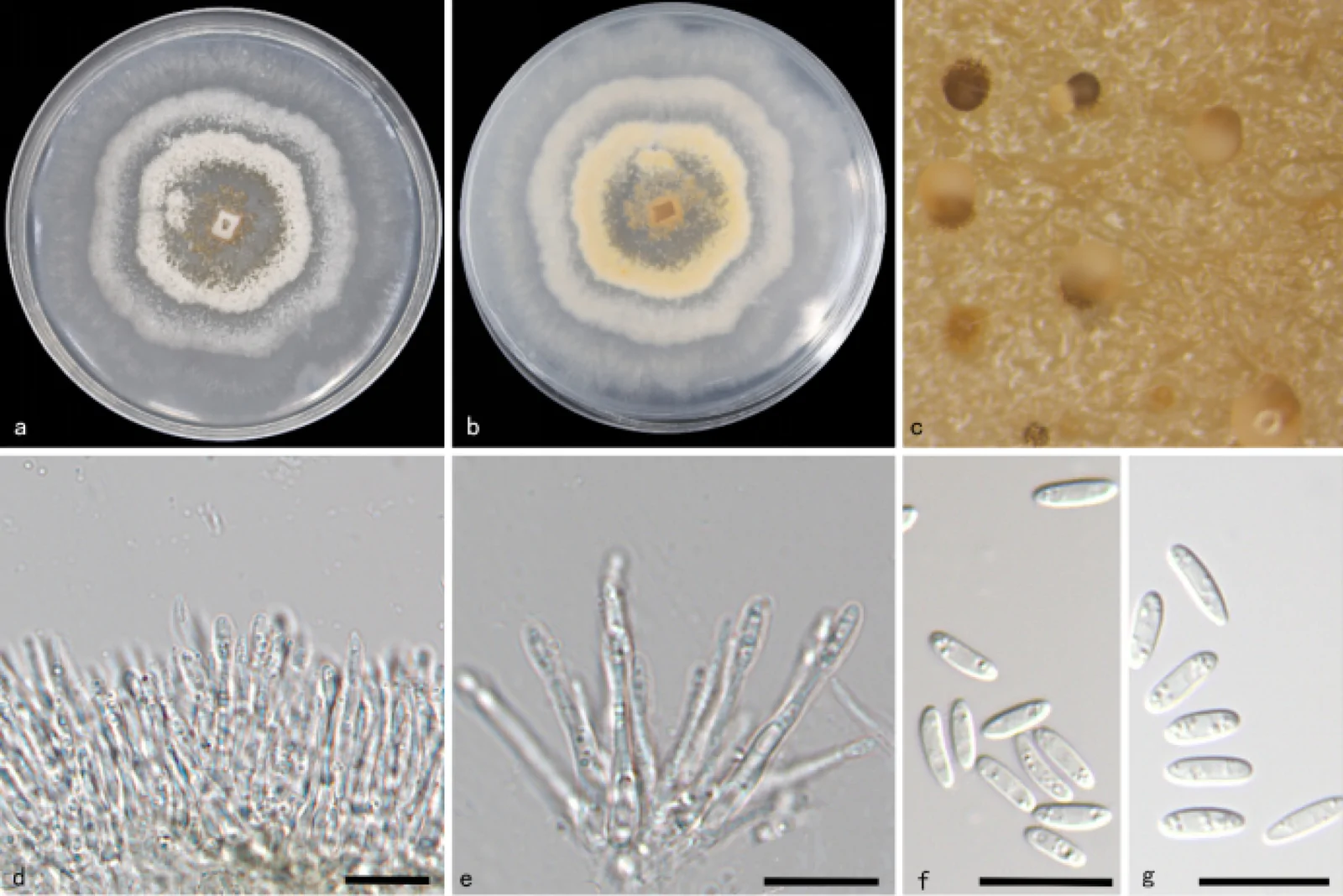
*Gnomoniopsis ledongensis* (holotype: HMAS 354066). **a**. Surface of colony after 14 days on PDA; **b**. Reverse side of colony after 14 days on PDA; **c**. Colony overview; **d, e**. Conidiophores with attached conidia; **f, g**. Conidia. Scale bars: 10 µm (**d–g**).

##### Etymology.

The specific epithet “*ledongensis*” is derived from Ledong Li Autonomous County, where the specimen was collected.

##### Description.

The sporodochia are protruding, appearing as clumps or small spheres, either solitary or clustered, and are black or light brown. When cultured in a 25°C incubator under dark conditions for 4 to 7 days, a pale yellow, viscous, cream-like aggregate of conidia exudes from the surface of the conidiomata. Conidiophores are columnar, contain septa, and taper gradually from the base to the apex, with conidia growing at the top. They are colorless and transparent, and densely arranged on the surface of the conidiomata 20.9–32.5 × 1.3–2.4 µm (mean = 26.7 × 1.8 µm, L/W = 8.7–25.0 µm, n = 20). A single conidium is fusiform or spindle-shaped, transparent and colorless 4.6–7.4 × 1.5–2.4 µm (mean = 6.1 × 1.9 µm, L/W = 1.9–4.9 µm, n = 25). Sexual morph was not observed.

##### Culture characteristics.

After 14 days of cultivation on PDA at 25°C in the dark, the colony diameter reaches 80.6–82.1 mm, with a growth rate of 5.7–5.9 mm per day. The colony is flat overall; most hyphae exhibit large and regular wavy growth, giving the colony an overall grayish-white appearance. Conidiomata are distributed linearly from the center to the periphery, while a small portion of hyphae grow in clumps.

##### Notes.

Phylogenetic analyses revealed that the newly proposed species *Gnomoniopsis ledongensis* in this study forms an independent clade together with *G. silvicola* (CFCC 54418) and *G. annonae* (CGMCC3.28195), indicating their close phylogenetic relationship (100%/1). However, nucleotide sequence differences exist between *G. ledongensis* and *G. silvicola*, ITS: 8/505 (1.58%); *tef1*: 24/298 (8.05%); *tub2*: 10/464 (2.16%), as well as between *G. ledongensis* and *G. annonae*, ITS: 9/587 (1.53%); *tef1*: 48/620 (7.74%); *tub2*: 14/491 (2.85%). Morphologically, there are also multiple differences among the three taxa. The conidiophores differ as follows: (20.9–32.5 × 1.3–2.4 µm, L/W = 8.7–25.0 µm vs. 7–15 × 1.5–2.5 µm, L/W = 2.8–10.0 µm vs. 7–11.5 × 1.3–2 µm, L/W = 3.5–8.8 µm). Conidia differ as: (4.6–7.4 × 1.5–2.4 µm, L/W = 1.9–4.9 µm vs. 4.5–5.3 × 2.2–2.6 µm, L/W = 1.7–2.4 µm vs. 4.6–6.8 × 1.6–2.2 µm, L/W = 2.1–4.3 µm).

#### Gnomoniopsis loropetali

Taxon classificationAnimaliaDiaporthalesGnomoniaceae

X.S. Wang, Y. Jiang, S. Wang & X.G. Zhang sp. nov.

47DF036C-9302-5E05-81BA-258AEA9FD3C6

863466

Fig. 6

##### Holotype.

CHINA • Zhejiang Province, Quzhou City, Kaihua County, Qixi Town, 29°23'25.22"N, 118°12'35.95"E, from diseased leaves of *Loropetalum chinense*, 19 June 2025, X.S. Wang, holotype HMAS 354535, ex-type living culture SAUCC1994101; paratype, ex-paratype living culture SAUCC1994102.

**Figure 6.**
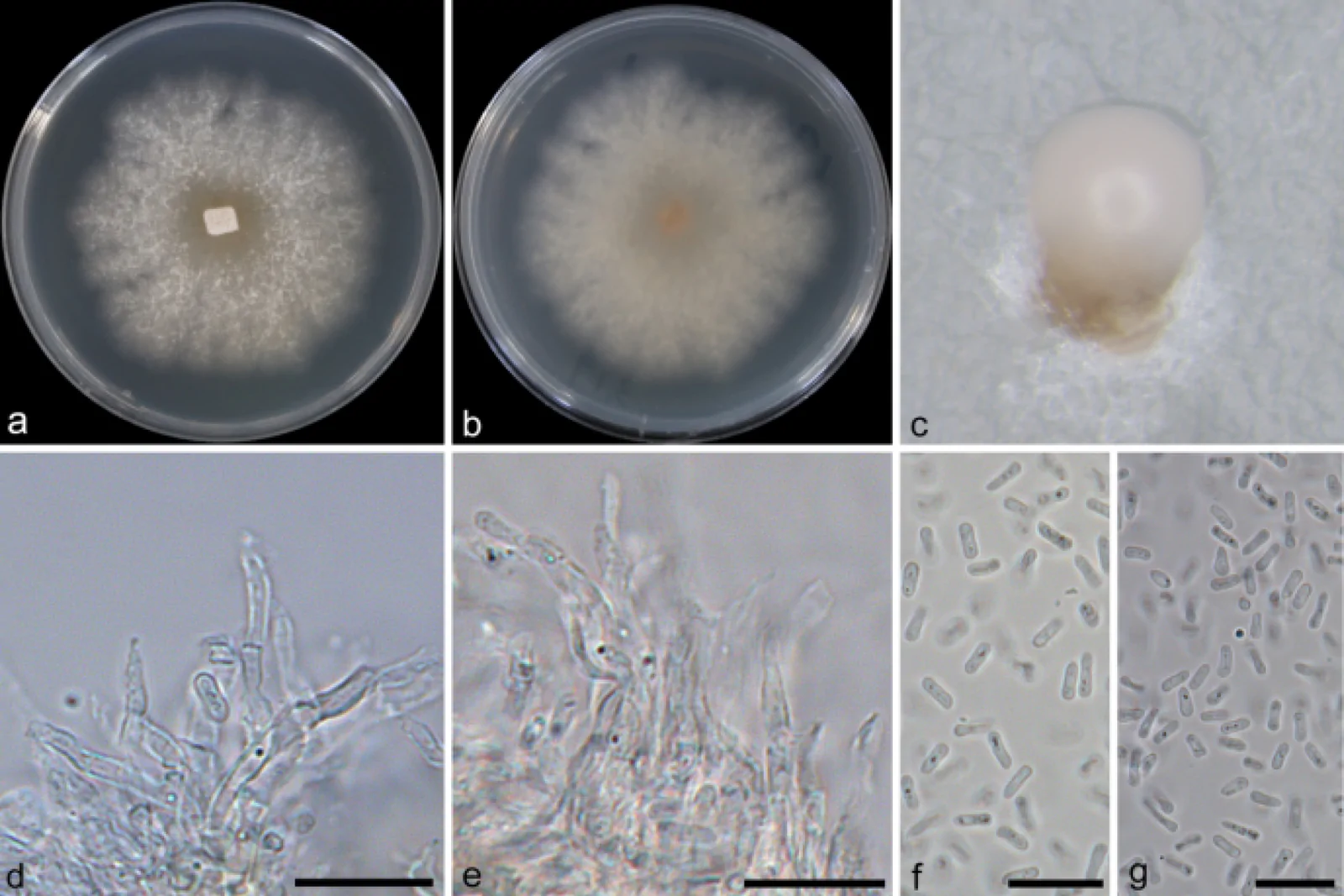
*Gnomoniopsis loropetali* (holotype: HMAS 354535). **a**. Surface of colony after 14 days on PDA; **b**. Reverse side of colony after 14 days on PDA; **c**. Colony overview; **d, e**. Conidiophores with attached conidia; **f, g**. Conidia. Scale bars: 10 µm (**d–g**).

##### Etymology.

The specific epithet *Gnomoniopsis loropetali* is derived from its host plant *Loropetalum chinense*.

##### Description.

Sporodochia are convex or immersed in the substrate, globose or irregularly massive, solitary, light brown. After incubation at 25 °C in darkness for 14 days, abundant whitish, droplet-like conidial masses (containing numerous conidia) exude explosively from the surface. Conidiophores are mostly tubular, straight or curved, tapering upwards from the base, hyaline and smooth-walled, with immature conidia densely arranged at the apex, gradually attenuate toward the tip; 8.4–22.3 × 1.3–2.1 µm (mean = 15.7 × 1.8 µm, L/W = 4.0–17.2 µm, n = 20). Conidia are mostly ovate or partially slender and linear, hyaline, smooth; 3.7–5.5 × 1.2–1.9 µm (mean = 4.3 × 1.5 µm, L/W = 1.9–4.6 µm, n = 25). Sexual morph was not observed.

##### Culture characteristics.

After 14 days of dark incubation at 25 °C on PDA medium, the colony diameter reaches 61.9–67.0 mm with a growth rate of 4.4–4.7 mm per day. The colonies are flat with undulate margins. The inner mycelium is sparse, while the peripheral mycelium is luxuriant. The colony is overall off-white, with pale brown sporodochia scattered irregularly on the surface.

##### Notes.

Phylogenetic analysis based on the combined ITS, *tef1* and *tub2* gene sequences showed that *Gnomoniopsis loropetali* formed an independent clade in the phylogenetic tree, and this clade together with *G. euryae* (CGMCC3.28234) and *G. melastomatis* (CGMCC3.28231) formed a larger clade, indicating that the three taxa are closely related. However, there were certain nucleotide sequence differences among them. The divergences between *G. loropetali* and *G. euryae* are ITS: 46/580 (7.93%), *tef1*: 56/355 (15.77%), *tub2*: 78/416 (18.75%); those between *G. loropetali* and *G. melastomatis* are ITS: 46/577 (7.97%), *tef1*: 57/355 (16.06%), *tub2*: 70/401 (17.46%). Morphologically, the conidiophores of *G. loropetali* were longer than those of *G. euryae* and *G. melastomatis* (8.4–22.3 × 1.3–2.1 µm, L/W = 4.0–17.2 µm vs. 11.3–15.4 × 1.5–1.8 µm, L/W = 6.3–10.3 µm vs. 15–20 × 1.8–2.3 µm, L/W = 6.5–11.1 µm). In contrast, the conidia of *G. loropetali* were shorter and smaller than those of *G. euryae* and *G. melastomatis* (3.7–5.5 × 1.2–1.9 µm, L/W = 1.9–4.6 µm vs. 7–9.5 × 1.8–2.2 µm, L/W = 3.2–5.3 µm vs. 8.5–11 × 2.8–3.8 µm, L/W = 2.2–3.9 µm). The conidia of *G. loropetali* were fusiform or ovoid, whereas those of both *G. euryae* and *G. melastomatis* were fusiform.

#### Gnomoniopsis qixiensis

Taxon classificationAnimaliaDiaporthalesGnomoniaceae

X.S. Wang, Y. Jiang, S. Wang & X.G. Zhang sp. nov.

5C333642-4916-5E67-8D8D-9B96DE349D0E

863467

Fig. 7

##### Holotype.

CHINA • Zhejiang Province, Quzhou City, Kaihua County, Qixi Town, 29°23'25"N, 118°12'35"E, from diseased leaves of *Lindera glauca*, collected on 19 June 2025 by X.S. Wang, holotype HMAS 354536, ex-type living culture SAUCC1995302; paratype, ex-paratype living culture SAUCC1995303.

**Figure 7.**
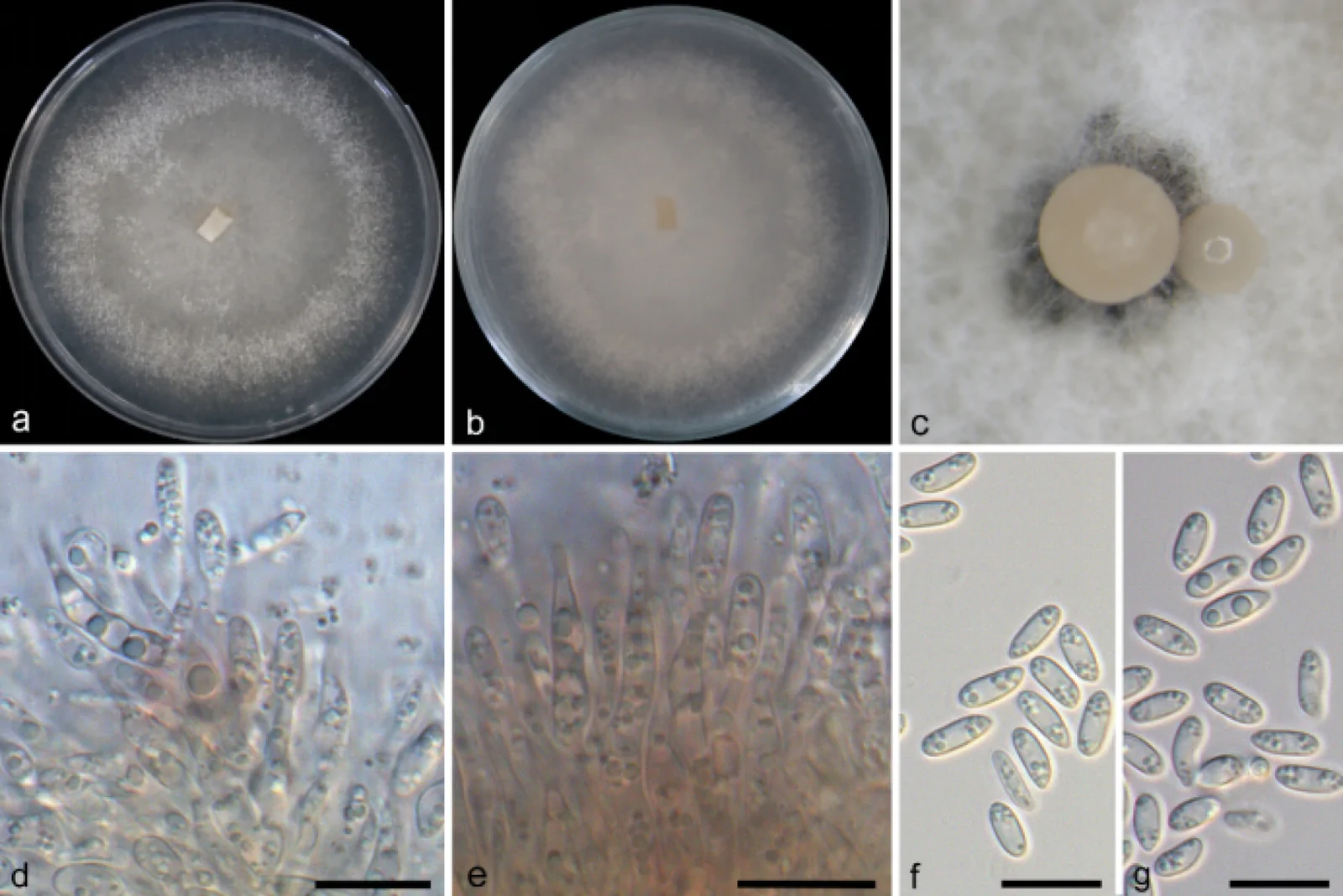
*Gnomoniopsis qixiensis* (holotype: HMAS 354536). **a**. Surface of colony after 14 days on PDA; **b**. Reverse side of colony after 14 days on PDA; **c**. Colony overview; **d, e**. Conidiophores with attached conidia; **f, g**. Conidia. Scale bars: 10 µm (**d–g**).

##### Etymology.

The specific epithet “*qixiensis*” is derived from Qixi Town, Zhe­jiang Province, where the species was collected.

##### Description.

Sporodochia are convex, globose or irregularly massive, gregarious or solitary, black, exuding pale yellow droplet-like conidial masses which are viscous. Conidiophores are mostly tubular, tapering upwards from the base, hyaline and smooth-walled, with immature conidia densely arranged at the apex, gradually attenuate toward the tip; measuring 7.8–33.5 × 1.6–3.2 µm (mean = 20.6 × 2.6 µm, L/W = 2.4–20.9 µm, n = 20). Conidia are ovate or fusiform, hyaline, smooth, and some are slightly curved; measuring 5.5–7.9 × 2.5–3.3 µm (mean = 6.8 × 3.0 µm, L/W = 1.7–3.2 µm, n = 25). Sexual morph was not observed.

##### Culture characteristics.

After 14 days of dark incubation at 25 °C on PDA medium, the colony diameter reaches 78.0–80.5 mm, with a growth rate of 5.5–5.7 mm per day. The colonies are flat with undulate margins. The peripheral mycelium grows luxuriant; the colony is overall off-white, with black sporodochia scattered irregularly on the colony surface.

##### Notes.

Phylogenetic analysis based on the combined ITS, *tef1*, and *tub2* sequences showed that *Gnomoniopsis qixiensis* and *G. yonganensis* (SAUCC1167501) form an independent clade in the phylogenetic tree and have a close phylogenetic relationship. The nucleotide differences between the two species are as follows: 32/565 (5.66%) in the ITS region, 55/330 (16.67%) in *tef1*, and 52/504 (10.32%) in *tub2*. There are significant morphological differences between them: the conidiophores of *G. qixiensis* are short and broad, while those of *G. yonganensis* are long and slender (7.8–33.5 × 1.6–3.2 µm, L/W = 2.4–20.9 µm vs. 12.6–45.9 × 0.9–2.2 µm, L/W = 5.7–51.0 µm). The conidia of *G. qixiensis* are ovate, whereas those of *G. yonganensis* are crescent-shaped.

#### Gnomoniopsis quercus

Taxon classificationAnimaliaDiaporthalesGnomoniaceae

X.S. Wang, Y. Jiang, S. Wang & X.G. Zhang sp. nov.

65D9ACB5-413D-5ED4-A4F6-1E2AE0CA040D

860861

Fig. 8

##### Holotype.

CHINA • Fujian Province, Nanping City, Yanping District, Mangdang Mountain, 26°41'48"N, 118°7'22"E, from diseased leaves of *Quercus griffithii*, 22 October 2024, X.S. Wang, holotype HMAS 354065, ex-type living culture SAUCC1204202a; paratype, ex-paratype living culture SAUCC1204202b.

**Figure 8.**
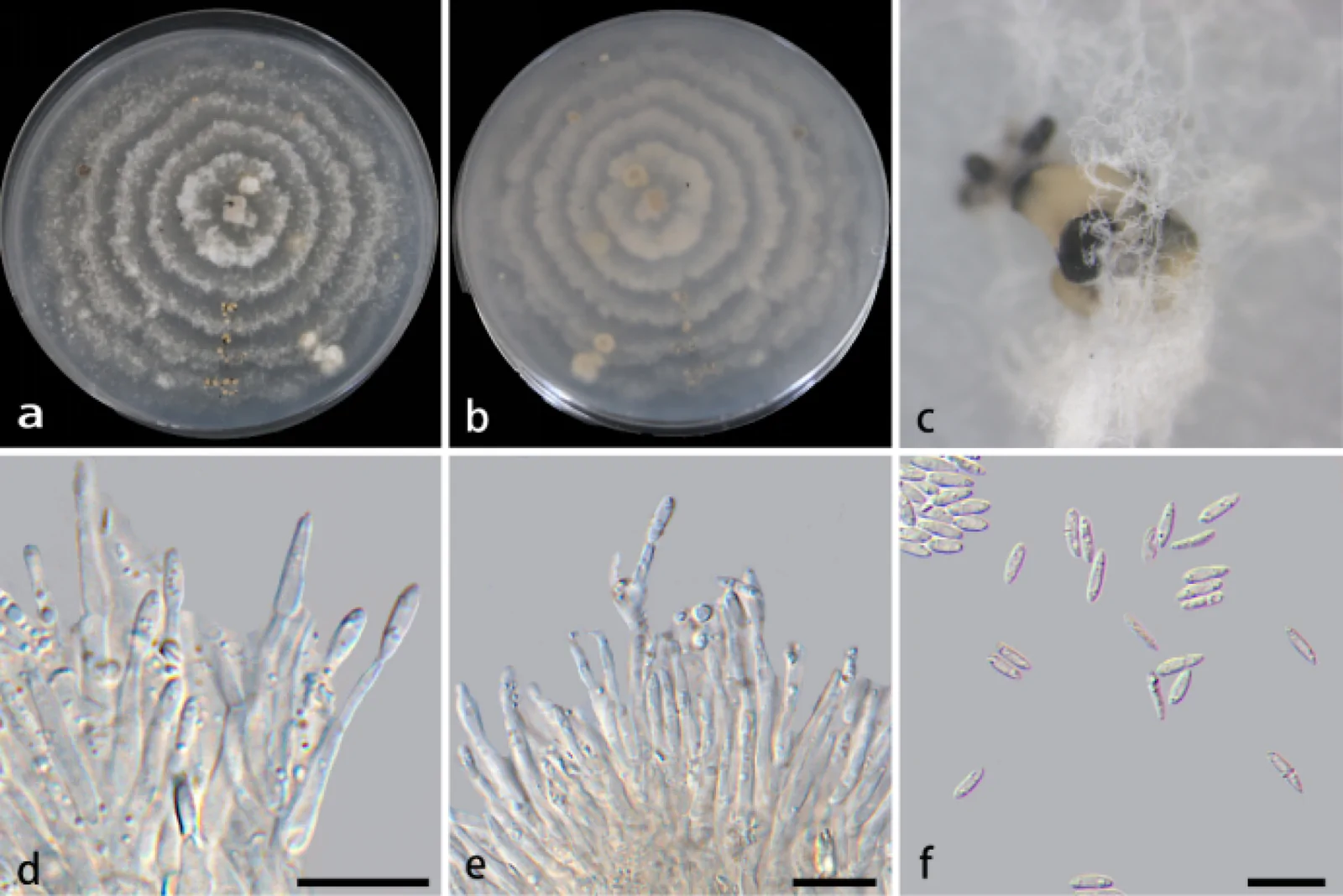
*Gnomoniopsis quercus* (holotype: HMAS 354065). **a**. Surface of colony after 14 days on PDA; **b**. Reverse side of colony after 14 days on PDA; **c**. Colony overview; **d, e**. Conidiophores with attached conidia; **f**. Conidia. Scale bars: 10 µm (**d–f**).

##### Etymology.

The term “*quercus*” is named after its host, *Quercus griffithii*.

##### Description.

The sporodochium protrudes, forming clumpy or irregular columnar structures. They are mostly solitary, with some in clusters, and are black in color. When cultured in a 25°C incubator under dark conditions for 4 to 7 days, a pale yellow, viscous, cream-like aggregate of conidia is emitted on the surface of the sporodochium. Conidiophores are columnar, tapering gradually from the base to the apex, with conidia attached to the top. The cells are colorless and transparent, distributed on the surface of the sporocarp in a dense arrangement 14.0–36.4 × 1.5–3.4 µm (mean = 25.2 × 2.4 µm, L/W = 4.1–24.3 µm, n = 20). Conidia are fusiform or spindle-shaped, transparent and colorless, with a diameter of 6.0–6.9 × 1.8–2.3 µm (mean = 6.5 × 2.1 µm, L/W = 2.6–3.8 µm, n = 25). Sexual morph was not observed.

##### Culture characteristics.

After 14 days of cultivation on PDA plates at 25°C in the dark, the colony diameter is 78.4–83.5 mm, with a growth rate of 5.6–5.9 mm per day. The colony is flat; most of the hyphae grow in a regular wavy pattern, and each circle of waves is covered with white villi, giving the colony an overall grayish-white appearance. The conidiomata are distributed linearly from the center to the periphery of the colony, while a small portion of the hyphae are distributed in clumps.

##### Notes.

Phylogenetic analysis revealed that the newly proposed *Gnomoniopsis quercus* in this study forms a distinct clade (97%/1) together with *G. yunnanensis* (SAUCC YN1659) and *G. xishuangbannaensis* (CGMCC3.25980), indicating their close phylogenetic relationship; however, nucleotide sequence differences exist between *G. quercus* and *G. yunnanensis*, ITS: 4/506 (0.79%); *tef1*: 20/547 (3.66%); *tub2*: 5/464 (1.08%) as well as between *G. quercus* and *G. xishuangbannaensis*, ITS: 12/555 (2.16%); *tef1*: 20/636 (3.14%); *tub2*: 6/499 (1.20%); Morphologically, the conidiophores of *G. quercus* are longer than those of both *G. yunnanensis* and *G. xishuangbannaensis* (14.0–36.4 × 1.5–3.4 µm, L/W = 4.1–24.3 µm vs. 9.0–18.0 × 0.5–1.57 µm, L/W = 5.7–36.0 µm vs. 7–16 × 1.3–2 µm, L/W = 3.5–12.3 µm), the conidia of *G. quercus* are fusiform while those of *G. yunnanensis* and *G. xishuangbannaensis* are ovoid or cylindrical, and the conidia of *G. quercus* are larger than those of *G. yunnanensis* and *G. xishuangbannaensis* (6.0–6.9 × 1.8–2.3 µm, L/W = 2.6–3.8 µm vs. 4.1–5.5 × 1.3–2.0 µm, L/W = 2.1–4.2 µm vs. 5–7 × 1.6–2.6 µm, L/W = 1.9–4.4 µm); in terms of morphological characteristics, the hyphae of *G. quercus* grow in sparse, multilayered undulating layers from the inside out, while the hyphae of *G. yunnanensis* grow in an abundant petal-like spreading pattern and those of *G. xishuangbannaensis* are undulating and abundant.

#### Gnomoniopsis xiaoheijiangensis

Taxon classificationAnimaliaDiaporthalesGnomoniaceae

X.S. Wang, Y. Jiang, S. Wang & X.G. Zhang sp. nov.

289952A8-476F-5897-A7E8-2ED30EEA5D19

863464

Fig. 9

##### Holotype.

CHINA • Yunnan Province, Pu’er City, Ning’er Hani and Yi Autonomous County, Xiaoheijiang Forest Park, 23°12'21"N, 100°55'31"E, from diseased leaves of *Castanea mollissima*, 16 May 2024, X.S. Wang, holotype HMAS 354533, ex-type living culture SAUCC921604; paratype, ex-paratype living culture SAUCC921605.

**Figure 9.**
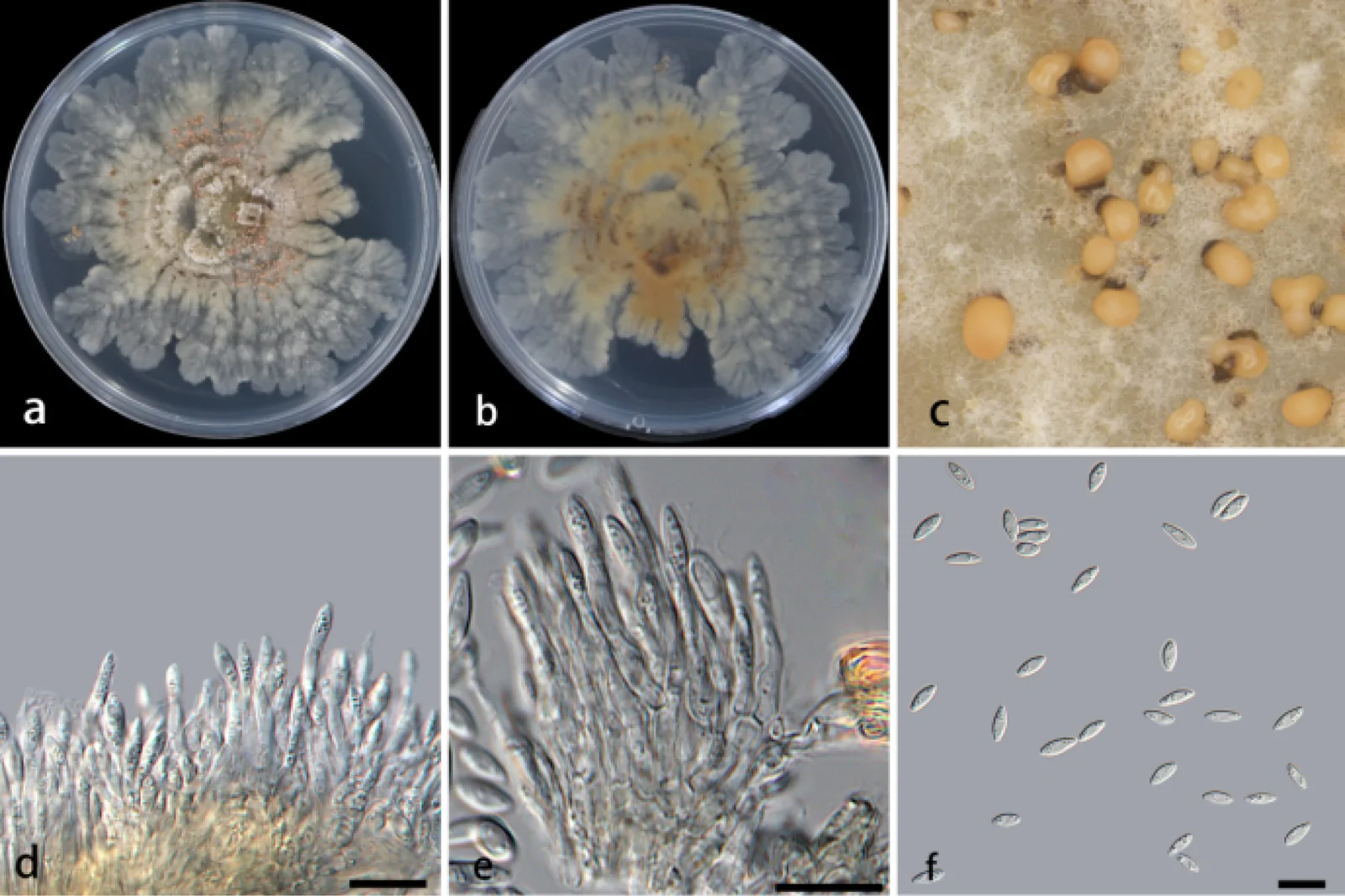
*Gnomoniopsis xiaoheijiangensis* (holotype: HMAS 354533). **a**. Surface of colony after 14 days on PDA; **b**. Reverse side of colony after 14 days on PDA; **c**. Colony overview; **d, e**. Conidiophores with attached conidia; **f**. Conidia. Scale bars: 10 µm (**d–f**).

##### Etymology.

The specific epithet “*xiaoheijiangensis*” is derived from Xiaoheijiang Forest Park, the type locality where this fungal species was collected.

##### Description.

Sporodochia convex, globose or irregularly massive, gregarious or solitary, dark brown, exuding pale yellow droplet-shaped slimy conidial masses. Conidiophores mostly tubular, tapering upwards from the base, hyaline and smooth-walled, bearing immature conidia densely arranged at the apex, gradually attenuate toward the tip; 5.2–16.7 × 1.1–2.4 µm (mean = 10.8 × 2.1 µm, L/W = 2.2–15.2 µm, n = 20). Conidia ovate or fusiform, hyaline, smooth, slightly curved in part; 5.9–8.5 × 2.8–3.7 µm (mean = 7.2 × 3.3 µm, L/W = 1.6–3.0 µm, n = 25). Sexual morph was not observed.

##### Culture characteristics.

After incubation on PDA at 25 °C in darkness for 14 days, the colony diameter reaches 53.4–80.8 mm, with a growth rate of 3.8–5.7 mm per day. Colonies flat, with undulate margins. Mycelium uniformly growing, predominantly off-white; pale brown sporodochia scattered in the central area of colonies.

##### Notes.

Phylogenetic analyses based on concatenated ITS, *tef1* and *tub2* sequences revealed that *Gnomoniopsis xiaoheijiangensis* forms a distinct clade with *G. rossmaniae* (CFCC 54307), indicating a close phylogenetic relationship. The nucleotide differences between the two species are as follows: ITS: 49/507 (9.66%), *tef1*: 109/176 (61.93%), *tub2*: 44/374 (11.76%). Morphologically, the conidiophores of *G. xiaoheijiangensis* are smaller than those of *G. rossmaniae* (5.2–16.7 × 1.1–2.4 µm, L/W = 2.2–15.2 µm vs. 9.0–19.0 × 2.0–3.0 µm, L/W = 3.0–9.5 µm), and the conidia of *G. xiaoheijiangensis* are distinctly shorter (5.9–8.5 × 2.8–3.7 µm, L/W = 1.6–3.0 µm vs. 11.6–14.6 × 3.3–3.9 µm, L/W = 2.9–4.4 µm). In addition, *G. xiaoheijiangensis* produces more abundant sporodochia and exudes more conidial masses compared with *G. rossmaniae*.

#### Gnomoniopsis yonganensis

Taxon classificationAnimaliaDiaporthalesGnomoniaceae

X.S. Wang, Y. Jiang, S. Wang & X.G. Zhang sp. nov.

1C42FB42-39CB-56E2-93F3-7AD284754EB2

860860

Fig. 10

##### Holotype.

CHINA • Fujian Province, Sanming City, Yong’an City, Taoyuan Cave beside National Highway 205, 26°1'29"N, 117°24'55"E, from *Lindera communis*, 23 October 2024, X.S. Wang, holotype HMAS 354064, ex-type living culture SAUCC1167501; paratype, ex-paratype living culture SAUCC1167502.

**Figure 10.**
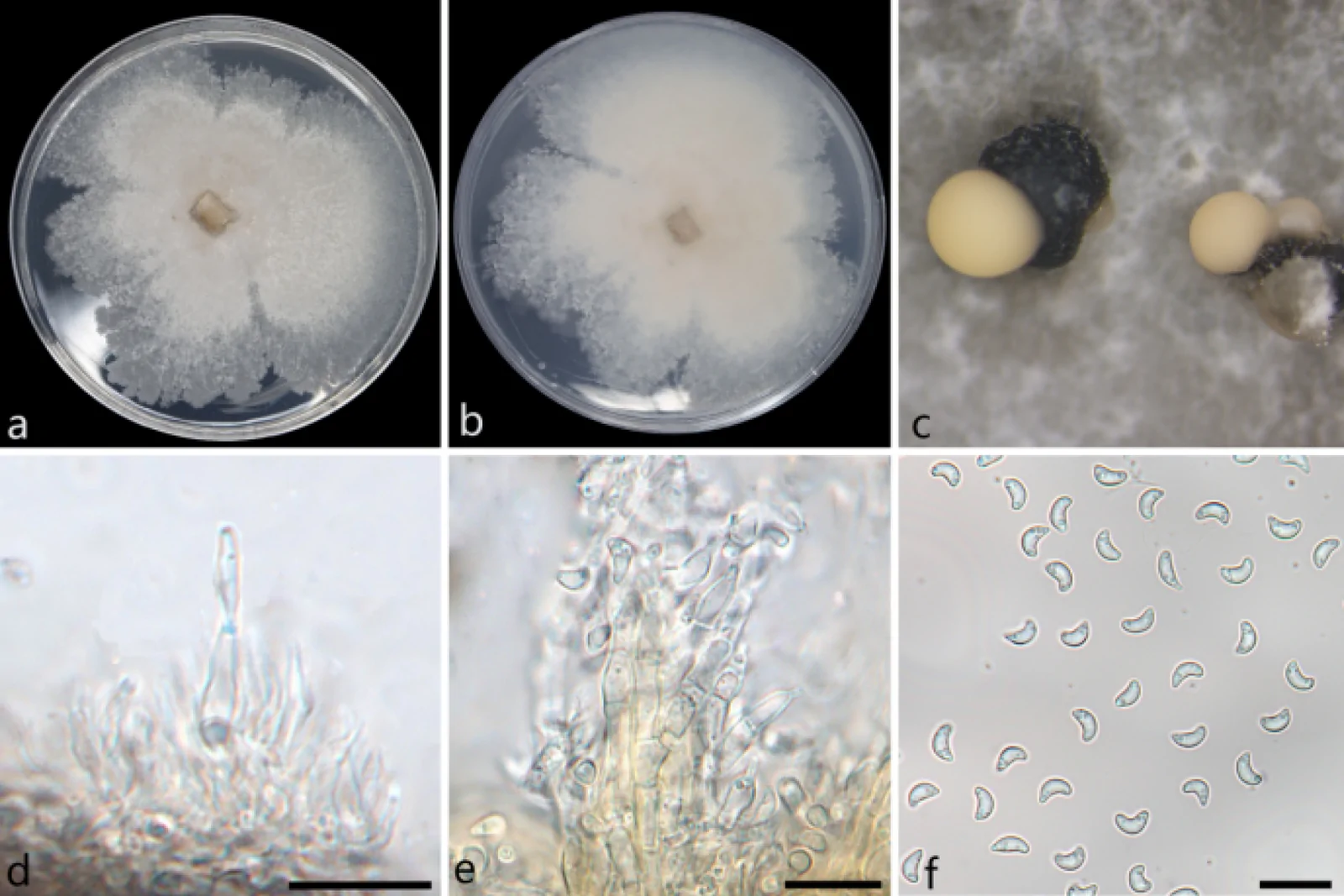
*Gnomoniopsis yonganensis* (holotype: HMAS 354064). **a**. Surface of colony after 14 days on PDA; **b**. Reverse side of colony after 14 days on PDA; **c**. Colony overview; **d, e**. Conidiophores with attached conidia; **f**. Conidia. Scale bars: 10 µm (**d–f**).

##### Etymology.

The specific epithet of this species is named after Yong’an City, the locality where its host plant was collected.

##### Description.

The sporodochia appear as massive or spherical protrusions, distributed irregularly singly on the colony. And they are black when mature and light brown in the early stage; after being cultivated in a dark environment at 25°C for 14 days, pale yellow, viscous and cream-like conidia aggregates oozed from the surface. Conidiophores are irregular columnar, gradually narrowing from the base to the top, arranged in clusters on the surface of sporodochium. Conidia are mostly attached to the top, with some in the middle section, and have a septum inside. They are transparent and colorless 12.6–45.9 × 0.9–2.2 µm (mean = 29.2 × 1.5 µm, L/W = 5.7–51.0 µm, n = 20). Conidia are crescent-shaped, transparent and colorless, and smooth 4.8–6.0 × 1.8–2.4 µm (mean = 5.5 × 2.2 µm, L/W = 2.0–3.3 µm, n = 25). Sexual morph was not observed.

##### Culture characteristics.

After 14 days of cultivation in the dark condition at 25°C, the diameter of the colony on PDA reached 65.2–83.6 mm, and the growth rate was 4.6–5.9 mm per day. The colony was gray and white, with flat growth. The hyphae were short and embedded on PDA, and the sporophytes were scattered irregularly in a few numbers.

##### Notes.

Phylogenetic analyses based on the multi-locus sequences of ITS, *tef1* and *tub2* revealed that *Gnomoniopsis yonganensis* and *G. qixiensis* (SAUCC1995302) formed a distinct clade with a close phylogenetic relationship. The nucleotide differences between the two species were 32/565 (5.66%) in the ITS region, 55/330 (16.67%) in *tef1*, and 52/504 (10.32%) in *tub2*. Significant morphological differences were observed between them. The conidiophores of *G. yonganensis* are long and slender, whereas those of *G. qixiensis* are shorter and broader (12.6–45.9 × 0.9–2.2 µm, L/W = 5.7–51.0 µm vs. 7.8–33.5 × 1.6–3.2 µm, L/W = 2.4–20.9 µm). The conidia of *G. yonganensis* are lunate, while those of *G. qixiensis* are ovate.

#### Gnomoniopsis yunvfengensis

Taxon classificationAnimaliaDiaporthalesGnomoniaceae

X.S. Wang, Y. Jiang, S. Wang & X.G. Zhang sp. nov.

D918EBAA-1D47-5502-9E17-0EB882FB98D1

860859

Fig. 11

##### Holotype.

CHINA • Fujian Province, Nanping City, Wuyishan City, Township Road 234, Yunv Peak, 27°38'33"N, 117°57'19"E, from decaying leaves, 21 October 2024, X.S. Wang, holotype HMAS 354063, ex-type living culture SAUCC1132501; paratype, ex-paratype living culture SAUCC1132502.

**Figure 11.**
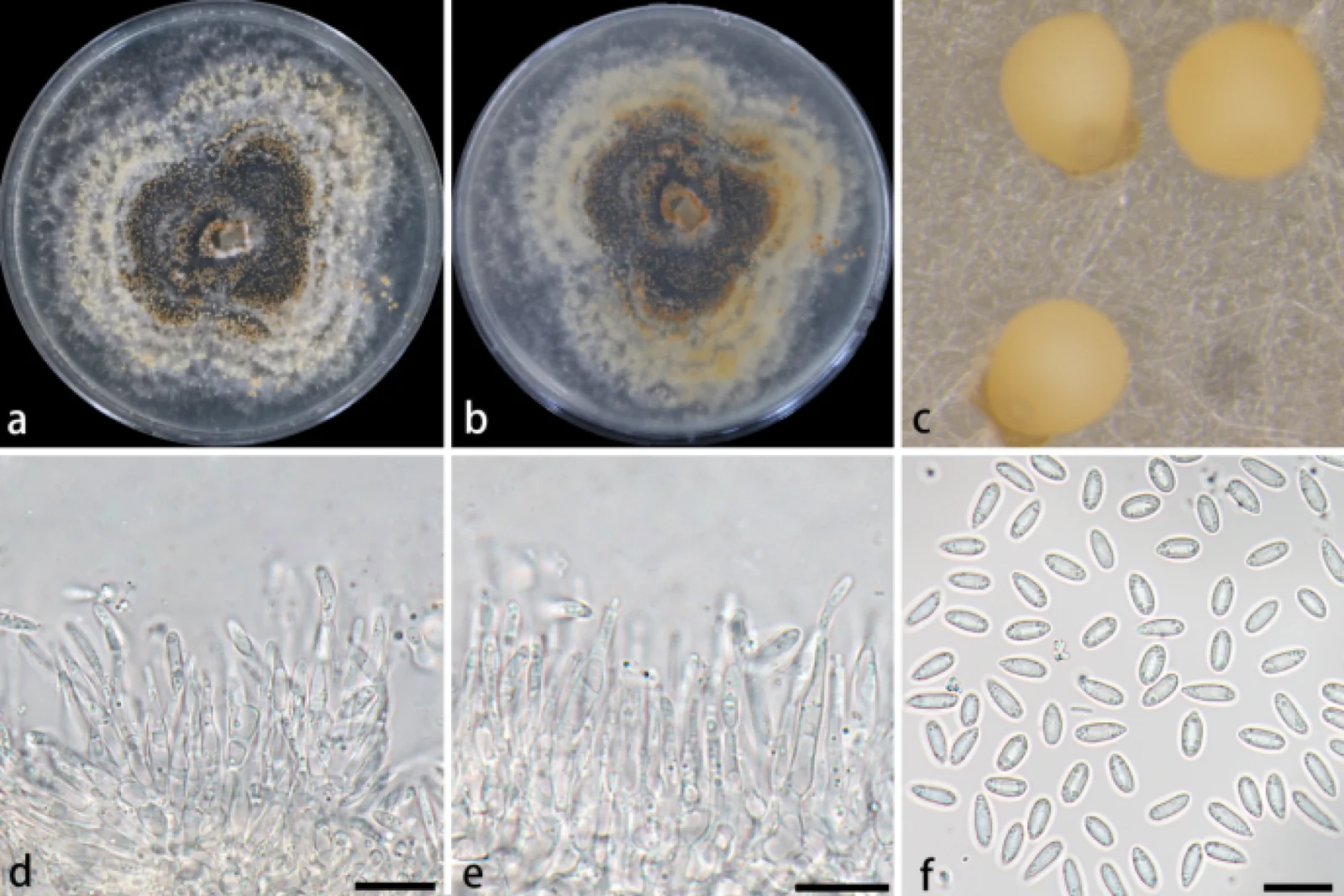
*Gnomoniopsis yunvfengensis* (holotype: HMAS 354063). **a**. Surface of colony after 14 days on PDA; **b**. Reverse side of colony after 14 days on PDA; **c**. Colony overview; **d, e**. Conidiophores with attached conidia; **f**. Conidia. Scale bars: 10 µm (**d–f**).

##### Etymology.

The term “*yunvfengensis*” comes from the host collection site – Yunv Peak.

##### Description.

Sporodochium protrudes and forms blocky or spherical shapes. Most of them are solitary while some are clustered. They are light brown. After being cultivated in a dark environment at 25°C for 4 to 7 days, the surface of the carrier spores exudes yellow, viscous, cream-like secretions, which contain conidia. Conidiophores are tubular and gradually narrow from the bottom upwards. The cells are colorless and transparent, distributed on the surface of the carrier body and closely clustered 10.4–28.5 × 1.2–2.3 µm (mean = 19.4 × 1.7 µm, L/W = 4.5–23.8 µm, n = 20). Conidia are spindle-shaped, transparent and colorless 5.4–8.4 × 1.9–3.0 µm (mean = 6.6 × 2.4 µm, L/W = 1.8–4.4 µm, n = 25). Sexual morph was not observed.

##### Culture characteristics.

After 14 days of cultivation at 25°C in the dark, the colony reaches a diameter of 78.1–83.2 mm, with a growth rate of 5.5–5.9 mm per day. The colony is flat and exhibits irregular wavy growth from the center to the periphery, presenting an overall grayish-white color. Light brown conidiomata are irregularly distributed in the inner part of the colony.

##### Notes.

Phylogenetic analysis of the ITS, *tef1* and *tub2* sequences indicates that *Gnomoniopsis yunvfengensis* is closely related to *G. mengyinensis* (SAUCC MY0293) and *G. flava* (CFCC 71567). In detail, *G. yunvfengensis* is distinguished from *G. mengyinensis* by 13/510 (2.55%), 68/551 (12.34%) and 30/465 (6.45%) characters in ITS, *tef1* and *tub2* sequences, respectively. In the same, it is distinguished from *G. flava* sp. nov. by 43/551 (7.80%), 92/550 (16.73%) and 45/465 (9.68%) characters in ITS, *tef1* and *tub2* sequences, respectively. Morphologically, the conidiophores of *G. yunvfengensis* are longer than those of *G. mengyinensis* and *G. flava* (10.4–28.5 × 1.2–2.3 µm, L/W = 4.5–23.8 µm vs. 8.0–11.5 × 1.3–2.2 µm, L/W = 3.6–8.8 µm vs. 11.5–26 × 1.5–2.5 µm, L/W = 4.6–17.3 µm). *G. yunvfengensis* and *G. flava* have fewer hyphae and their colonies grow relatively irregularly, while *G. mengyinensis* has vigorously growing hyphae.

### Pathogenicity tests

**Figure 12.**
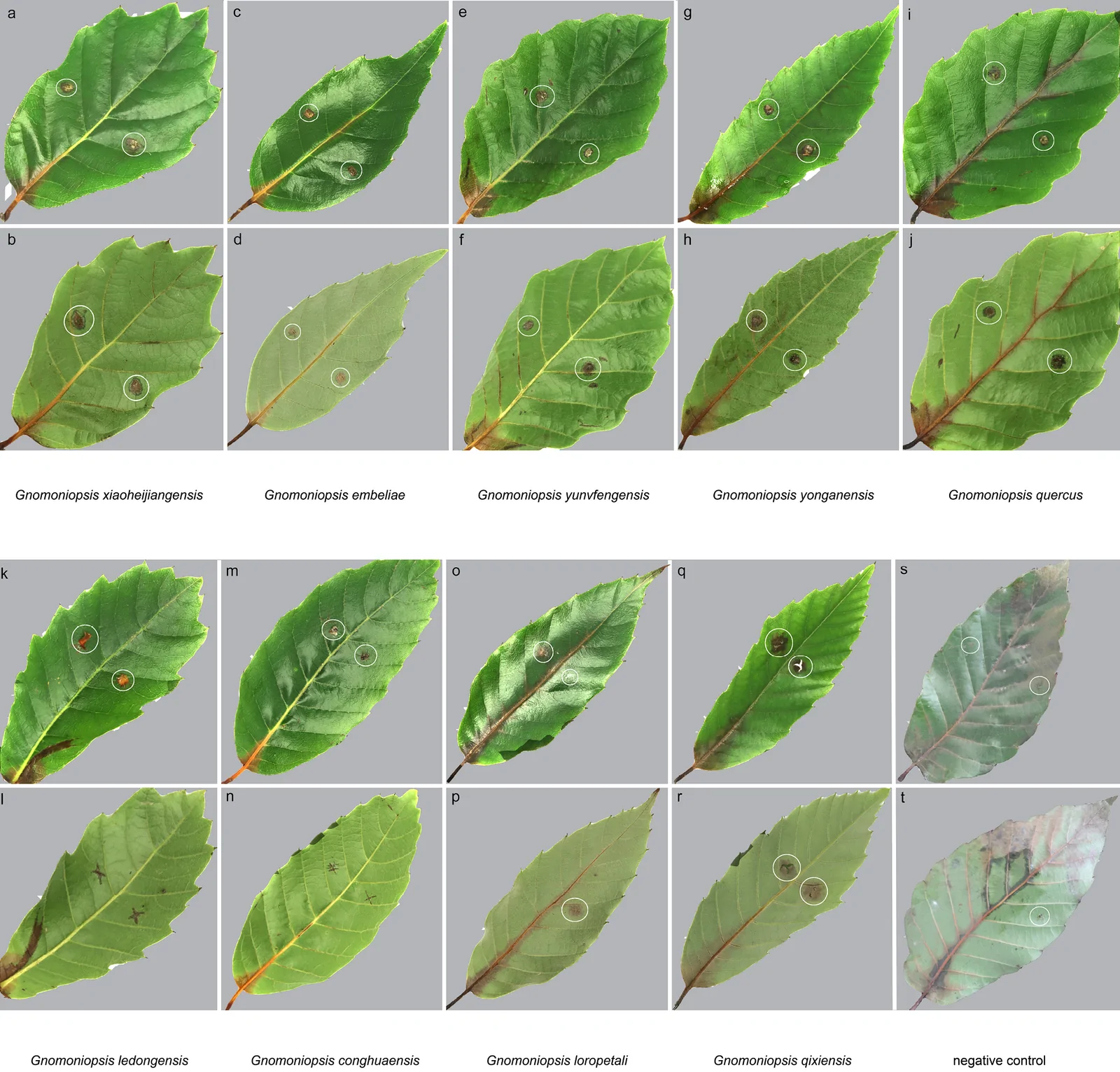
Pathogenicity symptoms on detached healthy *Castanea mollissima* leaves inoculated with nine novel *Gnomoniopsis* species and negative control. **a, b**. *Gnomoniopsis xiaoheijiangensis*; **c, d**. *G. embeliae*; **e, f**. *G. yunvfengensis*; **g, h**. *G. yonganensis*; **i, j**. *G. quercus*; **k, l**. *G. ledongensis*; **m, n**. *G. conghuaensis*; **o, p**. *G. loropetali*; **q, r**. *G. qixiensis*; **s, t**. Negative control leaves treated with blank PDA plugs without fungal mycelium. All leaf tissues were artificially wounded before inoculation, and obvious necrotic lesions formed at the inoculation sites 3–4 days post-incubation for all nine fungal taxa; no disease lesions were observed on negative control foliage. Circles mark the positions of inoculation wounds and resultant necrotic lesions.

Pathogenicity inoculation experiments demonstrated (Fig. 12) that all nine novel *Gnomoniopsis* isolates were capable of triggering necrotic lesions on wounded *Castanea mollissima* (chestnut, *Fagaceae*) leaf tissue. This assay was designed to evaluate the general necrotrophic capacity of these fungi on *Fagaceae* tissue, rather than host-specific pathogenicity against their original host plants. Initial faint brown discoloration emerged around the inoculation wound site 3–4 days after treatment, and these discolored areas gradually expanded outward to form distinct water-soaked necrotic plaques as incubation time extended. No abnormal discoloration or tissue rot symptoms were detected on any leaves belonging to the blank negative control group throughout the entire observation period.

Fungal isolation was conducted on symptomatic leaf fragments to complete Koch’s postulate verification. Fungal colonies retrieved from lesion margins exhibited colony morphologies fully consistent with the corresponding original test strains. Sequence alignment of three standard molecular markers (ITS, *tef1*, *tub2*) further confirmed that the reisolated fungal strains shared 99%–100% sequence identity with the strains initially used for inoculation. Combined phenotypic and molecular evidence satisfied the four core requirements of Koch’s postulates under this experimental condition, verifying that all nine new *Gnomoniopsis* species possess the capacity to induce leaf necrotic symptoms on wounded chestnut foliage.

## Discussion

Rapid development of genomic technologies has advanced traditional fungal taxonomy from qualitative morphological description to an integrated “phenotype–genotype–pan–genome landscape” paradigm (McCarthy and Fitzpatrick 2019; Xu 2020; Stengel et al. 2022). As a key genus in *Diaporthales* (*Ascomycota*), *Gnomoniopsis* has long suffered taxonomic ambiguities. Most species in this group are mainly endophytes and phytopathogens associated with *Fagaceae*, *Rosaceae* and related hosts plants. They display highly overlapping morphological characteristics and variable spore sizes, which renders traditional morphological identification unreliable for distinguishing closely related taxa. Although multi-locus analyses based on ITS, *tef1* and *tub2* have become the mainstream strategy, insufficient genetic resolution still limits the discovery of cryptic species (Rossman et al. 2007; Visentin et al. 2012; Walker 2012; Jiang et al. 2021b; Turco et al. 2023). By providing in-depth genomic data such as SNPs and gene family expansion or contraction through whole-genome sequencing, genomic research solidifies the systematic revision of *Gnomoniopsis*, and establishes reliable molecular criteria for species delineation (Grünwald et al. 2016; Kulik et al. 2022; Merényi et al. 2023).

After phylogenetic analyses, we identified and described nine new species of the genus *Gnomoniopsis* collected from five Chinese provinces, which were isolated from six different host plants: four hosts belonging to the *Fagaceae*, two to the genus *Lindera (Lauraceae)*, and one to the *Primulaceae*. Notably, two novel species, *G. yunvfengensis* and *G. conghuaensis*, were obtained from decaying leaves without clear host information. These findings greatly extend the known host range and ecological distribution of *Gnomoniopsis*, and confirm that the host diversity and lifestyle plasticity of this genus are far more complex than previously documented, further implying that numerous cryptic species and unexplored host associations remain to be discovered in less-investigated plant lineages and unique microhabitats. Moreover, the newly generated genome data of the nine species significantly supplement the current genomic resources of *Gnomoniopsis*, providing valuable molecular evidence and a fundamental dataset for future phylogenetic reconstruction, adaptive evolution analysis, and systematic taxonomic revision of this genus.

Based on phylogenetic phylogenomic tree construction (Wisecaver et al. 2014), this study reveals the evolutionary relationships of *Gnomoniopsis*. These evolutionary patterns are highly consistent with well-supported conclusions from previous fungal evolutionary studies. Phylogenomic analyses showed that *G. castaneae* and *G. smithogilvyi* (IMI 355082) clustered in the same clade. Their evolutionary relationships were consistent with multi-locus phylogenetic trees and highly congruent with the phylogenetic relationships reported by Lizcano Salas et al. (Lizcano Salas et al. 2024) in their study on *Fusarium*. These results indicate that phylogenomics, especially based on single-copy orthologous genes, is universally applicable in resolving ambiguous species boundaries in fungi, and further clarify the taxonomic boundaries among species within the genus *Gnomoniopsis* (Feau et al. 2011; Gostinčar 2020; Gomez-Chavarria et al. 2024; Sarawad et al. 2025).

## Conclusions

This study characterizes the genomic genetic features of *Gnomoniopsis* strains through whole-genome sequencing. Phylogenomic results provide supplementary evidence to previous taxonomic investigations that relied solely on a limited set of barcode loci. For future species delimitation and evolutionary analysis of this genus, analytical frameworks based on multi-locus phylogeny or concatenated phylogenomic datasets are preferable to single-gene approaches. Single-locus phylogenetic methods usually lack sufficient resolution to distinguish genetically close lineages, and may produce clustering results inconsistent with the robust species boundaries supported by morphological observation, pathogenicity verification and PHI recombination tests. The genomic resources generated in this work can serve as fundamental reference data to resolve ambiguous taxonomic relationships within *Gnomoniopsis*. No definitive evolutionary or species-delimitation hypothesis is put forward based solely on current genomic data; further research with expanded sampling, combined with morphological comparison and physiological experimental validation, is required to optimize the identification system for species of this genus.

## Supplementary Material

XML Treatment for Gnomoniopsis conghuaensis

XML Treatment for Gnomoniopsis embeliae

XML Treatment for Gnomoniopsis ledongensis

XML Treatment for Gnomoniopsis loropetali

XML Treatment for Gnomoniopsis qixiensis

XML Treatment for Gnomoniopsis quercus

XML Treatment for Gnomoniopsis xiaoheijiangensis

XML Treatment for Gnomoniopsis yonganensis

XML Treatment for Gnomoniopsis yunvfengensis

## Additional information

### Artificial Intelligence (AI) use

The authors accept full responsibility for the content of the manuscript, including the disclosure of any use of AI.

No AI tools were used in the preparation of this manuscript.

### Adherence to national and international regulations

All the fungal strains used in this study have been legally obtained, respecting the Convention on Biological Diversity (Rio Convention).

### Author ORCIDs

X. Wang https://orcid.org/0009-0005-1127-7368

Y. Jiang https://orcid.org/0009-0003-1292-610X

W. Liu https://orcid.org/0009-0009-3477-194X

X. Yan https://orcid.org/0009-0005-6257-3490

J. Sheng https://orcid.org/0009-0003-3882-6748

C. Ai https://orcid.org/0009-0002-3934-7944

X. Liu https://orcid.org/0000-0002-8808-010X

X. Zhang https://orcid.org/0000-0001-9733-8494

Z. Meng https://orcid.org/0000-0001-9085-1324

S. Wang https://orcid.org/0000-0002-7376-7638

### Data availability

All of the data that support the findings of this study are available in the main text or Supplementary Information.

## Supplementary materials

Supplementary material 1 Suppl. data 1–4: the sequences after MEGA for *Gnomoniopsis* Data type zip Explanation note **suppl. data 1**. The ITS sequences for *Gnomoniopsis* after MEGA. **suppl. data 2**. The *tef1* sequences for *Gnomoniopsis* after MEGA. **suppl. data 3**. The *tub2* sequences for *Gnomoniopsis* after MEGA. **suppl. data 4**. The ITS–*tef1*–*tub2* sequences for *Gnomoniopsis* after MEGA. https://binary.pensoft.net/file/1817823This dataset is made available under the Open Database License (http://opendatacommons.org/licenses/odbl/1.0/). The Open Database License (ODbL) is a license agreement intended to allow users to freely share, modify, and use this Dataset while maintaining this same freedom for others, provided that the original source and author(s) are credited. Xingsheng Wang, Yang Jiang, Wenwen Liu, Xigang Yan, Jinyan Sheng, Congcong Ai, Xiaoyong Liu, Xiuguo Zhang, Zhe Meng, Shi Wang

Supplementary material 2 Single gene trees for *Gnomoniopsis* Data type zip Explanation note **fig. S1**. Phylogenetic trees constructed using Maximum likelihood (ML) analyses based on ITS for *Gnomoniopsis*. **fig. S2**. Phylogenetic trees constructed using Maximum likelihood (ML) analyses based on *tef1* for *Gnomoniopsis*. **fig. S3**. Phylogenetic trees constructed using Maximum likelihood (ML) analyses based on *tub2* for *Gnomoniopsis*. https://binary.pensoft.net/file/1817824This dataset is made available under the Open Database License (http://opendatacommons.org/licenses/odbl/1.0/). The Open Database License (ODbL) is a license agreement intended to allow users to freely share, modify, and use this Dataset while maintaining this same freedom for others, provided that the original source and author(s) are credited. Xingsheng Wang, Yang Jiang, Wenwen Liu, Xigang Yan, Jinyan Sheng, Congcong Ai, Xiaoyong Liu, Xiuguo Zhang, Zhe Meng, Shi Wang

Supplementary material 3 PHI species delimitation test for *Gnomoniopsis* Data type zip Explanation note **figures S1–S6** exhibit pairwise homoplasy index (PHI) analytical graphs used to delimit nine novel species within *Gnomoniopsis*. https://binary.pensoft.net/file/1817825This dataset is made available under the Open Database License (http://opendatacommons.org/licenses/odbl/1.0/). The Open Database License (ODbL) is a license agreement intended to allow users to freely share, modify, and use this Dataset while maintaining this same freedom for others, provided that the original source and author(s) are credited. Xingsheng Wang, Yang Jiang, Wenwen Liu, Xigang Yan, Jinyan Sheng, Congcong Ai, Xiaoyong Liu, Xiuguo Zhang, Zhe Meng, Shi Wang

Supplementary material 4 Analysis of secondary metabolite biosynthetic gene clusters of *Gnomoniopsis* Data type zip Explanation note **tables S1–S9** show the detailed analyses of secondary metabolite biosynthetic gene clusters for the nine novel *Gnomoniopsis* species described in this study. https://binary.pensoft.net/file/1817826This dataset is made available under the Open Database License (http://opendatacommons.org/licenses/odbl/1.0/). The Open Database License (ODbL) is a license agreement intended to allow users to freely share, modify, and use this Dataset while maintaining this same freedom for others, provided that the original source and author(s) are credited. Xingsheng Wang, Yang Jiang, Wenwen Liu, Xigang Yan, Jinyan Sheng, Congcong Ai, Xiaoyong Liu, Xiuguo Zhang, Zhe Meng, Shi Wang

## References

[B1] Cantarel BL, Coutinho PM, Rancurel C et al. (2009) The Carbohydrate-Active EnZymes database (CAZy): an expert resource for Glycogenomics. Nucleic Acids Research 37: D233–D238. 10.1093/nar/gkn663 PMC2686590 18838391

[B2] Carbone I, Kohn LM (1999) A method for designing primer sets for speciation studies in filamentous ascomycetes. Mycologia 91: 553–556. 10.1080/00275514.1999.12061051

[B3] Chan PP, Lin BY, Mak AJ et al. (2021) tRNAscan-SE 2.0: improved detection and functional classification of transfer RNA genes. Nucleic Acids Research 49: 9077–9096. 10.1093/nar/gkab688 PMC8450103 34417604

[B4] Chen N (2004) Using RepeatMasker to identify repetitive elements in genomic sequences. Current Protocols in Bioinformatics 5: 4.10.1–4.10.14. 10.1002/0471250953.bi0410s05 19274634

[B5] Chen S (2025) fastp 1.0: An ultra‐fast all‐round tool for FASTQ data quality control and preprocessing. iMeta 4: e70078. 10.1002/imt2.70078 PMC12527978 41112039

[B6] Crous PW, Summerell BA, Shivas RG et al. (2012) Fungal Planet description sheets: 107–127. Persoonia – Molecular Phylogeny and Evolution of Fungi 28: 138–182. 10.3767/003158512X652633 PMC3409410 23105159

[B7] Crous PW, Luangsa-ard JJ, Wingfield MJ et al. (2018) Fungal Planet description sheets: 785–867. Persoonia – Molecular Phylogeny and Evolution of Fungi 41: 238–417. 10.3767/persoonia.2018.41.12 PMC6344811 30728607

[B8] Farrer RA, Martel A, Verbrugghe E et al. (2017) Genomic innovations linked to infection strategies across emerging pathogenic chytrid fungi. Nature Communications 8: 14742. 10.1038/ncomms14742 PMC5364385 28322291

[B9] Feau N, Decourcelle T, Husson C et al. (2011) Finding single copy genes out of sequenced genomes for multilocus phylogenetics in non-model fungi. Butler G (Ed.). PLOS ONE 6: e18803. 10.1371/journal.pone.0018803 PMC3076447 21533204

[B10] Flynn JM, Hubley R, Goubert C et al. (2020) RepeatModeler2 for automated genomic discovery of transposable element families. Proceedings of the National Academy of Sciences 117: 9451–9457. 10.1073/pnas.1921046117 PMC7196820 32300014

[B11] Glass NL, Donaldson GC (1995) Development of primer sets designed for use with the PCR to amplify conserved genes from filamentous ascomycetes. Applied and Environmental Microbiology 61: 1323–1330. 10.1128/aem.61.4.1323-1330.1995 PMC167388 7747954

[B12] Gomez-Chavarria DA, Rua-Giraldo AL, Alzate JF (2024) An evolutionary view of the *Fusarium* core genome. BMC Genomics 25: 304. 10.1186/s12864-024-10200-w PMC10958916 38519886

[B13] Gostinčar C (2020) Towards genomic criteria for delineating fungal species. Journal of Fungi 6: 246. 10.3390/jof6040246 PMC7711752 33114441

[B14] Grünwald NJ, McDonald BA, Milgroom MG (2016) Population genomics of fungal and oomycete pathogens. Annual Review of Phytopathology 54: 323–346. 10.1146/annurev-phyto-080614-115913 27296138

[B15] Guo Z-H, Zhou Y-Q, Wang J et al. (2026) Three new species of *Gnomoniopsis* and *Ternstroemiomyces* (*Diaporthales*, *Sordariomycetes*) from China. MycoKeys 129: 81–105. 10.3897/mycokeys.129.182847 PMC12954450 41783134

[B16] Haas BJ, Zeng Q, Pearson MD et al. (2011) Approaches to fungal genome annotation. Mycology 2: 118–141. 10.1080/21501203.2011.606851 PMC3207268 22059117

[B17] Haas BJ, Salzberg SL, Zhu W et al. (2008) Automated eukaryotic gene structure annotation using EVidenceModeler and the program to assemble spliced alignments. Genome Biology 9: R7. 10.1186/gb-2008-9-1-r7 PMC2395244 18190707

[B18] Huson DH (1998) SplitsTree: analyzing and visualizing evolutionary data. Bioinformatics 14: 68–73. 10.1093/bioinformatics/14.1.68 9520503

[B19] Huson DH, Bryant D (2006) Application of phylogenetic networks in evolutionary studies. Molecular Biology and Evolution 23: 254–267. 10.1093/molbev/msj030 16221896

[B20] Jiang N, Tian C (2019) An emerging pathogen from rotted chestnut in China: *Gnomo­niopsis daii* sp. nov. Forests 10: 1016. 10.3390/f10111016

[B21] Jiang N, Liang L-Y, Tian C-M (2020) *Gnomoniopsis chinensis* (*Gnomoniaceae*, *Diaporthales*), a new fungus causing canker of Chinese chestnut in Hebei Province, China. MycoKeys 67: 19–32. 10.3897/mycokeys.67.51133 PMC7242485 32476980

[B22] Jiang N, Fan X, Tian C (2021a) Identification and characterization of leaf-inhabiting fungi from *Castanea* Plantations in China. Journal of Fungi 7: 64. 10.3390/jof7010064 PMC7831338 33477575

[B23] Jiang N, Xue H, Piao C et al. (2022) Characterization and identification of *Gnomoniopsis* species. Terrestrial Ecosystem and Conservation 2: 44–52. 10.12356/j.2096-8884.2022-0031

[B24] Jiang N, Voglmayr H, Bian D-R et al. (2021b) Morphology and phylogeny of *Gnomoniopsis* (*Gnomoniaceae*, *Diaporthales*) from *Fagaceae* leaves in China. Journal of Fungi 7: 792. 10.3390/jof7100792 PMC8540803 34682214

[B25] Kalvari I, Nawrocki EP, Argasinska J et al. (2018) Non‐coding RNA analysis using the Rfam database. Current Protocols in Bioinformatics 62: e51. 10.1002/cpbi.51 PMC6754622 29927072

[B26] Keilwagen J, Hartung F, Paulini M et al. (2018) Combining RNA-seq data and homology-based gene prediction for plants, animals and fungi. BMC Bioinformatics 19: 189. 10.1186/s12859-018-2203-5 PMC5975413 29843602

[B27] Keilwagen J, Wenk M, Erickson JL et al. (2016) Using intron position conservation for homology-based gene prediction. Nucleic Acids Research 44: e89–e89. 10.1093/nar/gkw092 PMC4872089 26893356

[B28] Keller NP (2019) Fungal secondary metabolism: regulation, function and drug discovery. Nature Reviews Microbiology 17: 167–180. 10.1038/s41579-018-0121-1 PMC6381595 30531948

[B29] Kulik T, Molcan T, Fiedorowicz G et al. (2022) Whole-genome single nucleotide polymorphism analysis for typing the pandemic pathogen *Fusarium graminearum* sensu stricto. Frontiers in Microbiology 13: 885978. 10.3389/fmicb.2022.885978 PMC9339996 35923405

[B30] Lagesen K, Hallin P, Rødland EA et al. (2007) RNAmmer: consistent and rapid annotation of ribosomal RNA genes. Nucleic Acids Research 35: 3100–3108. 10.1093/nar/gkm160 PMC1888812 17452365

[B31] Li Y, Li A, Jiang N (2025) Identification of the nut rot pathogen affecting *Castanopsis carlesii* Based on morphological and phylogenetic analyses. Forests 16: 627. 10.3390/f16040627

[B32] Lizcano Salas AF, Duitama J, Restrepo S et al. (2024) Phylogenomic approaches reveal a robust time-scale phylogeny of the Terminal *Fusarium* Clade. IMA Fungus 15: 13. 10.1186/s43008-024-00147-8 PMC11161934 38849861

[B33] McCarthy CGP, Fitzpatrick DA (2019) Pan-genome analyses of model fungal species. Microbial Genomics 5. 10.1099/mgen.0.000243 PMC6421352 30714895

[B34] ME B (1978) The *Diaporthales* in North America with emphasis on *Gnomonia* and its segregates. Mycol Mem 7: 1–232.

[B35] Merényi Z, Krizsán K, Sahu N et al. (2023) Genomes of fungi and relatives reveal delayed loss of ancestral gene families and evolution of key fungal traits. Nature Ecology & Evolution 7: 1221–1231. 10.1038/s41559-023-02095-9 PMC10406608 37349567

[B36] Mondo SJ, Grigoriev IV (2025) A genomic perspective on fungal diversity and evolution. Nature Reviews Microbiology 23: 718–733. 10.1038/s41579-025-01195-6 40588585

[B37] Naqvi SAH, Abbas A, Hasnain A et al. (2025) Advancing fungal phylogenetics: integrating modern sequencing, dark taxa discovery, and machine learning. Archives of Microbiology 207: 192. 10.1007/s00203-025-04392-2 40643763

[B38] O’Donnell K, Kistler HC, Cigelnik E et al. (1998) Multiple evolutionary origins of the fungus causing Panama disease of banana: Concordant evidence from nuclear and mitochondrial gene genealogies. Proceedings of the National Academy of Sciences 95: 2044–2049. 10.1073/pnas.95.5.2044 PMC19243 9482835

[B39] Prjibelski A, Antipov D, Meleshko D et al. (2020) Using SPAdes De Novo Assembler. Current Protocols in Bioinformatics 70: e102. 10.1002/cpbi.102 32559359

[B40] Quaedvlieg W, Binder M, Groenewald JZ et al. (2014) Introducing the consolidated species concept to resolve species in the *Teratosphaeriaceae*. Persoonia – Molecular Phylo­geny and Evolution of Fungi 33: 1–40. 10.3767/003158514X681981 PMC4312929 25737591

[B41] Rossman AY, Farr DF, Castlebury LA (2007) A review of the phylogeny and biology of the *Diaporthales*. Mycoscience 48: 135–144. 10.1007/S10267-007-0347-7

[B42] Ruan Z, Jiao J, Zhao J et al. (2024) Genome sequencing and comparative genomics reveal insights into pathogenicity and evolution of *Fusarium zanthoxyli*, the causal agent of stem canker in prickly ash. BMC Genomics 25: 502. 10.1186/s12864-024-10424-w PMC11110190 38773367

[B43] Sarawad A, Hosagoudar S, Parvatikar P (2025) Pan-genomics: Insight into the functional genome, applications, advancements, and challenges. Current Genomics 26: 2–14. 10.2174/0113892029311541240627111506 PMC11793047 39911277

[B44] Sogonov MV, Castlebury LA, Rossman AY et al. (2007) The type species of *Apiognomonia*, *A. veneta*, with its *Discula anamorph* is distinct from *A. errabunda*. Mycological Research 111: 693–709. 10.1016/j.mycres.2007.03.013 17604146

[B45] Sogonov MV, Castlebury LA, Rossman AY et al. (2008) Leaf-inhabiting genera of the *Gnomoniaceae*, *Diaporthales*. Studies in Mycology 62: 1–77. 10.3114/sim.2008.62.01 PMC2621335 19287541

[B46] Stanke M, Steinkamp R, Waack S et al. (2004) AUGUSTUS: a web server for gene finding in eukaryotes. Nucleic Acids Research 32: W309–W312. 10.1093/nar/gkh379 PMC441517 15215400

[B47] Stengel A, Stanke KM, Quattrone AC et al. (2022) Improving taxonomic delimitation of fungal species in the age of genomics and phenomics. Frontiers in Microbiology 13: 847067. 10.3389/fmicb.2022.847067 PMC8892103 35250961

[B48] Turco S, Mazzaglia A, Drais MI et al. (2023) Hybrid de novo genome assembly and comparative genomics of three different isolates of *Gnomoniopsis castaneae*. Scientific Reports 13: 3356. 10.1038/s41598-023-30496-0 PMC9971261 36849528

[B49] Udayanga D, Miriyagalla SD, Manamgoda DS et al. (2021) Molecular reassessment of diaporthalean fungi associated with strawberry, including the leaf blight fungus, *Paraphomopsis obscurans* gen. et comb. nov. (*Melanconiellaceae*). IMA Fungus 12: 15. 10.1186/s43008-021-00069-9 PMC8218473 34158123

[B50] Visentin I, Gentile S, Valentino D et al. (2012) *Gnomoniopsis castanea* sp. nov. (*Gnomoniaceae*, *Diaporthales*) as the causal agent of nut rot in sweet chestnut. Journal of Plant Pathology 94: 411–419.

[B51] Voglmayr H, Castlebury LA, Jaklitsch WM (2017) *Juglanconis* gen. nov. on *Juglandaceae*, and the new family *Juglanconidaceae (Diaporthales)*. Persoonia – Molecular Phylogeny and Evolution of Fungi 38: 136–155. 10.3767/003158517X694768 PMC5645181 29151630

[B52] Voglmayr H, Rossman AY, Castlebury LA et al. (2012) Multigene phylogeny and taxonomy of the genus *Melanconiella (Diaporthales)*. Fungal Diversity 57: 1–44. 10.1007/s13225-012-0175-8

[B53] Walker DM (2012) Taxonomy, systematics, ecology, and evolutionary biology of the *Gnomoniaceae (Diaporthales)*, with emphasis on *Gnomoniopsis* and *Ophiognomonia*. Rutgers The State University of New Jersey, School of Graduate Studies.

[B54] Walker DM, Castlebury LA, Rossman AY et al. (2010) Systematics of genus *Gnomo­niopsis* (*Gnomoniaceae*, *Diaporthales*) based on a three gene phylogeny, host associations and morphology. Mycologia 102: 1479–1496. 10.3852/10-002 20943552

[B55] Wang S, Zhang Z, Liu R et al. (2022) Morphological and phylogenetic analyses reveal four new species of *Gnomoniopsis* (*Gnomoniaceae*, *Diaporthales*) from China. Journal of Fungi 8: 770. 10.3390/jof8080770 PMC9331987 35893138

[B56] Wang S, Liu X, Xiong C et al. (2023a) ASF1 regulates asexual and sexual reproduction in *Stemphylium eturmiunum* by DJ-1 stimulation of the PI3K/AKT signaling pathway. Fungal Diversity 123: 159–176. 10.1007/s13225-023-00528-1

[B57] Wang Y, Chang Y, Ortañez J et al. (2023b) Divergent evolution of early terrestrial fungi reveals the evolution of *Mucormycosis* pathogenicity factors. Ma L-J (Ed.). Genome Biology and Evolution 15: evad046. 10.1093/gbe/evad046 PMC10079182 36930540

[B58] White TJ, Bruns T, Lee S et al. (1990) Amplification and direct sequencing of fungal ribosomal RNA genes for phylogenetics. In: PCR Protocols. Elsevier, 315–322. 10.1016/B978-0-12-372180-8.50042-1

[B59] Wisecaver JH, Slot JC, Rokas A (2014) The Evolution of Fungal Metabolic Pathways. Stajich JE (Ed.). PLOS Genetics 10: e1004816. 10.1371/journal.pgen.1004816 PMC4256263 25474404

[B60] Xu J (2020) Fungal species concepts in the genomics era. Genome 63: 459–468. 10.1139/gen-2020-0022 32531173

[B61] Yang Q, Jiang N, Tian C-M (2020) Tree inhabiting gnomoniaceous species from China, with *Cryphogonomonia* gen. nov. proposed. MycoKeys 69: 71–89. 10.3897/mycokeys.69.54012 PMC7367894 32733150

[B62] Zhang M-Y, Zhang Z-X, Wang Y-L et al. (2025a) Molecular and morphological characterization of four new *Phyllosticta* species (*Botryosphaeriales*, *Phyllostictaceae*): Genomic insights into evolutionary dynamics and metabolic adaptation. IMA Fungus 16: e168055. 10.3897/imafungus.16.168055 PMC12559956 41164718

[B63] Zhang Z-X, Shang Y-X, Liu Q-Y et al. (2025b) Deciphering the evolutionary and taxonomic complexity of *Diaporthales* (*Sordariomycetes*, *Ascomycota*) through integrated phylo­genomic and divergence time estimation. Fungal Diversity 132: 1–125. 10.1007/s13225-025-00551-4

