# Supplementary material for: Molecular and morphological characterization of nine new Gnomoniopsis species (Diaporthales, Gnomoniaceae): Genomic insights into evolution and phylogeny
Source: IMA Fungus. 2026 Oct 23;17:e208364. doi: 10.3897/imafungus.17.208364 (PMC13723015; doi:10.3897/imafungus.17.208364)
Supplement: Supplementary file 2 [file imafungus-17-e208364-s002.zip › Supplementary material 2/Supplementary figure 2 Gnomoniopsis tef1 ML.pdf]

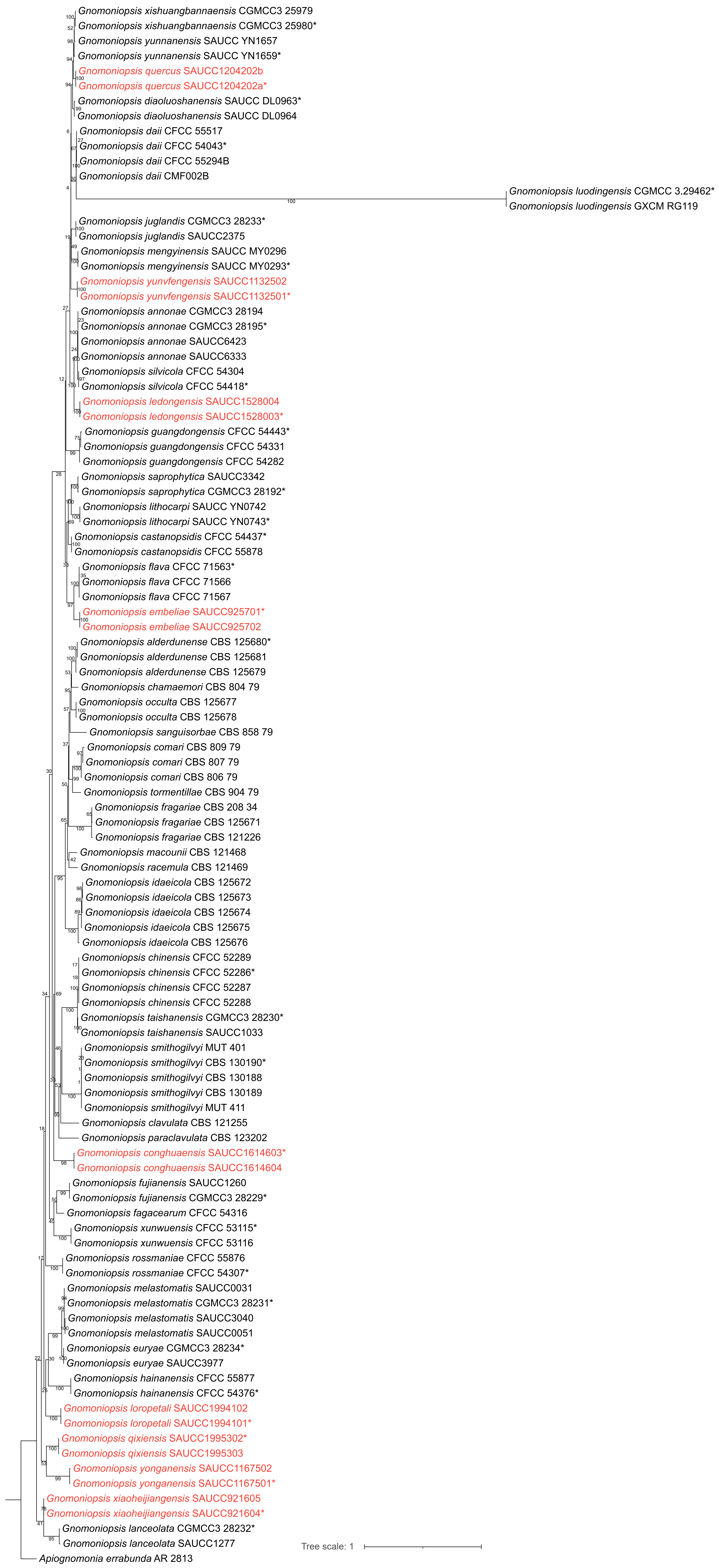

Gnomoniopsis xishuangbannaensis CGMCC3 25979
Gnomoniopsis xishuangbannaensis CGMCC3 25980*
Gnomoniopsis yunnanensis SAUCC YN1657
Gnomoniopsis yunnanensis SAUCC YN1659*
Gnomoniopsis quercus SAUCC1204202b
Gnomoniopsis quercus SAUCC1204202a*
Gnomoniopsis diaoluoshanensis SAUCC DL0963*
Gnomoniopsis diaoluoshanensis SAUCC DL0964
Gnomoniopsis daii CFCC 55517
Gnomoniopsis daii CFCC 54043*
Gnomoniopsis daii CFCC 55294B
Gnomoniopsis daii CMF002B
Gnomoniopsis luodingensis CGMCC 3.29462*
Gnomoniopsis luodingensis GXCM RG119
Gnomoniopsis juglandis CGMCC3 28233*
Gnomoniopsis juglandis SAUCC2375
Gnomoniopsis mengyinensis SAUCC MY0296
Gnomoniopsis mengyinensis SAUCC MY0293*
Gnomoniopsis yunvfengensis SAUCC1132502
Gnomoniopsis yunvfengensis SAUCC1132501*
Gnomoniopsis annonae CGMCC3 28194
Gnomoniopsis annonae CGMCC3 28195*
Gnomoniopsis annonae SAUCC6423
Gnomoniopsis annonae SAUCC6333
Gnomoniopsis silvicola CFCC 54304
Gnomoniopsis silvicola CFCC 54418*
Gnomoniopsis ledongensis SAUCC1528004
Gnomoniopsis ledongensis SAUCC1528003*
Gnomoniopsis guangdongensis CFCC 54443*
Gnomoniopsis guangdongensis CFCC 54331
Gnomoniopsis guangdongensis CFCC 54282
Gnomoniopsis saprophytica SAUCC3342
Gnomoniopsis saprophytica CGMCC3 28192*
Gnomoniopsis lithocarpi SAUCC YN0742
Gnomoniopsis lithocarpi SAUCC YN0743*
Gnomoniopsis castanopsidis CFCC 54437*
Gnomoniopsis castanopsidis CFCC 55878
Gnomoniopsis flava CFCC 71563*
Gnomoniopsis flava CFCC 71566
Gnomoniopsis flava CFCC 71567
Gnomoniopsis embeliae SAUCC925701*
Gnomoniopsis embeliae SAUCC925702
Gnomoniopsis alderdunense CBS 125680*
Gnomoniopsis alderdunense CBS 125681
Gnomoniopsis alderdunense CBS 125679
Gnomoniopsis chamaemori CBS 804 79
Gnomoniopsis occulta CBS 125677
Gnomoniopsis occulta CBS 125678
Gnomoniopsis sanguisorbae CBS 858 79
Gnomoniopsis comari CBS 809 79
Gnomoniopsis comari CBS 807 79
Gnomoniopsis comari CBS 806 79
Gnomoniopsis tormentillae CBS 904 79
Gnomoniopsis fragariae CBS 208 34
Gnomoniopsis fragariae CBS 125671
Gnomoniopsis fragariae CBS 121226
Gnomoniopsis macounii CBS 121468
Gnomoniopsis racemula CBS 121469
Gnomoniopsis idaeicola CBS 125672
Gnomoniopsis idaeicola CBS 125673
Gnomoniopsis idaeicola CBS 125674
Gnomoniopsis idaeicola CBS 125675
Gnomoniopsis idaeicola CBS 125676
Gnomoniopsis chinensis CFCC 52289
Gnomoniopsis chinensis CFCC 52286*
Gnomoniopsis chinensis CFCC 52287
Gnomoniopsis chinensis CFCC 52288
Gnomoniopsis taishanensis CGMCC3 28230*
Gnomoniopsis taishanensis SAUCC1033
Gnomoniopsis smithogilvyi MUT 401
Gnomoniopsis smithogilvyi CBS 130190*
Gnomoniopsis smithogilvyi CBS 130188
Gnomoniopsis smithogilvyi CBS 130189
Gnomoniopsis smithogilvyi MUT 411
Gnomoniopsis clavulata CBS 121255
Gnomoniopsis paraclavulata CBS 123202
Gnomoniopsis conghuaensis SAUCC1614603*
Gnomoniopsis conghuaensis SAUCC1614604
Gnomoniopsis fujianensis SAUCC1260
Gnomoniopsis fujianensis CGMCC3 28229*
Gnomoniopsis fagacearum CFCC 54316
Gnomoniopsis xunwuensis CFCC 53115*
Gnomoniopsis xunwuensis CFCC 53116
Gnomoniopsis rossmaniae CFCC 55876
Gnomoniopsis rossmaniae CFCC 54307*
Gnomoniopsis melastomatis SAUCC0031
Gnomoniopsis melastomatis CGMCC3 28231*
Gnomoniopsis melastomatis SAUCC3040
Gnomoniopsis melastomatis SAUCC0051
Gnomoniopsis euryae CGMCC3 28234*
Gnomoniopsis euryae SAUCC3977
Gnomoniopsis hainanensis CFCC 55877
Gnomoniopsis hainanensis CFCC 54376*
Gnomoniopsis loropetali SAUCC1994102
Gnomoniopsis loropetali SAUCC1994101*
Gnomoniopsis qixiensis SAUCC1995302*
Gnomoniopsis qixiensis SAUCC1995303
Gnomoniopsis yonganensis SAUCC1167502
Gnomoniopsis yonganensis SAUCC1167501*
Gnomoniopsis xiaoheijiangensis SAUCC921605
Gnomoniopsis xiaoheijiangensis SAUCC921604*
Gnomoniopsis lanceolata CGMCC3 28232*
Gnomoniopsis lanceolata SAUCC1277
Apiognomonia errabunda AR 2813
Tree scale: 1

Supplementary figure S2: Single-locus analyses of *Gnomoniopsis* were based on *tef1* sequence alignments. *Apiognomonia errabunda* was used as the outgroup. Maximum likelihood bootstrap support values are shown above the nodes. Strains isolated in the present study are highlighted in red.
