# Supplementary material for: Molecular and morphological characterization of nine new Gnomoniopsis species (Diaporthales, Gnomoniaceae): Genomic insights into evolution and phylogeny
Source: IMA Fungus. 2026 Oct 23;17:e208364. doi: 10.3897/imafungus.17.208364 (PMC13723015; doi:10.3897/imafungus.17.208364)
Supplement: Supplementary file 2 [file imafungus-17-e208364-s002.zip › Supplementary material 2/Supplementary figure 3 Gnomoniopsis tub2 ML.pdf]

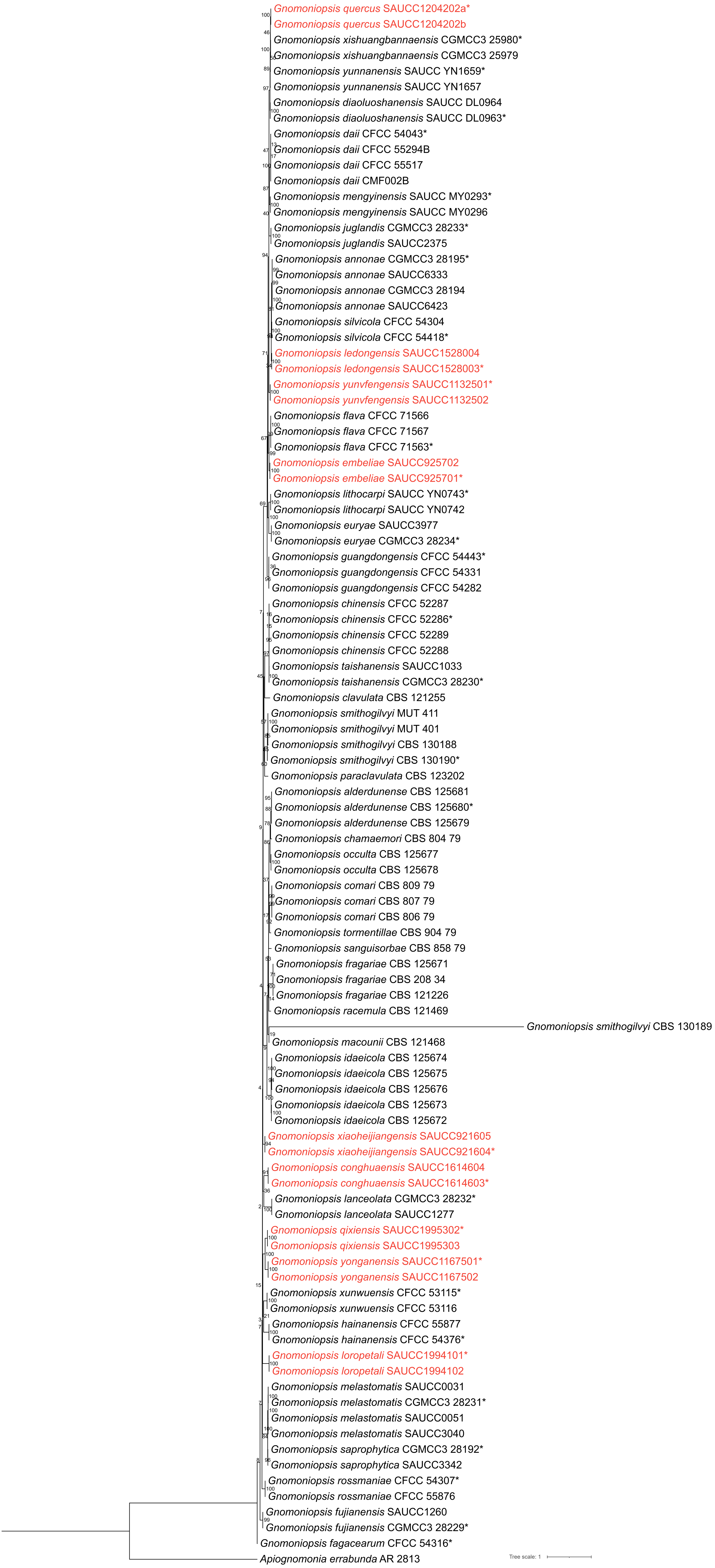

Supplementary figure S3: Single-locus analyses of *Gnomoniopsis* were based on *tub2* sequence alignments. *Apiognomonia errabunda* was used as the outgroup. Maximum likelihood bootstrap support values are shown above the nodes. Strains isolated in the present study are highlighted in red.
