# Supplementary material for: Molecular and morphological characterization of nine new Gnomoniopsis species (Diaporthales, Gnomoniaceae): Genomic insights into evolution and phylogeny
Source: IMA Fungus. 2026 Oct 23;17:e208364. doi: 10.3897/imafungus.17.208364 (PMC13723015; doi:10.3897/imafungus.17.208364)
Supplement: Supplementary file 3 [file imafungus-17-e208364-s003.zip › Supplementary material 3.docx]

**Fig. S1** PHI (Pairwise Homoplasy Index) tests for closely related species of the genus Gnomoniopsis were performed in SplitsTree using the LogDet transformation and the NeighborNet algorithm. Our isolates are marked in red text.

**Fig. S2** PHI (Pairwise Homoplasy Index) tests for closely related species of the genus Gnomoniopsis were performed in SplitsTree using the LogDet transformation and the NeighborNet algorithm. Our isolates are marked in red text.

**Fig. S3** PHI (Pairwise Homoplasy Index) tests for closely related species of the genus Gnomoniopsis were performed in SplitsTree using the LogDet transformation and the NeighborNet algorithm. Our isolates are marked in red text.

**Fig. S4** PHI (Pairwise Homoplasy Index) tests for closely related species of the genus Gnomoniopsis were performed in SplitsTree using the LogDet transformation and the NeighborNet algorithm. Our isolates are marked in red text.

**Fig. S5** PHI (Pairwise Homoplasy Index) tests for closely related species of the genus Gnomoniopsis were performed in SplitsTree using the LogDet transformation and the NeighborNet algorithm. Our isolates are marked in red text.

**Fig. S6** PHI (Pairwise Homoplasy Index) tests for closely related species of the genus Gnomoniopsis were performed in SplitsTree using the LogDet transformation and the NeighborNet algorithm. Our isolates are marked in red text.
