# Supplementary material for: Molecular and morphological characterization of nine new Gnomoniopsis species (Diaporthales, Gnomoniaceae): Genomic insights into evolution and phylogeny
Source: IMA Fungus. 2026 Oct 23;17:e208364. doi: 10.3897/imafungus.17.208364 (PMC13723015; doi:10.3897/imafungus.17.208364)
Supplement: Supplementary file 4 [file imafungus-17-e208364-s004.zip › Supplementary material 4.docx]

Tab. S1 Analysis results of secondary metabolic biosynthesis gene clusters of *Gnomoniopsis xiaoheijiangensis*

| Region | Type | From | To | Similarity Confidence | Most similar known cluster |
| --- | --- | --- | --- | --- | --- |
| [Region 1.1](https://fungismash.secondarymetabolites.org/upload/fungi-d0fdb138-d28e-4127-b514-9fa292b383a2/index.html#r1c1) | [NRPS-like](https://docs.antismash.secondarymetabolites.org/glossary/#nrps-like) | 1681 | 65064 |  |  |
| [Region 1.2](https://fungismash.secondarymetabolites.org/upload/fungi-d0fdb138-d28e-4127-b514-9fa292b383a2/index.html#r1c2) | T1PKS,terpene | 986073 | 1054403 |  |  |
| [Region 1.3](https://fungismash.secondarymetabolites.org/upload/fungi-d0fdb138-d28e-4127-b514-9fa292b383a2/index.html#r1c3) | [T1PKS](https://docs.antismash.secondarymetabolites.org/glossary/#t1pks) | 1239638 | 1306503 |  |  |
| [Region 1.4](https://fungismash.secondarymetabolites.org/upload/fungi-d0fdb138-d28e-4127-b514-9fa292b383a2/index.html#r1c4) | [NRPS-like](https://docs.antismash.secondarymetabolites.org/glossary/#nrps-like) | 2346792 | 2410250 |  |  |
| [Region 2.1](https://fungismash.secondarymetabolites.org/upload/fungi-d0fdb138-d28e-4127-b514-9fa292b383a2/index.html#r2c1) | [T1PKS](https://docs.antismash.secondarymetabolites.org/glossary/#t1pks) | 90339 | 158801 |  |  |
| [Region 2.2](https://fungismash.secondarymetabolites.org/upload/fungi-d0fdb138-d28e-4127-b514-9fa292b383a2/index.html#r2c2) | [NRPS-like](https://docs.antismash.secondarymetabolites.org/glossary/#nrps-like) | 1460154 | 1524359 |  |  |
| [Region 3.1](https://fungismash.secondarymetabolites.org/upload/fungi-d0fdb138-d28e-4127-b514-9fa292b383a2/index.html#r3c1) | NRPS,T1PKS | 1186333 | 1259295 |  |  |
| [Region 3.2](https://fungismash.secondarymetabolites.org/upload/fungi-d0fdb138-d28e-4127-b514-9fa292b383a2/index.html#r3c2) | [isocyanide](https://docs.antismash.secondarymetabolites.org/glossary/#isocyanide) | 1343915 | 1406150 |  |  |
| [Region 3.3](https://fungismash.secondarymetabolites.org/upload/fungi-d0fdb138-d28e-4127-b514-9fa292b383a2/index.html#r3c3) | [T1PKS](https://docs.antismash.secondarymetabolites.org/glossary/#t1pks) | 1687077 | 1800802 |  |  |
| [Region 4.1](https://fungismash.secondarymetabolites.org/upload/fungi-d0fdb138-d28e-4127-b514-9fa292b383a2/index.html#r4c1) | NRPS-like,terpene | 1804753 | 1850479 |  |  |
| [Region 5.1](https://fungismash.secondarymetabolites.org/upload/fungi-d0fdb138-d28e-4127-b514-9fa292b383a2/index.html#r5c1) | [terpene](https://docs.antismash.secondarymetabolites.org/glossary/#terpene) | 49905 | 80489 |  |  |
| [Region 5.2](https://fungismash.secondarymetabolites.org/upload/fungi-d0fdb138-d28e-4127-b514-9fa292b383a2/index.html#r5c2) | [NRPS](https://docs.antismash.secondarymetabolites.org/glossary/#nrps) | 194568 | 259676 |  |  |
| [Region 5.3](https://fungismash.secondarymetabolites.org/upload/fungi-d0fdb138-d28e-4127-b514-9fa292b383a2/index.html#r5c3) | [NRPS](https://docs.antismash.secondarymetabolites.org/glossary/#nrps) | 786189 | 850036 |  |  |
| [Region 5.4](https://fungismash.secondarymetabolites.org/upload/fungi-d0fdb138-d28e-4127-b514-9fa292b383a2/index.html#r5c4) | [indole](https://docs.antismash.secondarymetabolites.org/glossary/#indole) | 1275301 | 1305843 |  |  |
| [Region 5.5](https://fungismash.secondarymetabolites.org/upload/fungi-d0fdb138-d28e-4127-b514-9fa292b383a2/index.html#r5c5) | [NRPS](https://docs.antismash.secondarymetabolites.org/glossary/#nrps) | 1368586 | 1439178 |  |  |
| [Region 5.6](https://fungismash.secondarymetabolites.org/upload/fungi-d0fdb138-d28e-4127-b514-9fa292b383a2/index.html#r5c6) | [NRPS-like](https://docs.antismash.secondarymetabolites.org/glossary/#nrps-like) | 1614396 | 1677948 |  |  |
| [Region 5.7](https://fungismash.secondarymetabolites.org/upload/fungi-d0fdb138-d28e-4127-b514-9fa292b383a2/index.html#r5c7) | [T1PKS](https://docs.antismash.secondarymetabolites.org/glossary/#t1pks) | 1750640 | 1813561 | Medium | [hypothemycin](https://mibig.secondarymetabolites.org/go/BGC0000076.3) |
| [Region 6.1](https://fungismash.secondarymetabolites.org/upload/fungi-d0fdb138-d28e-4127-b514-9fa292b383a2/index.html#r6c1) | [NI-siderophore](https://docs.antismash.secondarymetabolites.org/glossary/#ni-siderophore) | 241334 | 285491 |  |  |
| [Region 6.2](https://fungismash.secondarymetabolites.org/upload/fungi-d0fdb138-d28e-4127-b514-9fa292b383a2/index.html#r6c2) | terpene,T1PKS | 421197 | 522616 | Medium | [copalyl diphosphate](https://mibig.secondarymetabolites.org/go/BGC0000688.4) |
| [Region 7.1](https://fungismash.secondarymetabolites.org/upload/fungi-d0fdb138-d28e-4127-b514-9fa292b383a2/index.html#r7c1) | T1PKS,NRPS | 865787 | 988190 | Low | [leucinostatin A/leucinostatin B](https://mibig.secondarymetabolites.org/go/BGC0001358.4) |
| [Region 7.2](https://fungismash.secondarymetabolites.org/upload/fungi-d0fdb138-d28e-4127-b514-9fa292b383a2/index.html#r7c2) | [terpene](https://docs.antismash.secondarymetabolites.org/glossary/#terpene) | 1161373 | 1192453 |  |  |
| [Region 7.3](https://fungismash.secondarymetabolites.org/upload/fungi-d0fdb138-d28e-4127-b514-9fa292b383a2/index.html#r7c3) | [terpene](https://docs.antismash.secondarymetabolites.org/glossary/#terpene) | 1276038 | 1308495 | High | [clavaric acid](https://mibig.secondarymetabolites.org/go/BGC0001248.3) |
| [Region 7.4](https://fungismash.secondarymetabolites.org/upload/fungi-d0fdb138-d28e-4127-b514-9fa292b383a2/index.html#r7c4) | T1PKS,NRPS-like | 1339393 | 1452866 |  |  |
| [Region 8.1](https://fungismash.secondarymetabolites.org/upload/fungi-d0fdb138-d28e-4127-b514-9fa292b383a2/index.html#r8c1) | [indole](https://docs.antismash.secondarymetabolites.org/glossary/#indole) | 450286 | 481846 |  |  |
| [Region 8.2](https://fungismash.secondarymetabolites.org/upload/fungi-d0fdb138-d28e-4127-b514-9fa292b383a2/index.html#r8c2) | [terpene](https://docs.antismash.secondarymetabolites.org/glossary/#terpene) | 531364 | 562540 |  |  |
| [Region 9.1](https://fungismash.secondarymetabolites.org/upload/fungi-d0fdb138-d28e-4127-b514-9fa292b383a2/index.html#r9c1) | [terpene-precursor](https://docs.antismash.secondarymetabolites.org/glossary/#terpene-precursor) | 112178 | 143379 |  |  |
| [Region 9.2](https://fungismash.secondarymetabolites.org/upload/fungi-d0fdb138-d28e-4127-b514-9fa292b383a2/index.html#r9c2) | [NRPS](https://docs.antismash.secondarymetabolites.org/glossary/#nrps) | 264120 | 328877 | Low | [aspirochlorine](https://mibig.secondarymetabolites.org/go/BGC0001123.5) |
| [Region 9.3](https://fungismash.secondarymetabolites.org/upload/fungi-d0fdb138-d28e-4127-b514-9fa292b383a2/index.html#r9c3) | [NRPS-like](https://docs.antismash.secondarymetabolites.org/glossary/#nrps-like) | 976923 | 1040506 |  |  |
| [Region 10.1](https://fungismash.secondarymetabolites.org/upload/fungi-d0fdb138-d28e-4127-b514-9fa292b383a2/index.html#r10c1) | [terpene](https://docs.antismash.secondarymetabolites.org/glossary/#terpene) | 577261 | 608485 |  |  |
| [Region 10.2](https://fungismash.secondarymetabolites.org/upload/fungi-d0fdb138-d28e-4127-b514-9fa292b383a2/index.html#r10c2) | [T1PKS](https://docs.antismash.secondarymetabolites.org/glossary/#t1pks) | 848660 | 916906 |  |  |
| [Region 11.1](https://fungismash.secondarymetabolites.org/upload/fungi-d0fdb138-d28e-4127-b514-9fa292b383a2/index.html#r11c1) | [terpene](https://docs.antismash.secondarymetabolites.org/glossary/#terpene) | 432785 | 464948 |  |  |
| [Region 11.2](https://fungismash.secondarymetabolites.org/upload/fungi-d0fdb138-d28e-4127-b514-9fa292b383a2/index.html#r11c2) | [NRPS](https://docs.antismash.secondarymetabolites.org/glossary/#nrps) | 748428 | 812296 |  |  |
| [Region 12.1](https://fungismash.secondarymetabolites.org/upload/fungi-d0fdb138-d28e-4127-b514-9fa292b383a2/index.html#r12c1) | [T1PKS](https://docs.antismash.secondarymetabolites.org/glossary/#t1pks) | 341505 | 411956 |  |  |
| [Region 12.2](https://fungismash.secondarymetabolites.org/upload/fungi-d0fdb138-d28e-4127-b514-9fa292b383a2/index.html#r12c2) | [terpene](https://docs.antismash.secondarymetabolites.org/glossary/#terpene) | 415462 | 446903 |  |  |
| [Region 12.3](https://fungismash.secondarymetabolites.org/upload/fungi-d0fdb138-d28e-4127-b514-9fa292b383a2/index.html#r12c3) | [terpene](https://docs.antismash.secondarymetabolites.org/glossary/#terpene) | 970677 | 1003597 |  |  |
| [Region 14.1](https://fungismash.secondarymetabolites.org/upload/fungi-d0fdb138-d28e-4127-b514-9fa292b383a2/index.html#r14c1) | [terpene-precursor](https://docs.antismash.secondarymetabolites.org/glossary/#terpene-precursor) | 911939 | 943246 |  |  |
| [Region 16.1](https://fungismash.secondarymetabolites.org/upload/fungi-d0fdb138-d28e-4127-b514-9fa292b383a2/index.html#r16c1) | NRPS-like,T1PKS | 193022 | 258275 |  |  |
| [Region 16.2](https://fungismash.secondarymetabolites.org/upload/fungi-d0fdb138-d28e-4127-b514-9fa292b383a2/index.html#r16c2) | NRPS,T1PKS | 425310 | 496407 | Low | [gregatin A](https://mibig.secondarymetabolites.org/go/BGC0002185.2) |
| [Region 16.3](https://fungismash.secondarymetabolites.org/upload/fungi-d0fdb138-d28e-4127-b514-9fa292b383a2/index.html#r16c3) | [T1PKS](https://docs.antismash.secondarymetabolites.org/glossary/#t1pks) | 637011 | 725672 | Low | [tricholignan A](https://mibig.secondarymetabolites.org/go/BGC0001854.4) |
| [Region 16.4](https://fungismash.secondarymetabolites.org/upload/fungi-d0fdb138-d28e-4127-b514-9fa292b383a2/index.html#r16c4) | NRPS-like,T1PKS | 746577 | 845118 |  |  |

Tab. S1 Analysis results of secondary metabolic biosynthesis gene clusters of *Gnomoniopsis xiaoheijiangensis* (Continued)

| **Region** | **Type** | **From** | **To** | **Similarity Confidence** | **Most similar known cluster** |
| --- | --- | --- | --- | --- | --- |
| [Region 16.5](https://fungismash.secondarymetabolites.org/upload/fungi-d0fdb138-d28e-4127-b514-9fa292b383a2/index.html#r16c5) | [NRPS-like](https://docs.antismash.secondarymetabolites.org/glossary/#nrps-like) | 914610 | 963181 |  |  |
| [Region 17.1](https://fungismash.secondarymetabolites.org/upload/fungi-d0fdb138-d28e-4127-b514-9fa292b383a2/index.html#r17c1) | [T1PKS](https://docs.antismash.secondarymetabolites.org/glossary/#t1pks) | 603127 | 671130 | High | [alternapyrone](https://mibig.secondarymetabolites.org/go/BGC0000012.3) |
| [Region 17.2](https://fungismash.secondarymetabolites.org/upload/fungi-d0fdb138-d28e-4127-b514-9fa292b383a2/index.html#r17c2) | [NRPS](https://docs.antismash.secondarymetabolites.org/glossary/#nrps) | 756173 | 830375 |  |  |
| [Region 18.1](https://fungismash.secondarymetabolites.org/upload/fungi-d0fdb138-d28e-4127-b514-9fa292b383a2/index.html#r18c1) | NRPS-like,NRPS,T1PKS | 2212 | 93675 | Low | [cytochalasin E/cytochalasin K](https://mibig.secondarymetabolites.org/go/BGC0000983.5) |
| [Region 20.1](https://fungismash.secondarymetabolites.org/upload/fungi-d0fdb138-d28e-4127-b514-9fa292b383a2/index.html#r20c1) | NRPS,terpene,indole | 337964 | 414743 |  |  |
| [Region 20.2](https://fungismash.secondarymetabolites.org/upload/fungi-d0fdb138-d28e-4127-b514-9fa292b383a2/index.html#r20c2) | [T1PKS](https://docs.antismash.secondarymetabolites.org/glossary/#t1pks) | 449078 | 517306 |  |  |
| [Region 21.1](https://fungismash.secondarymetabolites.org/upload/fungi-d0fdb138-d28e-4127-b514-9fa292b383a2/index.html#r21c1) | [T1PKS](https://docs.antismash.secondarymetabolites.org/glossary/#t1pks) | 1 | 75376 | Low | [zearalenone](https://mibig.secondarymetabolites.org/go/BGC0001057.4) |
| [Region 21.2](https://fungismash.secondarymetabolites.org/upload/fungi-d0fdb138-d28e-4127-b514-9fa292b383a2/index.html#r21c2) | [T1PKS](https://docs.antismash.secondarymetabolites.org/glossary/#t1pks) | 95137 | 183398 |  |  |
| [Region 21.3](https://fungismash.secondarymetabolites.org/upload/fungi-d0fdb138-d28e-4127-b514-9fa292b383a2/index.html#r21c3) | [NRPS](https://docs.antismash.secondarymetabolites.org/glossary/#nrps) | 231455 | 307141 |  |  |
| [Region 23.1](https://fungismash.secondarymetabolites.org/upload/fungi-d0fdb138-d28e-4127-b514-9fa292b383a2/index.html#r23c1) | [other](https://docs.antismash.secondarymetabolites.org/glossary/#other) | 356959 | 417873 |  |  |
| [Region 23.2](https://fungismash.secondarymetabolites.org/upload/fungi-d0fdb138-d28e-4127-b514-9fa292b383a2/index.html#r23c2) | [terpene](https://docs.antismash.secondarymetabolites.org/glossary/#terpene) | 599426 | 630961 |  |  |
| [Region 24.1](https://fungismash.secondarymetabolites.org/upload/fungi-d0fdb138-d28e-4127-b514-9fa292b383a2/index.html#r24c1) | NRPS-like,T1PKS | 471137 | 544056 |  |  |
| [Region 25.1](https://fungismash.secondarymetabolites.org/upload/fungi-d0fdb138-d28e-4127-b514-9fa292b383a2/index.html#r25c1) | NRP-metallophore,NRPS | 1 | 55861 |  |  |
| [Region 25.2](https://fungismash.secondarymetabolites.org/upload/fungi-d0fdb138-d28e-4127-b514-9fa292b383a2/index.html#r25c2) | [NRPS](https://docs.antismash.secondarymetabolites.org/glossary/#nrps) | 103572 | 171524 |  |  |
| [Region 25.3](https://fungismash.secondarymetabolites.org/upload/fungi-d0fdb138-d28e-4127-b514-9fa292b383a2/index.html#r25c3) | [terpene](https://docs.antismash.secondarymetabolites.org/glossary/#terpene) | 217533 | 249140 | Low | [squalestatin S1](https://mibig.secondarymetabolites.org/go/BGC0001839.3) |
| [Region 27.1](https://fungismash.secondarymetabolites.org/upload/fungi-d0fdb138-d28e-4127-b514-9fa292b383a2/index.html#r27c1) | [NRPS](https://docs.antismash.secondarymetabolites.org/glossary/#nrps) | 77204 | 156997 |  |  |
| [Region 30.1](https://fungismash.secondarymetabolites.org/upload/fungi-d0fdb138-d28e-4127-b514-9fa292b383a2/index.html#r30c1) | [T1PKS](https://docs.antismash.secondarymetabolites.org/glossary/#t1pks) | 344004 | 411715 | Low | [depudecin](https://mibig.secondarymetabolites.org/go/BGC0000046.4) |
| [Region 32.1](https://fungismash.secondarymetabolites.org/upload/fungi-d0fdb138-d28e-4127-b514-9fa292b383a2/index.html#r32c1) | [NRPS-like](https://docs.antismash.secondarymetabolites.org/glossary/#nrps-like) | 57094 | 121060 | High | [choline](https://mibig.secondarymetabolites.org/go/BGC0002276.2) |
| [Region 33.1](https://fungismash.secondarymetabolites.org/upload/fungi-d0fdb138-d28e-4127-b514-9fa292b383a2/index.html#r33c1) | [T1PKS](https://docs.antismash.secondarymetabolites.org/glossary/#t1pks) | 1 | 41255 |  |  |
| [Region 34.1](https://fungismash.secondarymetabolites.org/upload/fungi-d0fdb138-d28e-4127-b514-9fa292b383a2/index.html#r34c1) | [T1PKS](https://docs.antismash.secondarymetabolites.org/glossary/#t1pks) | 59151 | 126606 |  |  |
| [Region 35.1](https://fungismash.secondarymetabolites.org/upload/fungi-d0fdb138-d28e-4127-b514-9fa292b383a2/index.html#r35c1) | [T1PKS](https://docs.antismash.secondarymetabolites.org/glossary/#t1pks) | 56265 | 122912 | Medium | [scytalone/T3HN](https://mibig.secondarymetabolites.org/go/BGC0002161.2) |
| [Region 37.1](https://fungismash.secondarymetabolites.org/upload/fungi-d0fdb138-d28e-4127-b514-9fa292b383a2/index.html#r37c1) | [indole](https://docs.antismash.secondarymetabolites.org/glossary/#indole) | 48765 | 80099 |  |  |
| [Region 42.1](https://fungismash.secondarymetabolites.org/upload/fungi-d0fdb138-d28e-4127-b514-9fa292b383a2/index.html#r42c1) | [T3PKS](https://docs.antismash.secondarymetabolites.org/glossary/#t3pks) | 92730 | 154277 |  |  |
| [Region 43.1](https://fungismash.secondarymetabolites.org/upload/fungi-d0fdb138-d28e-4127-b514-9fa292b383a2/index.html#r43c1) | [NRPS-like](https://docs.antismash.secondarymetabolites.org/glossary/#nrps-like) | 113279 | 176911 |  |  |
| [Region 44.1](https://fungismash.secondarymetabolites.org/upload/fungi-d0fdb138-d28e-4127-b514-9fa292b383a2/index.html#r44c1) | NRPS-like,T1PKS | 1 | 73700 | Low | [monacolin K](https://mibig.secondarymetabolites.org/go/BGC0000098.3) |
| [Region 47.1](https://fungismash.secondarymetabolites.org/upload/fungi-d0fdb138-d28e-4127-b514-9fa292b383a2/index.html#r47c1) | NRPS-like,T1PKS | 1 | 80686 |  |  |
| [Region 51.1](https://fungismash.secondarymetabolites.org/upload/fungi-d0fdb138-d28e-4127-b514-9fa292b383a2/index.html#r51c1) | NRPS,T1PKS | 5651 | 88809 |  |  |
| [Region 58.1](https://fungismash.secondarymetabolites.org/upload/fungi-d0fdb138-d28e-4127-b514-9fa292b383a2/index.html#r58c1) | [terpene](https://docs.antismash.secondarymetabolites.org/glossary/#terpene) | 821 | 32375 |  |  |
| [Region 67.1](https://fungismash.secondarymetabolites.org/upload/fungi-d0fdb138-d28e-4127-b514-9fa292b383a2/index.html#r67c1) | [T1PKS](https://docs.antismash.secondarymetabolites.org/glossary/#t1pks) | 1 | 34987 |  |  |
| [Region 68.1](https://fungismash.secondarymetabolites.org/upload/fungi-d0fdb138-d28e-4127-b514-9fa292b383a2/index.html#r68c1) | NRPS-like,T1PKS | 1 | 32270 |  |  |

Tab. S2 Analysis results of secondary metabolic biosynthesis gene clusters of *Gnomoniopsis embeliae*

| **Region** | **Type** | **From** | **To** | **Similarity Confidence** | **Most similar known cluster** |
| --- | --- | --- | --- | --- | --- |
| [Region 1.1](https://fungismash.secondarymetabolites.org/upload/fungi-d7f1bbd7-b6b0-41dd-9d7d-008171430e20/index.html#r1c1) | [indole](https://docs.antismash.secondarymetabolites.org/glossary/#indole) | 853430 | 884997 |  |  |
| [Region 1.2](https://fungismash.secondarymetabolites.org/upload/fungi-d7f1bbd7-b6b0-41dd-9d7d-008171430e20/index.html#r1c2) | [NRPS](https://docs.antismash.secondarymetabolites.org/glossary/#nrps) | 3052643 | 3117884 |  |  |
| [Region 2.1](https://fungismash.secondarymetabolites.org/upload/fungi-d7f1bbd7-b6b0-41dd-9d7d-008171430e20/index.html#r2c1) | [NI-siderophore](https://docs.antismash.secondarymetabolites.org/glossary/#ni-siderophore) | 1162064 | 1206215 |  |  |
| [Region 2.2](https://fungismash.secondarymetabolites.org/upload/fungi-d7f1bbd7-b6b0-41dd-9d7d-008171430e20/index.html#r2c2) | [terpene](https://docs.antismash.secondarymetabolites.org/glossary/#terpene) | 3318895 | 3344396 |  |  |
| [Region 4.1](https://fungismash.secondarymetabolites.org/upload/fungi-d7f1bbd7-b6b0-41dd-9d7d-008171430e20/index.html#r4c1) | [terpene](https://docs.antismash.secondarymetabolites.org/glossary/#terpene) | 68833 | 100463 | Low | [squalestatin S1](https://mibig.secondarymetabolites.org/go/BGC0001839.3) |
| [Region 4.2](https://fungismash.secondarymetabolites.org/upload/fungi-d7f1bbd7-b6b0-41dd-9d7d-008171430e20/index.html#r4c2) | [NRPS-like](https://docs.antismash.secondarymetabolites.org/glossary/#nrps-like) | 390201 | 456684 |  |  |
| [Region 4.3](https://fungismash.secondarymetabolites.org/upload/fungi-d7f1bbd7-b6b0-41dd-9d7d-008171430e20/index.html#r4c3) | [phosphonate](https://docs.antismash.secondarymetabolites.org/glossary/#phosphonate) | 1991461 | 2007939 |  |  |
| [Region 5.1](https://fungismash.secondarymetabolites.org/upload/fungi-d7f1bbd7-b6b0-41dd-9d7d-008171430e20/index.html#r5c1) | [terpene](https://docs.antismash.secondarymetabolites.org/glossary/#terpene) | 609796 | 640886 |  |  |
| [Region 5.2](https://fungismash.secondarymetabolites.org/upload/fungi-d7f1bbd7-b6b0-41dd-9d7d-008171430e20/index.html#r5c2) | [NRPS-like](https://docs.antismash.secondarymetabolites.org/glossary/#nrps-like) | 864181 | 927690 |  |  |
| [Region 5.3](https://fungismash.secondarymetabolites.org/upload/fungi-d7f1bbd7-b6b0-41dd-9d7d-008171430e20/index.html#r5c3) | [terpene](https://docs.antismash.secondarymetabolites.org/glossary/#terpene) | 1091230 | 1122825 |  |  |
| [Region 5.4](https://fungismash.secondarymetabolites.org/upload/fungi-d7f1bbd7-b6b0-41dd-9d7d-008171430e20/index.html#r5c4) | [NRPS](https://docs.antismash.secondarymetabolites.org/glossary/#nrps) | 1390884 | 1454800 |  |  |
| [Region 5.5](https://fungismash.secondarymetabolites.org/upload/fungi-d7f1bbd7-b6b0-41dd-9d7d-008171430e20/index.html#r5c5) | [T1PKS](https://docs.antismash.secondarymetabolites.org/glossary/#t1pks) | 1620370 | 1780739 | Low | [azasperpyranone A/azasperpyranone B/azasperpyranone C/azasperpyranone D/azasperpyranone E/azasperpyranone F/azasperpyranone G/azasperpyranone H](https://mibig.secondarymetabolites.org/go/BGC0002267.2) |
| [Region 5.6](https://fungismash.secondarymetabolites.org/upload/fungi-d7f1bbd7-b6b0-41dd-9d7d-008171430e20/index.html#r5c6) | [NRPS-like](https://docs.antismash.secondarymetabolites.org/glossary/#nrps-like) | 1886292 | 1949544 |  |  |
| [Region 5.7](https://fungismash.secondarymetabolites.org/upload/fungi-d7f1bbd7-b6b0-41dd-9d7d-008171430e20/index.html#r5c7) | [NRPS](https://docs.antismash.secondarymetabolites.org/glossary/#nrps) | 2029962 | 2100560 |  |  |
| [Region 6.1](https://fungismash.secondarymetabolites.org/upload/fungi-d7f1bbd7-b6b0-41dd-9d7d-008171430e20/index.html#r6c1) | NRPS-like,T1PKS | 158450 | 223692 |  |  |
| [Region 6.2](https://fungismash.secondarymetabolites.org/upload/fungi-d7f1bbd7-b6b0-41dd-9d7d-008171430e20/index.html#r6c2) | NRPS-like | 787924 | 850068 | High | [aspulvinone H/aspulvinone B1](https://mibig.secondarymetabolites.org/go/BGC0002274.2) |
| [Region 6.3](https://fungismash.secondarymetabolites.org/upload/fungi-d7f1bbd7-b6b0-41dd-9d7d-008171430e20/index.html#r6c3) | [T1PKS](https://docs.antismash.secondarymetabolites.org/glossary/#t1pks) | 1314013 | 1379803 | Low | [neosartorin](https://mibig.secondarymetabolites.org/go/BGC0001988.3) |
| [Region 7.1](https://fungismash.secondarymetabolites.org/upload/fungi-d7f1bbd7-b6b0-41dd-9d7d-008171430e20/index.html#r7c1) | [T1PKS](https://docs.antismash.secondarymetabolites.org/glossary/#t1pks) | 9478 | 78641 |  |  |
| [Region 7.2](https://fungismash.secondarymetabolites.org/upload/fungi-d7f1bbd7-b6b0-41dd-9d7d-008171430e20/index.html#r7c2) | [NRPS-like](https://docs.antismash.secondarymetabolites.org/glossary/#nrps-like) | 395672 | 459105 |  |  |
| [Region 7.3](https://fungismash.secondarymetabolites.org/upload/fungi-d7f1bbd7-b6b0-41dd-9d7d-008171430e20/index.html#r7c3) | [NRPS-like](https://docs.antismash.secondarymetabolites.org/glossary/#nrps-like) | 1539416 | 1602696 |  |  |
| [Region 7.4](https://fungismash.secondarymetabolites.org/upload/fungi-d7f1bbd7-b6b0-41dd-9d7d-008171430e20/index.html#r7c4) | T1PKS,NRPS-like | 1655800 | 1757225 |  |  |
| [Region 8.1](https://fungismash.secondarymetabolites.org/upload/fungi-d7f1bbd7-b6b0-41dd-9d7d-008171430e20/index.html#r8c1) | [terpene](https://docs.antismash.secondarymetabolites.org/glossary/#terpene) | 258699 | 291750 |  |  |
| [Region 8.2](https://fungismash.secondarymetabolites.org/upload/fungi-d7f1bbd7-b6b0-41dd-9d7d-008171430e20/index.html#r8c2) | [terpene-precursor](https://docs.antismash.secondarymetabolites.org/glossary/#terpene-precursor) | 417853 | 449130 |  |  |
| [Region 8.3](https://fungismash.secondarymetabolites.org/upload/fungi-d7f1bbd7-b6b0-41dd-9d7d-008171430e20/index.html#r8c3) | [T1PKS](https://docs.antismash.secondarymetabolites.org/glossary/#t1pks) | 1297267 | 1402947 | Medium | [scytalone/T3HN](https://mibig.secondarymetabolites.org/go/BGC0002161.2) |
| [Region 9.1](https://fungismash.secondarymetabolites.org/upload/fungi-d7f1bbd7-b6b0-41dd-9d7d-008171430e20/index.html#r9c1) | [isocyanide](https://docs.antismash.secondarymetabolites.org/glossary/#isocyanide) | 690115 | 752384 |  |  |
| [Region 10.1](https://fungismash.secondarymetabolites.org/upload/fungi-d7f1bbd7-b6b0-41dd-9d7d-008171430e20/index.html#r10c1) | [terpene-precursor](https://docs.antismash.secondarymetabolites.org/glossary/#terpene-precursor) | 850213 | 881434 |  |  |
| [Region 11.1](https://fungismash.secondarymetabolites.org/upload/fungi-d7f1bbd7-b6b0-41dd-9d7d-008171430e20/index.html#r11c1) | [NRPS-like](https://docs.antismash.secondarymetabolites.org/glossary/#nrps-like) | 669720 | 734420 |  |  |
| [Region 12.1](https://fungismash.secondarymetabolites.org/upload/fungi-d7f1bbd7-b6b0-41dd-9d7d-008171430e20/index.html#r12c1) | [NRPS-like](https://docs.antismash.secondarymetabolites.org/glossary/#nrps-like) | 615201 | 678770 |  |  |
| [Region 13.1](https://fungismash.secondarymetabolites.org/upload/fungi-d7f1bbd7-b6b0-41dd-9d7d-008171430e20/index.html#r13c1) | [terpene](https://docs.antismash.secondarymetabolites.org/glossary/#terpene) | 572069 | 604053 |  |  |
| [Region 14.1](https://fungismash.secondarymetabolites.org/upload/fungi-d7f1bbd7-b6b0-41dd-9d7d-008171430e20/index.html#r14c1) | [NRPS-like](https://docs.antismash.secondarymetabolites.org/glossary/#nrps-like) | 845066 | 880243 | High | [choline](https://mibig.secondarymetabolites.org/go/BGC0002276.2) |
| [Region 15.1](https://fungismash.secondarymetabolites.org/upload/fungi-d7f1bbd7-b6b0-41dd-9d7d-008171430e20/index.html#r15c1) | [NRPS-like](https://docs.antismash.secondarymetabolites.org/glossary/#nrps-like) | 62149 | 123500 |  |  |
| [Region 15.2](https://fungismash.secondarymetabolites.org/upload/fungi-d7f1bbd7-b6b0-41dd-9d7d-008171430e20/index.html#r15c2) | [NRPS](https://docs.antismash.secondarymetabolites.org/glossary/#nrps) | 642017 | 711987 |  |  |

Tab. S2 Analysis results of secondary metabolic biosynthesis gene clusters of *Gnomoniopsis embeliae*

(Continued)

| **Region** | **Type** | **From** | **To** | **Similarity Confidence** | **Most similar known cluster** |
| --- | --- | --- | --- | --- | --- |
| [Region 16.1](https://fungismash.secondarymetabolites.org/upload/fungi-d7f1bbd7-b6b0-41dd-9d7d-008171430e20/index.html#r16c1) | [T1PKS](https://docs.antismash.secondarymetabolites.org/glossary/#t1pks) | 666809 | 734651 |  |  |
| [Region 19.1](https://fungismash.secondarymetabolites.org/upload/fungi-d7f1bbd7-b6b0-41dd-9d7d-008171430e20/index.html#r19c1) | [T1PKS](https://docs.antismash.secondarymetabolites.org/glossary/#t1pks) | 1 | 43290 |  |  |
| [Region 22.1](https://fungismash.secondarymetabolites.org/upload/fungi-d7f1bbd7-b6b0-41dd-9d7d-008171430e20/index.html#r22c1) | [T1PKS](https://docs.antismash.secondarymetabolites.org/glossary/#t1pks) | 460326 | 528804 |  |  |
| [Region 23.1](https://fungismash.secondarymetabolites.org/upload/fungi-d7f1bbd7-b6b0-41dd-9d7d-008171430e20/index.html#r23c1) | NRPS-like,T1PKS | 123535 | 200497 |  |  |
| [Region 24.1](https://fungismash.secondarymetabolites.org/upload/fungi-d7f1bbd7-b6b0-41dd-9d7d-008171430e20/index.html#r24c1) | [T1PKS](https://docs.antismash.secondarymetabolites.org/glossary/#t1pks) | 274736 | 342403 |  |  |
| [Region 24.2](https://fungismash.secondarymetabolites.org/upload/fungi-d7f1bbd7-b6b0-41dd-9d7d-008171430e20/index.html#r24c2) | [T1PKS](https://docs.antismash.secondarymetabolites.org/glossary/#t1pks) | 413794 | 484285 |  |  |
| [Region 25.1](https://fungismash.secondarymetabolites.org/upload/fungi-d7f1bbd7-b6b0-41dd-9d7d-008171430e20/index.html#r25c1) | [T1PKS](https://docs.antismash.secondarymetabolites.org/glossary/#t1pks) | 47417 | 118947 | High | [alternapyrone](https://mibig.secondarymetabolites.org/go/BGC0000012.3) |
| [Region 25.2](https://fungismash.secondarymetabolites.org/upload/fungi-d7f1bbd7-b6b0-41dd-9d7d-008171430e20/index.html#r25c2) | [NRPS](https://docs.antismash.secondarymetabolites.org/glossary/#nrps) | 215562 | 289773 |  |  |
| [Region 27.1](https://fungismash.secondarymetabolites.org/upload/fungi-d7f1bbd7-b6b0-41dd-9d7d-008171430e20/index.html#r27c1) | [terpene](https://docs.antismash.secondarymetabolites.org/glossary/#terpene) | 353903 | 386330 | High | [clavaric acid](https://mibig.secondarymetabolites.org/go/BGC0001248.3) |
| [Region 29.1](https://fungismash.secondarymetabolites.org/upload/fungi-d7f1bbd7-b6b0-41dd-9d7d-008171430e20/index.html#r29c1) | [terpene](https://docs.antismash.secondarymetabolites.org/glossary/#terpene) | 49325 | 80875 |  |  |
| [Region 29.2](https://fungismash.secondarymetabolites.org/upload/fungi-d7f1bbd7-b6b0-41dd-9d7d-008171430e20/index.html#r29c2) | [other](https://docs.antismash.secondarymetabolites.org/glossary/#other) | 251010 | 318254 |  |  |
| [Region 30.1](https://fungismash.secondarymetabolites.org/upload/fungi-d7f1bbd7-b6b0-41dd-9d7d-008171430e20/index.html#r30c1) | NRP-metallophore,NRPS | 82862 | 161271 |  |  |
| [Region 30.2](https://fungismash.secondarymetabolites.org/upload/fungi-d7f1bbd7-b6b0-41dd-9d7d-008171430e20/index.html#r30c2) | [T3PKS](https://docs.antismash.secondarymetabolites.org/glossary/#t3pks) | 232551 | 294091 |  |  |
| [Region 31.1](https://fungismash.secondarymetabolites.org/upload/fungi-d7f1bbd7-b6b0-41dd-9d7d-008171430e20/index.html#r31c1) | [NRPS](https://docs.antismash.secondarymetabolites.org/glossary/#nrps) | 206256 | 295227 |  |  |
| [Region 33.1](https://fungismash.secondarymetabolites.org/upload/fungi-d7f1bbd7-b6b0-41dd-9d7d-008171430e20/index.html#r33c1) | NRPS-like,T1PKS | 257243 | 295715 |  |  |
| [Region 34.1](https://fungismash.secondarymetabolites.org/upload/fungi-d7f1bbd7-b6b0-41dd-9d7d-008171430e20/index.html#r34c1) | [T1PKS](https://docs.antismash.secondarymetabolites.org/glossary/#t1pks) | 184340 | 265841 | Low | [tricholignan A](https://mibig.secondarymetabolites.org/go/BGC0001854.4) |
| [Region 36.1](https://fungismash.secondarymetabolites.org/upload/fungi-d7f1bbd7-b6b0-41dd-9d7d-008171430e20/index.html#r36c1) | NRPS-like,T1PKS | 8421 | 81113 |  |  |
| [Region 37.1](https://fungismash.secondarymetabolites.org/upload/fungi-d7f1bbd7-b6b0-41dd-9d7d-008171430e20/index.html#r37c1) | T1PKS,indole | 93465 | 161619 | Medium | [trichoxide](https://mibig.secondarymetabolites.org/go/BGC0002233.3) |
| [Region 42.1](https://fungismash.secondarymetabolites.org/upload/fungi-d7f1bbd7-b6b0-41dd-9d7d-008171430e20/index.html#r42c1) | NRPS-like,NRPS,T1PKS | 1 | 111886 |  |  |
| [Region 50.1](https://fungismash.secondarymetabolites.org/upload/fungi-d7f1bbd7-b6b0-41dd-9d7d-008171430e20/index.html#r50c1) | [T1PKS](https://docs.antismash.secondarymetabolites.org/glossary/#t1pks) | 1 | 47295 |  |  |
| [Region 57.1](https://fungismash.secondarymetabolites.org/upload/fungi-d7f1bbd7-b6b0-41dd-9d7d-008171430e20/index.html#r57c1) | [terpene](https://docs.antismash.secondarymetabolites.org/glossary/#terpene) | 6738 | 37739 | Medium | [HEx-tc1 terpene](https://mibig.secondarymetabolites.org/go/BGC0002216.3) |
| [Region 58.1](https://fungismash.secondarymetabolites.org/upload/fungi-d7f1bbd7-b6b0-41dd-9d7d-008171430e20/index.html#r58c1) | [NRPS-like](https://docs.antismash.secondarymetabolites.org/glossary/#nrps-like) | 1 | 43080 |  |  |
| [Region 60.1](https://fungismash.secondarymetabolites.org/upload/fungi-d7f1bbd7-b6b0-41dd-9d7d-008171430e20/index.html#r60c1) | [NRPS](https://docs.antismash.secondarymetabolites.org/glossary/#nrps) | 1 | 38844 |  |  |
| [Region 61.1](https://fungismash.secondarymetabolites.org/upload/fungi-d7f1bbd7-b6b0-41dd-9d7d-008171430e20/index.html#r61c1) | [T1PKS](https://docs.antismash.secondarymetabolites.org/glossary/#t1pks) | 1 | 37258 |  |  |
| [Region 78.1](https://fungismash.secondarymetabolites.org/upload/fungi-d7f1bbd7-b6b0-41dd-9d7d-008171430e20/index.html#r78c1) | [T1PKS](https://docs.antismash.secondarymetabolites.org/glossary/#t1pks) | 1 | 19723 |  |  |
| [Region 83.1](https://fungismash.secondarymetabolites.org/upload/fungi-d7f1bbd7-b6b0-41dd-9d7d-008171430e20/index.html#r83c1) | [terpene](https://docs.antismash.secondarymetabolites.org/glossary/#terpene) | 1 | 17685 |  |  |

Tab. S3 Analysis results of secondary metabolic biosynthesis gene clusters of *Gnomoniopsis yunvfengensis*

| **Region** | **Type** | **From** | **To** | **Similarity Confidence** | **Most similar known cluster** |
| --- | --- | --- | --- | --- | --- |
| [Region 1.1](https://fungismash.secondarymetabolites.org/upload/fungi-bb5eddb7-4374-4030-bf56-a772c1e894ee/index.html#r1c1) | [NRPS-like](https://docs.antismash.secondarymetabolites.org/glossary/#nrps-like) | 969227 | 1032536 |  |  |
| [Region 1.2](https://fungismash.secondarymetabolites.org/upload/fungi-bb5eddb7-4374-4030-bf56-a772c1e894ee/index.html#r1c2) | [phosphonate](https://docs.antismash.secondarymetabolites.org/glossary/#phosphonate) | 1425981 | 1442383 |  |  |
| [Region 1.3](https://fungismash.secondarymetabolites.org/upload/fungi-bb5eddb7-4374-4030-bf56-a772c1e894ee/index.html#r1c3) | [indole](https://docs.antismash.secondarymetabolites.org/glossary/#indole) | 1587744 | 1618718 |  |  |
| [Region 1.4](https://fungismash.secondarymetabolites.org/upload/fungi-bb5eddb7-4374-4030-bf56-a772c1e894ee/index.html#r1c4) | [NRPS](https://docs.antismash.secondarymetabolites.org/glossary/#nrps) | 2411343 | 2475254 |  |  |
| [Region 1.5](https://fungismash.secondarymetabolites.org/upload/fungi-bb5eddb7-4374-4030-bf56-a772c1e894ee/index.html#r1c5) | T1PKS,terpene | 2632138 | 2730740 | Medium | [harziphilone/t22azaphilone/isoharziphilone-1/isoharziphilone-2/compound 4/compound 1](https://mibig.secondarymetabolites.org/go/BGC0002206.2) |
| [Region 1.6](https://fungismash.secondarymetabolites.org/upload/fungi-bb5eddb7-4374-4030-bf56-a772c1e894ee/index.html#r1c6) | [NRPS](https://docs.antismash.secondarymetabolites.org/glossary/#nrps) | 3003053 | 3073612 |  |  |
| [Region 1.7](https://fungismash.secondarymetabolites.org/upload/fungi-bb5eddb7-4374-4030-bf56-a772c1e894ee/index.html#r1c7) | NRPS-like,NRPS,T1PKS | 3215535 | 3329311 |  |  |
| [Region 2.1](https://fungismash.secondarymetabolites.org/upload/fungi-bb5eddb7-4374-4030-bf56-a772c1e894ee/index.html#r2c1) | [NI-siderophore](https://docs.antismash.secondarymetabolites.org/glossary/#ni-siderophore) | 1035876 | 1079998 |  |  |
| [Region 2.2](https://fungismash.secondarymetabolites.org/upload/fungi-bb5eddb7-4374-4030-bf56-a772c1e894ee/index.html#r2c2) | NRPS-like,terpene | 1999066 | 2064398 |  |  |
| [Region 2.3](https://fungismash.secondarymetabolites.org/upload/fungi-bb5eddb7-4374-4030-bf56-a772c1e894ee/index.html#r2c3) | terpene,T1PKS | 3219985 | 3303847 | Low | [betaenone A/betaenone B/betaenone C](https://mibig.secondarymetabolites.org/go/BGC0001264.3) |
| [Region 3.1](https://fungismash.secondarymetabolites.org/upload/fungi-bb5eddb7-4374-4030-bf56-a772c1e894ee/index.html#r3c1) | [indole](https://docs.antismash.secondarymetabolites.org/glossary/#indole) | 816704 | 848062 |  |  |
| [Region 3.2](https://fungismash.secondarymetabolites.org/upload/fungi-bb5eddb7-4374-4030-bf56-a772c1e894ee/index.html#r3c2) | [terpene](https://docs.antismash.secondarymetabolites.org/glossary/#terpene) | 901469 | 932649 |  |  |
| [Region 4.1](https://fungismash.secondarymetabolites.org/upload/fungi-bb5eddb7-4374-4030-bf56-a772c1e894ee/index.html#r4c1) | T1PKS,indole | 511941 | 580094 |  |  |
| [Region 4.2](https://fungismash.secondarymetabolites.org/upload/fungi-bb5eddb7-4374-4030-bf56-a772c1e894ee/index.html#r4c2) | [NRPS-like](https://docs.antismash.secondarymetabolites.org/glossary/#nrps-like) | 1792052 | 1855440 |  |  |
| [Region 4.3](https://fungismash.secondarymetabolites.org/upload/fungi-bb5eddb7-4374-4030-bf56-a772c1e894ee/index.html#r4c3) | [T1PKS](https://docs.antismash.secondarymetabolites.org/glossary/#t1pks) | 2013682 | 2072666 | High | [alternapyrone](https://mibig.secondarymetabolites.org/go/BGC0000012.3) |
| [Region 5.1](https://fungismash.secondarymetabolites.org/upload/fungi-bb5eddb7-4374-4030-bf56-a772c1e894ee/index.html#r5c1) | [terpene](https://docs.antismash.secondarymetabolites.org/glossary/#terpene) | 1399828 | 1431210 |  |  |
| [Region 5.2](https://fungismash.secondarymetabolites.org/upload/fungi-bb5eddb7-4374-4030-bf56-a772c1e894ee/index.html#r5c2) | [terpene-precursor](https://docs.antismash.secondarymetabolites.org/glossary/#terpene-precursor) | 1556251 | 1587522 |  |  |
| [Region 6.1](https://fungismash.secondarymetabolites.org/upload/fungi-bb5eddb7-4374-4030-bf56-a772c1e894ee/index.html#r6c1) | [NRPS-like](https://docs.antismash.secondarymetabolites.org/glossary/#nrps-like) | 156195 | 219612 |  |  |
| [Region 6.2](https://fungismash.secondarymetabolites.org/upload/fungi-bb5eddb7-4374-4030-bf56-a772c1e894ee/index.html#r6c2) | NRPS-like,T1PKS | 633825 | 699061 |  |  |
| [Region 6.3](https://fungismash.secondarymetabolites.org/upload/fungi-bb5eddb7-4374-4030-bf56-a772c1e894ee/index.html#r6c3) | [terpene](https://docs.antismash.secondarymetabolites.org/glossary/#terpene) | 1611147 | 1642978 |  |  |
| [Region 7.1](https://fungismash.secondarymetabolites.org/upload/fungi-bb5eddb7-4374-4030-bf56-a772c1e894ee/index.html#r7c1) | [NRPS](https://docs.antismash.secondarymetabolites.org/glossary/#nrps) | 118537 | 192742 |  |  |
| [Region 7.2](https://fungismash.secondarymetabolites.org/upload/fungi-bb5eddb7-4374-4030-bf56-a772c1e894ee/index.html#r7c2) | [isocyanide](https://docs.antismash.secondarymetabolites.org/glossary/#isocyanide) | 1001004 | 1063230 |  |  |
| [Region 8.1](https://fungismash.secondarymetabolites.org/upload/fungi-bb5eddb7-4374-4030-bf56-a772c1e894ee/index.html#r8c1) | [NRPS-like](https://docs.antismash.secondarymetabolites.org/glossary/#nrps-like) | 165 | 65322 |  |  |
| [Region 8.2](https://fungismash.secondarymetabolites.org/upload/fungi-bb5eddb7-4374-4030-bf56-a772c1e894ee/index.html#r8c2) | [terpene](https://docs.antismash.secondarymetabolites.org/glossary/#terpene) | 87186 | 119592 | High | [clavaric acid](https://mibig.secondarymetabolites.org/go/BGC0001248.3) |
| [Region 8.3](https://fungismash.secondarymetabolites.org/upload/fungi-bb5eddb7-4374-4030-bf56-a772c1e894ee/index.html#r8c3) | [terpene](https://docs.antismash.secondarymetabolites.org/glossary/#terpene) | 868860 | 899828 |  |  |
| [Region 8.4](https://fungismash.secondarymetabolites.org/upload/fungi-bb5eddb7-4374-4030-bf56-a772c1e894ee/index.html#r8c4) | [terpene-precursor](https://docs.antismash.secondarymetabolites.org/glossary/#terpene-precursor) | 1486917 | 1518144 |  |  |
| [Region 9.1](https://fungismash.secondarymetabolites.org/upload/fungi-bb5eddb7-4374-4030-bf56-a772c1e894ee/index.html#r9c1) | NRPS,T1PKS,terpene | 109845 | 195652 |  |  |
| [Region 9.2](https://fungismash.secondarymetabolites.org/upload/fungi-bb5eddb7-4374-4030-bf56-a772c1e894ee/index.html#r9c2) | [terpene](https://docs.antismash.secondarymetabolites.org/glossary/#terpene) | 642788 | 673871 |  |  |
| [Region 9.3](https://fungismash.secondarymetabolites.org/upload/fungi-bb5eddb7-4374-4030-bf56-a772c1e894ee/index.html#r9c3) | [NRPS-like](https://docs.antismash.secondarymetabolites.org/glossary/#nrps-like) | 792249 | 855585 |  |  |
| [Region 10.1](https://fungismash.secondarymetabolites.org/upload/fungi-bb5eddb7-4374-4030-bf56-a772c1e894ee/index.html#r10c1) | [T1PKS](https://docs.antismash.secondarymetabolites.org/glossary/#t1pks) | 750519 | 817996 |  |  |
| [Region 12.1](https://fungismash.secondarymetabolites.org/upload/fungi-bb5eddb7-4374-4030-bf56-a772c1e894ee/index.html#r12c1) | [NRPS-like](https://docs.antismash.secondarymetabolites.org/glossary/#nrps-like) | 1076630 | 1140589 | High | [choline](https://mibig.secondarymetabolites.org/go/BGC0002276.2) |
| [Region 13.1](https://fungismash.secondarymetabolites.org/upload/fungi-bb5eddb7-4374-4030-bf56-a772c1e894ee/index.html#r13c1) | [NRPS-like](https://docs.antismash.secondarymetabolites.org/glossary/#nrps-like) | 84009 | 147860 |  |  |
| [Region 13.2](https://fungismash.secondarymetabolites.org/upload/fungi-bb5eddb7-4374-4030-bf56-a772c1e894ee/index.html#r13c2) | [terpene](https://docs.antismash.secondarymetabolites.org/glossary/#terpene) | 428251 | 459877 | Low | [squalestatin S1](https://mibig.secondarymetabolites.org/go/BGC0001839.3) |
| [Region 13.3](https://fungismash.secondarymetabolites.org/upload/fungi-bb5eddb7-4374-4030-bf56-a772c1e894ee/index.html#r13c3) | NRP-metallophore,NRPS | 639087 | 717572 |  |  |

Tab. S3 Analysis results of secondary metabolic biosynthesis gene clusters of *Gnomoniopsis yunvfengensis*

(Continued)

| **Region** | **Type** | **From** | **To** | **Similarity Confidence** | **Most similar known cluster** |
| --- | --- | --- | --- | --- | --- |
| [Region 13.4](https://fungismash.secondarymetabolites.org/upload/fungi-bb5eddb7-4374-4030-bf56-a772c1e894ee/index.html#r13c4) | [T3PKS](https://docs.antismash.secondarymetabolites.org/glossary/#t3pks) | 787937 | 849484 |  |  |
| [Region 14.1](https://fungismash.secondarymetabolites.org/upload/fungi-bb5eddb7-4374-4030-bf56-a772c1e894ee/index.html#r14c1) | [T1PKS](https://docs.antismash.secondarymetabolites.org/glossary/#t1pks) | 534101 | 600764 | Medium | [scytalone/T3HN](https://mibig.secondarymetabolites.org/go/BGC0002161.2) |
| [Region 15.1](https://fungismash.secondarymetabolites.org/upload/fungi-bb5eddb7-4374-4030-bf56-a772c1e894ee/index.html#r15c1) | [T1PKS](https://docs.antismash.secondarymetabolites.org/glossary/#t1pks) | 650062 | 755763 | Medium | [neosartorin](https://mibig.secondarymetabolites.org/go/BGC0001988.3) |
| [Region 16.1](https://fungismash.secondarymetabolites.org/upload/fungi-bb5eddb7-4374-4030-bf56-a772c1e894ee/index.html#r16c1) | [T1PKS](https://docs.antismash.secondarymetabolites.org/glossary/#t1pks) | 446435 | 514269 |  |  |
| [Region 16.2](https://fungismash.secondarymetabolites.org/upload/fungi-bb5eddb7-4374-4030-bf56-a772c1e894ee/index.html#r16c2) | [NRPS-like](https://docs.antismash.secondarymetabolites.org/glossary/#nrps-like) | 660541 | 723824 |  |  |
| [Region 16.3](https://fungismash.secondarymetabolites.org/upload/fungi-bb5eddb7-4374-4030-bf56-a772c1e894ee/index.html#r16c3) | [T1PKS](https://docs.antismash.secondarymetabolites.org/glossary/#t1pks) | 775730 | 914088 | Low | [hypothemycin](https://mibig.secondarymetabolites.org/go/BGC0000077.3) |
| [Region 17.1](https://fungismash.secondarymetabolites.org/upload/fungi-bb5eddb7-4374-4030-bf56-a772c1e894ee/index.html#r17c1) | [NRPS-like](https://docs.antismash.secondarymetabolites.org/glossary/#nrps-like) | 529695 | 594659 |  |  |
| [Region 21.1](https://fungismash.secondarymetabolites.org/upload/fungi-bb5eddb7-4374-4030-bf56-a772c1e894ee/index.html#r21c1) | [T1PKS](https://docs.antismash.secondarymetabolites.org/glossary/#t1pks) | 141717 | 209426 |  |  |
| [Region 21.2](https://fungismash.secondarymetabolites.org/upload/fungi-bb5eddb7-4374-4030-bf56-a772c1e894ee/index.html#r21c2) | [T1PKS](https://docs.antismash.secondarymetabolites.org/glossary/#t1pks) | 283828 | 354328 |  |  |
| [Region 21.3](https://fungismash.secondarymetabolites.org/upload/fungi-bb5eddb7-4374-4030-bf56-a772c1e894ee/index.html#r21c3) | NRPS-like,T1PKS | 521167 | 593635 |  |  |
| [Region 23.1](https://fungismash.secondarymetabolites.org/upload/fungi-bb5eddb7-4374-4030-bf56-a772c1e894ee/index.html#r23c1) | [T1PKS](https://docs.antismash.secondarymetabolites.org/glossary/#t1pks) | 112121 | 180657 |  |  |
| [Region 23.2](https://fungismash.secondarymetabolites.org/upload/fungi-bb5eddb7-4374-4030-bf56-a772c1e894ee/index.html#r23c2) | [NRPS](https://docs.antismash.secondarymetabolites.org/glossary/#nrps) | 188614 | 258568 |  |  |
| [Region 23.3](https://fungismash.secondarymetabolites.org/upload/fungi-bb5eddb7-4374-4030-bf56-a772c1e894ee/index.html#r23c3) | [terpene](https://docs.antismash.secondarymetabolites.org/glossary/#terpene) | 272868 | 304099 |  |  |
| [Region 23.4](https://fungismash.secondarymetabolites.org/upload/fungi-bb5eddb7-4374-4030-bf56-a772c1e894ee/index.html#r23c4) | T1PKS,NRPS-like | 449633 | 551269 |  |  |
| [Region 24.1](https://fungismash.secondarymetabolites.org/upload/fungi-bb5eddb7-4374-4030-bf56-a772c1e894ee/index.html#r24c1) | [terpene](https://docs.antismash.secondarymetabolites.org/glossary/#terpene) | 93833 | 125801 |  |  |
| [Region 24.2](https://fungismash.secondarymetabolites.org/upload/fungi-bb5eddb7-4374-4030-bf56-a772c1e894ee/index.html#r24c2) | [T1PKS](https://docs.antismash.secondarymetabolites.org/glossary/#t1pks) | 547894 | 611467 |  |  |
| [Region 25.1](https://fungismash.secondarymetabolites.org/upload/fungi-bb5eddb7-4374-4030-bf56-a772c1e894ee/index.html#r25c1) | T1PKS,NRPS-like,indole | 437582 | 507167 | High | [cichorine](https://mibig.secondarymetabolites.org/go/BGC0000037.4) |
| [Region 27.1](https://fungismash.secondarymetabolites.org/upload/fungi-bb5eddb7-4374-4030-bf56-a772c1e894ee/index.html#r27c1) | [T1PKS](https://docs.antismash.secondarymetabolites.org/glossary/#t1pks) | 438853 | 505015 | Low | [flavoglaucin](https://mibig.secondarymetabolites.org/go/BGC0002234.3) |
| [Region 31.1](https://fungismash.secondarymetabolites.org/upload/fungi-bb5eddb7-4374-4030-bf56-a772c1e894ee/index.html#r31c1) | [other](https://docs.antismash.secondarymetabolites.org/glossary/#other) | 154453 | 215367 |  |  |
| [Region 31.2](https://fungismash.secondarymetabolites.org/upload/fungi-bb5eddb7-4374-4030-bf56-a772c1e894ee/index.html#r31c2) | [terpene](https://docs.antismash.secondarymetabolites.org/glossary/#terpene) | 390395 | 421939 |  |  |
| [Region 32.1](https://fungismash.secondarymetabolites.org/upload/fungi-bb5eddb7-4374-4030-bf56-a772c1e894ee/index.html#r32c1) | [terpene](https://docs.antismash.secondarymetabolites.org/glossary/#terpene) | 1 | 20833 |  |  |
| [Region 32.2](https://fungismash.secondarymetabolites.org/upload/fungi-bb5eddb7-4374-4030-bf56-a772c1e894ee/index.html#r32c2) | NRPS-like,T1PKS | 352846 | 412643 |  |  |

Tab. S4 Analysis results of secondary metabolic biosynthesis gene clusters of *Gnomoniopsis yonganensis*

| **Region** | **Type** | **From** | **To** | **Similarity Confidence** | **Most similar known cluster** |
| --- | --- | --- | --- | --- | --- |
| [Region 1.1](https://fungismash.secondarymetabolites.org/upload/fungi-d2dcf881-ff87-4d9e-a830-770ef1dbdd37/index.html#r1c1) | [T1PKS](https://docs.antismash.secondarymetabolites.org/glossary/#t1pks) | 124139 | 189940 | Medium | [neosartorin](https://mibig.secondarymetabolites.org/go/BGC0001988.3) |
| [Region 1.2](https://fungismash.secondarymetabolites.org/upload/fungi-d2dcf881-ff87-4d9e-a830-770ef1dbdd37/index.html#r1c2) | [NRPS-like](https://docs.antismash.secondarymetabolites.org/glossary/#nrps-like) | 748182 | 811532 |  |  |
| [Region 1.3](https://fungismash.secondarymetabolites.org/upload/fungi-d2dcf881-ff87-4d9e-a830-770ef1dbdd37/index.html#r1c3) | T1PKS,NRPS-like | 822450 | 925935 | Low | [hypothemycin](https://mibig.secondarymetabolites.org/go/BGC0000077.3) |
| [Region 1.4](https://fungismash.secondarymetabolites.org/upload/fungi-d2dcf881-ff87-4d9e-a830-770ef1dbdd37/index.html#r1c4) | T1PKS,phosphonate | 1030127 | 1129733 | Low | [tricholignan A](https://mibig.secondarymetabolites.org/go/BGC0001854.4) |
| [Region 2.1](https://fungismash.secondarymetabolites.org/upload/fungi-d2dcf881-ff87-4d9e-a830-770ef1dbdd37/index.html#r2c1) | NRPS,T1PKS | 296375 | 408538 | Low | [trichoxide](https://mibig.secondarymetabolites.org/go/BGC0002233.3) |
| [Region 3.1](https://fungismash.secondarymetabolites.org/upload/fungi-d2dcf881-ff87-4d9e-a830-770ef1dbdd37/index.html#r3c1) | [NRPS](https://docs.antismash.secondarymetabolites.org/glossary/#nrps) | 1 | 57449 | High | [gliovirin](https://mibig.secondarymetabolites.org/go/BGC0001609.4) |
| [Region 3.2](https://fungismash.secondarymetabolites.org/upload/fungi-d2dcf881-ff87-4d9e-a830-770ef1dbdd37/index.html#r3c2) | [NRPS-like](https://docs.antismash.secondarymetabolites.org/glossary/#nrps-like) | 1082854 | 1146434 |  |  |
| [Region 4.1](https://fungismash.secondarymetabolites.org/upload/fungi-d2dcf881-ff87-4d9e-a830-770ef1dbdd37/index.html#r4c1) | [terpene-precursor](https://docs.antismash.secondarymetabolites.org/glossary/#terpene-precursor) | 71856 | 103145 |  |  |
| [Region 4.2](https://fungismash.secondarymetabolites.org/upload/fungi-d2dcf881-ff87-4d9e-a830-770ef1dbdd37/index.html#r4c2) | [T1PKS](https://docs.antismash.secondarymetabolites.org/glossary/#t1pks) | 859119 | 925816 | Medium | [scytalone/T3HN](https://mibig.secondarymetabolites.org/go/BGC0002161.2) |
| [Region 5.1](https://fungismash.secondarymetabolites.org/upload/fungi-d2dcf881-ff87-4d9e-a830-770ef1dbdd37/index.html#r5c1) | [T1PKS](https://docs.antismash.secondarymetabolites.org/glossary/#t1pks) | 49109 | 117388 |  |  |
| [Region 5.2](https://fungismash.secondarymetabolites.org/upload/fungi-d2dcf881-ff87-4d9e-a830-770ef1dbdd37/index.html#r5c2) | T1PKS,terpene,indole,isocyanide | 675352 | 776204 |  |  |
| [Region 5.3](https://fungismash.secondarymetabolites.org/upload/fungi-d2dcf881-ff87-4d9e-a830-770ef1dbdd37/index.html#r5c3) | [T1PKS](https://docs.antismash.secondarymetabolites.org/glossary/#t1pks) | 811648 | 882819 | High | [alternapyrone](https://mibig.secondarymetabolites.org/go/BGC0000012.3) |
| [Region 5.4](https://fungismash.secondarymetabolites.org/upload/fungi-d2dcf881-ff87-4d9e-a830-770ef1dbdd37/index.html#r5c4) | [indole](https://docs.antismash.secondarymetabolites.org/glossary/#indole) | 1248912 | 1279823 |  |  |
| [Region 8.1](https://fungismash.secondarymetabolites.org/upload/fungi-d2dcf881-ff87-4d9e-a830-770ef1dbdd37/index.html#r8c1) | NRPS,indole | 135701 | 212311 |  |  |
| [Region 8.2](https://fungismash.secondarymetabolites.org/upload/fungi-d2dcf881-ff87-4d9e-a830-770ef1dbdd37/index.html#r8c2) | [NRPS](https://docs.antismash.secondarymetabolites.org/glossary/#nrps) | 798906 | 868819 |  |  |
| [Region 8.3](https://fungismash.secondarymetabolites.org/upload/fungi-d2dcf881-ff87-4d9e-a830-770ef1dbdd37/index.html#r8c3) | [terpene](https://docs.antismash.secondarymetabolites.org/glossary/#terpene) | 1114191 | 1145450 |  |  |
| [Region 9.1](https://fungismash.secondarymetabolites.org/upload/fungi-d2dcf881-ff87-4d9e-a830-770ef1dbdd37/index.html#r9c1) | [NRPS](https://docs.antismash.secondarymetabolites.org/glossary/#nrps) | 538089 | 601900 |  |  |
| [Region 11.1](https://fungismash.secondarymetabolites.org/upload/fungi-d2dcf881-ff87-4d9e-a830-770ef1dbdd37/index.html#r11c1) | NRPS-like,T1PKS | 146405 | 238196 | Low | [oxyjavanicin](https://mibig.secondarymetabolites.org/go/BGC0001242.3) |
| [Region 11.2](https://fungismash.secondarymetabolites.org/upload/fungi-d2dcf881-ff87-4d9e-a830-770ef1dbdd37/index.html#r11c2) | [T3PKS](https://docs.antismash.secondarymetabolites.org/glossary/#t3pks) | 326497 | 388018 |  |  |
| [Region 11.3](https://fungismash.secondarymetabolites.org/upload/fungi-d2dcf881-ff87-4d9e-a830-770ef1dbdd37/index.html#r11c3) | [NRPS-like](https://docs.antismash.secondarymetabolites.org/glossary/#nrps-like) | 927313 | 990640 |  |  |
| [Region 13.1](https://fungismash.secondarymetabolites.org/upload/fungi-d2dcf881-ff87-4d9e-a830-770ef1dbdd37/index.html#r13c1) | [other](https://docs.antismash.secondarymetabolites.org/glossary/#other) | 341847 | 402761 |  |  |
| [Region 14.1](https://fungismash.secondarymetabolites.org/upload/fungi-d2dcf881-ff87-4d9e-a830-770ef1dbdd37/index.html#r14c1) | T1PKS,terpene-precursor | 1 | 62342 | Low | [terpestacin/preterpestacin 3/preterpestacin 2/preterpestacin 1](https://mibig.secondarymetabolites.org/go/BGC0002245.2) |
| [Region 14.2](https://fungismash.secondarymetabolites.org/upload/fungi-d2dcf881-ff87-4d9e-a830-770ef1dbdd37/index.html#r14c2) | [NRPS-like](https://docs.antismash.secondarymetabolites.org/glossary/#nrps-like) | 165894 | 229315 |  |  |
| [Region 14.3](https://fungismash.secondarymetabolites.org/upload/fungi-d2dcf881-ff87-4d9e-a830-770ef1dbdd37/index.html#r14c3) | [terpene](https://docs.antismash.secondarymetabolites.org/glossary/#terpene) | 568037 | 600003 |  |  |
| [Region 14.4](https://fungismash.secondarymetabolites.org/upload/fungi-d2dcf881-ff87-4d9e-a830-770ef1dbdd37/index.html#r14c4) | [NRPS-like](https://docs.antismash.secondarymetabolites.org/glossary/#nrps-like) | 721207 | 784702 |  |  |
| [Region 14.5](https://fungismash.secondarymetabolites.org/upload/fungi-d2dcf881-ff87-4d9e-a830-770ef1dbdd37/index.html#r14c5) | [T1PKS](https://docs.antismash.secondarymetabolites.org/glossary/#t1pks) | 807987 | 874883 |  |  |
| [Region 19.1](https://fungismash.secondarymetabolites.org/upload/fungi-d2dcf881-ff87-4d9e-a830-770ef1dbdd37/index.html#r19c1) | [terpene](https://docs.antismash.secondarymetabolites.org/glossary/#terpene) | 373704 | 405068 |  |  |
| [Region 19.2](https://fungismash.secondarymetabolites.org/upload/fungi-d2dcf881-ff87-4d9e-a830-770ef1dbdd37/index.html#r19c2) | [NRPS-like](https://docs.antismash.secondarymetabolites.org/glossary/#nrps-like) | 644888 | 695382 |  |  |
| [Region 20.1](https://fungismash.secondarymetabolites.org/upload/fungi-d2dcf881-ff87-4d9e-a830-770ef1dbdd37/index.html#r20c1) | [T1PKS](https://docs.antismash.secondarymetabolites.org/glossary/#t1pks) | 40162 | 109748 | Low | [monacolin K](https://mibig.secondarymetabolites.org/go/BGC0000098.3) |
| [Region 20.2](https://fungismash.secondarymetabolites.org/upload/fungi-d2dcf881-ff87-4d9e-a830-770ef1dbdd37/index.html#r20c2) | [T1PKS](https://docs.antismash.secondarymetabolites.org/glossary/#t1pks) | 526705 | 595811 | High | [betaenone C/probetaenone I/stemphyloxin II/compound 4/compound 6/compound 7/compound 9/compound 3/dehydroprobetaenone I/compound 5](https://mibig.secondarymetabolites.org/go/BGC0002165.3) |
| [Region 21.1](https://fungismash.secondarymetabolites.org/upload/fungi-d2dcf881-ff87-4d9e-a830-770ef1dbdd37/index.html#r21c1) | [NRPS-like](https://docs.antismash.secondarymetabolites.org/glossary/#nrps-like) | 1 | 54913 |  |  |

Tab. S4 Analysis results of secondary metabolic biosynthesis gene clusters of *Gnomoniopsis yonganensis* (Continued)

| **Region** | **Type** | **From** | **To** | **Similarity Confidence** | **Most similar known cluster** |
| --- | --- | --- | --- | --- | --- |
| [Region 21.2](https://fungismash.secondarymetabolites.org/upload/fungi-d2dcf881-ff87-4d9e-a830-770ef1dbdd37/index.html#r21c2) | [terpene](https://docs.antismash.secondarymetabolites.org/glossary/#terpene) | 60695 | 93194 | High | [clavaric acid](https://mibig.secondarymetabolites.org/go/BGC0001248.3) |
| [Region 21.3](https://fungismash.secondarymetabolites.org/upload/fungi-d2dcf881-ff87-4d9e-a830-770ef1dbdd37/index.html#r21c3) | [terpene-precursor](https://docs.antismash.secondarymetabolites.org/glossary/#terpene-precursor) | 386259 | 417459 |  |  |
| [Region 22.1](https://fungismash.secondarymetabolites.org/upload/fungi-d2dcf881-ff87-4d9e-a830-770ef1dbdd37/index.html#r22c1) | NRPS-like,NRPS | 211449 | 299927 |  |  |
| [Region 23.1](https://fungismash.secondarymetabolites.org/upload/fungi-d2dcf881-ff87-4d9e-a830-770ef1dbdd37/index.html#r23c1) | NRP-metallophore,NRPS | 14386 | 92816 |  |  |
| [Region 24.1](https://fungismash.secondarymetabolites.org/upload/fungi-d2dcf881-ff87-4d9e-a830-770ef1dbdd37/index.html#r24c1) | [terpene](https://docs.antismash.secondarymetabolites.org/glossary/#terpene) | 281481 | 313002 |  |  |
| [Region 26.1](https://fungismash.secondarymetabolites.org/upload/fungi-d2dcf881-ff87-4d9e-a830-770ef1dbdd37/index.html#r26c1) | NRPS-like,T1PKS | 33369 | 98734 |  |  |
| [Region 27.1](https://fungismash.secondarymetabolites.org/upload/fungi-d2dcf881-ff87-4d9e-a830-770ef1dbdd37/index.html#r27c1) | [T1PKS](https://docs.antismash.secondarymetabolites.org/glossary/#t1pks) | 44687 | 112335 |  |  |
| [Region 27.2](https://fungismash.secondarymetabolites.org/upload/fungi-d2dcf881-ff87-4d9e-a830-770ef1dbdd37/index.html#r27c2) | [fungal-RiPP](https://docs.antismash.secondarymetabolites.org/glossary/#fungal-ripp) | 161657 | 229072 |  |  |
| [Region 33.1](https://fungismash.secondarymetabolites.org/upload/fungi-d2dcf881-ff87-4d9e-a830-770ef1dbdd37/index.html#r33c1) | [indole](https://docs.antismash.secondarymetabolites.org/glossary/#indole) | 52431 | 83994 |  |  |
| [Region 36.1](https://fungismash.secondarymetabolites.org/upload/fungi-d2dcf881-ff87-4d9e-a830-770ef1dbdd37/index.html#r36c1) | [terpene](https://docs.antismash.secondarymetabolites.org/glossary/#terpene) | 31811 | 62891 |  |  |
| [Region 36.2](https://fungismash.secondarymetabolites.org/upload/fungi-d2dcf881-ff87-4d9e-a830-770ef1dbdd37/index.html#r36c2) | [NRPS-like](https://docs.antismash.secondarymetabolites.org/glossary/#nrps-like) | 234550 | 297968 |  |  |
| [Region 39.1](https://fungismash.secondarymetabolites.org/upload/fungi-d2dcf881-ff87-4d9e-a830-770ef1dbdd37/index.html#r39c1) | [NI-siderophore](https://docs.antismash.secondarymetabolites.org/glossary/#ni-siderophore) | 126794 | 171007 |  |  |
| [Region 40.1](https://fungismash.secondarymetabolites.org/upload/fungi-d2dcf881-ff87-4d9e-a830-770ef1dbdd37/index.html#r40c1) | [NRPS](https://docs.antismash.secondarymetabolites.org/glossary/#nrps) | 90307 | 164508 |  |  |
| [Region 44.1](https://fungismash.secondarymetabolites.org/upload/fungi-d2dcf881-ff87-4d9e-a830-770ef1dbdd37/index.html#r44c1) | [NRPS-like](https://docs.antismash.secondarymetabolites.org/glossary/#nrps-like) | 38996 | 102453 |  |  |
| [Region 44.2](https://fungismash.secondarymetabolites.org/upload/fungi-d2dcf881-ff87-4d9e-a830-770ef1dbdd37/index.html#r44c2) | [T1PKS](https://docs.antismash.secondarymetabolites.org/glossary/#t1pks) | 237528 | 305454 |  |  |
| [Region 45.1](https://fungismash.secondarymetabolites.org/upload/fungi-d2dcf881-ff87-4d9e-a830-770ef1dbdd37/index.html#r45c1) | nitropropanoic_acid,NRPS-like | 42586 | 117692 |  |  |
| [Region 49.1](https://fungismash.secondarymetabolites.org/upload/fungi-d2dcf881-ff87-4d9e-a830-770ef1dbdd37/index.html#r49c1) | T1PKS,NRPS-like | 160059 | 232803 |  |  |
| [Region 53.1](https://fungismash.secondarymetabolites.org/upload/fungi-d2dcf881-ff87-4d9e-a830-770ef1dbdd37/index.html#r53c1) | [terpene](https://docs.antismash.secondarymetabolites.org/glossary/#terpene) | 138771 | 170337 |  |  |
| [Region 54.1](https://fungismash.secondarymetabolites.org/upload/fungi-d2dcf881-ff87-4d9e-a830-770ef1dbdd37/index.html#r54c1) | [terpene](https://docs.antismash.secondarymetabolites.org/glossary/#terpene) | 18050 | 52595 |  |  |
| [Region 56.1](https://fungismash.secondarymetabolites.org/upload/fungi-d2dcf881-ff87-4d9e-a830-770ef1dbdd37/index.html#r56c1) | [terpene](https://docs.antismash.secondarymetabolites.org/glossary/#terpene) | 13110 | 44754 | Low | [squalestatin S1](https://mibig.secondarymetabolites.org/go/BGC0001839.3) |
| [Region 56.2](https://fungismash.secondarymetabolites.org/upload/fungi-d2dcf881-ff87-4d9e-a830-770ef1dbdd37/index.html#r56c2) | NRPS,T1PKS,NRPS-like | 78576 | 157100 |  |  |
| [Region 64.1](https://fungismash.secondarymetabolites.org/upload/fungi-d2dcf881-ff87-4d9e-a830-770ef1dbdd37/index.html#r64c1) | [NRPS-like](https://docs.antismash.secondarymetabolites.org/glossary/#nrps-like) | 10140 | 74113 | High | [choline](https://mibig.secondarymetabolites.org/go/BGC0002276.2) |
| [Region 69.1](https://fungismash.secondarymetabolites.org/upload/fungi-d2dcf881-ff87-4d9e-a830-770ef1dbdd37/index.html#r69c1) | [T1PKS](https://docs.antismash.secondarymetabolites.org/glossary/#t1pks) | 1 | 67230 |  |  |
| [Region 70.1](https://fungismash.secondarymetabolites.org/upload/fungi-d2dcf881-ff87-4d9e-a830-770ef1dbdd37/index.html#r70c1) | [T1PKS](https://docs.antismash.secondarymetabolites.org/glossary/#t1pks) | 72256 | 123273 |  |  |

Tab. S5 Analysis results of secondary metabolic biosynthesis gene clusters of *Gnomoniopsis quercus*

| **Region** | **Type** | **From** | **To** | **Similarity Confidence** | **Most similar known cluster** |
| --- | --- | --- | --- | --- | --- |
| [Region 1.1](https://fungismash.secondarymetabolites.org/upload/fungi-b676eb42-ec66-4d94-abaf-69ea6a447921/index.html#r1c1) | T1PKS,indole | 862810 | 933284 |  |  |
| [Region 2.1](https://fungismash.secondarymetabolites.org/upload/fungi-b676eb42-ec66-4d94-abaf-69ea6a447921/index.html#r2c1) | [NRPS-like](https://docs.antismash.secondarymetabolites.org/glossary/#nrps-like) | 969906 | 1033843 | High | [choline](https://mibig.secondarymetabolites.org/go/BGC0002276.2) |
| [Region 3.1](https://fungismash.secondarymetabolites.org/upload/fungi-b676eb42-ec66-4d94-abaf-69ea6a447921/index.html#r3c1) | [terpene-precursor](https://docs.antismash.secondarymetabolites.org/glossary/#terpene-precursor) | 443664 | 474892 |  |  |
| [Region 4.1](https://fungismash.secondarymetabolites.org/upload/fungi-b676eb42-ec66-4d94-abaf-69ea6a447921/index.html#r4c1) | [NI-siderophore](https://docs.antismash.secondarymetabolites.org/glossary/#ni-siderophore) | 404967 | 449077 |  |  |
| [Region 7.1](https://fungismash.secondarymetabolites.org/upload/fungi-b676eb42-ec66-4d94-abaf-69ea6a447921/index.html#r7c1) | NRPS-like,T1PKS | 97288 | 170090 |  |  |
| [Region 9.1](https://fungismash.secondarymetabolites.org/upload/fungi-b676eb42-ec66-4d94-abaf-69ea6a447921/index.html#r9c1) | [T1PKS](https://docs.antismash.secondarymetabolites.org/glossary/#t1pks) | 501088 | 565341 |  |  |
| [Region 11.1](https://fungismash.secondarymetabolites.org/upload/fungi-b676eb42-ec66-4d94-abaf-69ea6a447921/index.html#r11c1) | [T1PKS](https://docs.antismash.secondarymetabolites.org/glossary/#t1pks) | 125827 | 195273 |  |  |
| [Region 11.2](https://fungismash.secondarymetabolites.org/upload/fungi-b676eb42-ec66-4d94-abaf-69ea6a447921/index.html#r11c2) | [terpene](https://docs.antismash.secondarymetabolites.org/glossary/#terpene) | 432910 | 465339 | High | [clavaric acid](https://mibig.secondarymetabolites.org/go/BGC0001248.3) |
| [Region 12.1](https://fungismash.secondarymetabolites.org/upload/fungi-b676eb42-ec66-4d94-abaf-69ea6a447921/index.html#r12c1) | T1PKS,T3PKS | 262335 | 332659 |  |  |
| [Region 15.1](https://fungismash.secondarymetabolites.org/upload/fungi-b676eb42-ec66-4d94-abaf-69ea6a447921/index.html#r15c1) | [phosphonate](https://docs.antismash.secondarymetabolites.org/glossary/#phosphonate) | 227443 | 243846 |  |  |
| [Region 15.2](https://fungismash.secondarymetabolites.org/upload/fungi-b676eb42-ec66-4d94-abaf-69ea6a447921/index.html#r15c2) | [NRPS](https://docs.antismash.secondarymetabolites.org/glossary/#nrps) | 271136 | 342528 |  |  |
| [Region 15.3](https://fungismash.secondarymetabolites.org/upload/fungi-b676eb42-ec66-4d94-abaf-69ea6a447921/index.html#r15c3) | [NRPS](https://docs.antismash.secondarymetabolites.org/glossary/#nrps) | 408482 | 451479 |  |  |
| [Region 19.1](https://fungismash.secondarymetabolites.org/upload/fungi-b676eb42-ec66-4d94-abaf-69ea6a447921/index.html#r19c1) | [T1PKS](https://docs.antismash.secondarymetabolites.org/glossary/#t1pks) | 358575 | 417366 |  |  |
| [Region 23.1](https://fungismash.secondarymetabolites.org/upload/fungi-b676eb42-ec66-4d94-abaf-69ea6a447921/index.html#r23c1) | [NRPS](https://docs.antismash.secondarymetabolites.org/glossary/#nrps) | 181456 | 245368 |  |  |
| [Region 24.1](https://fungismash.secondarymetabolites.org/upload/fungi-b676eb42-ec66-4d94-abaf-69ea6a447921/index.html#r24c1) | [NRPS-like](https://docs.antismash.secondarymetabolites.org/glossary/#nrps-like) | 145619 | 209148 |  |  |
| [Region 25.1](https://fungismash.secondarymetabolites.org/upload/fungi-b676eb42-ec66-4d94-abaf-69ea6a447921/index.html#r25c1) | [T1PKS](https://docs.antismash.secondarymetabolites.org/glossary/#t1pks) | 8261 | 77452 |  |  |
| [Region 26.1](https://fungismash.secondarymetabolites.org/upload/fungi-b676eb42-ec66-4d94-abaf-69ea6a447921/index.html#r26c1) | T1PKS,terpene,indole | 102921 | 173451 |  |  |
| [Region 29.1](https://fungismash.secondarymetabolites.org/upload/fungi-b676eb42-ec66-4d94-abaf-69ea6a447921/index.html#r29c1) | [terpene](https://docs.antismash.secondarymetabolites.org/glossary/#terpene) | 2758 | 33955 | Low | [Fumagillin](https://mibig.secondarymetabolites.org/go/BGC0001067.5) |
| [Region 31.1](https://fungismash.secondarymetabolites.org/upload/fungi-b676eb42-ec66-4d94-abaf-69ea6a447921/index.html#r31c1) | terpene,T1PKS | 37517 | 132465 | Low | [HEx-tc1 terpene](https://mibig.secondarymetabolites.org/go/BGC0002216.3) |
| [Region 32.1](https://fungismash.secondarymetabolites.org/upload/fungi-b676eb42-ec66-4d94-abaf-69ea6a447921/index.html#r32c1) | NRPS,T1PKS,NRPS-like | 1 | 91909 |  |  |
| [Region 32.2](https://fungismash.secondarymetabolites.org/upload/fungi-b676eb42-ec66-4d94-abaf-69ea6a447921/index.html#r32c2) | [NRPS](https://docs.antismash.secondarymetabolites.org/glossary/#nrps) | 254672 | 297466 |  |  |
| [Region 33.1](https://fungismash.secondarymetabolites.org/upload/fungi-b676eb42-ec66-4d94-abaf-69ea6a447921/index.html#r33c1) | [T1PKS](https://docs.antismash.secondarymetabolites.org/glossary/#t1pks) | 43899 | 111908 | High | [alternapyrone](https://mibig.secondarymetabolites.org/go/BGC0000012.3) |
| [Region 33.2](https://fungismash.secondarymetabolites.org/upload/fungi-b676eb42-ec66-4d94-abaf-69ea6a447921/index.html#r33c2) | [NRPS](https://docs.antismash.secondarymetabolites.org/glossary/#nrps) | 200285 | 274489 |  |  |
| [Region 42.1](https://fungismash.secondarymetabolites.org/upload/fungi-b676eb42-ec66-4d94-abaf-69ea6a447921/index.html#r42c1) | [T1PKS](https://docs.antismash.secondarymetabolites.org/glossary/#t1pks) | 109936 | 191920 | Low | [zearalenone](https://mibig.secondarymetabolites.org/go/BGC0001057.4) |
| [Region 48.1](https://fungismash.secondarymetabolites.org/upload/fungi-b676eb42-ec66-4d94-abaf-69ea6a447921/index.html#r48c1) | [terpene](https://docs.antismash.secondarymetabolites.org/glossary/#terpene) | 77297 | 108265 |  |  |
| [Region 48.2](https://fungismash.secondarymetabolites.org/upload/fungi-b676eb42-ec66-4d94-abaf-69ea6a447921/index.html#r48c2) | [T1PKS](https://docs.antismash.secondarymetabolites.org/glossary/#t1pks) | 214543 | 259973 |  |  |
| [Region 52.1](https://fungismash.secondarymetabolites.org/upload/fungi-b676eb42-ec66-4d94-abaf-69ea6a447921/index.html#r52c1) | [NRPS-like](https://docs.antismash.secondarymetabolites.org/glossary/#nrps-like) | 179258 | 242587 |  |  |
| [Region 53.1](https://fungismash.secondarymetabolites.org/upload/fungi-b676eb42-ec66-4d94-abaf-69ea6a447921/index.html#r53c1) | [NRPS](https://docs.antismash.secondarymetabolites.org/glossary/#nrps) | 65330 | 135302 |  |  |
| [Region 53.2](https://fungismash.secondarymetabolites.org/upload/fungi-b676eb42-ec66-4d94-abaf-69ea6a447921/index.html#r53c2) | [terpene](https://docs.antismash.secondarymetabolites.org/glossary/#terpene) | 154999 | 186227 |  |  |
| [Region 55.1](https://fungismash.secondarymetabolites.org/upload/fungi-b676eb42-ec66-4d94-abaf-69ea6a447921/index.html#r55c1) | [isocyanide](https://docs.antismash.secondarymetabolites.org/glossary/#isocyanide) | 171095 | 233314 |  |  |
| [Region 59.1](https://fungismash.secondarymetabolites.org/upload/fungi-b676eb42-ec66-4d94-abaf-69ea6a447921/index.html#r59c1) | [NRPS-like](https://docs.antismash.secondarymetabolites.org/glossary/#nrps-like) | 114341 | 177846 |  |  |
| [Region 60.1](https://fungismash.secondarymetabolites.org/upload/fungi-b676eb42-ec66-4d94-abaf-69ea6a447921/index.html#r60c1) | [terpene](https://docs.antismash.secondarymetabolites.org/glossary/#terpene) | 19012 | 50437 |  |  |
| [Region 61.1](https://fungismash.secondarymetabolites.org/upload/fungi-b676eb42-ec66-4d94-abaf-69ea6a447921/index.html#r61c1) | [T1PKS](https://docs.antismash.secondarymetabolites.org/glossary/#t1pks) | 153592 | 222959 |  |  |
| [Region 63.1](https://fungismash.secondarymetabolites.org/upload/fungi-b676eb42-ec66-4d94-abaf-69ea6a447921/index.html#r63c1) | [NRPS](https://docs.antismash.secondarymetabolites.org/glossary/#nrps) | 1 | 46220 |  |  |
| [Region 63.2](https://fungismash.secondarymetabolites.org/upload/fungi-b676eb42-ec66-4d94-abaf-69ea6a447921/index.html#r63c2) | [T3PKS](https://docs.antismash.secondarymetabolites.org/glossary/#t3pks) | 130648 | 192193 |  |  |
| [Region 64.1](https://fungismash.secondarymetabolites.org/upload/fungi-b676eb42-ec66-4d94-abaf-69ea6a447921/index.html#r64c1) | [terpene](https://docs.antismash.secondarymetabolites.org/glossary/#terpene) | 112435 | 145434 |  |  |
| [Region 65.1](https://fungismash.secondarymetabolites.org/upload/fungi-b676eb42-ec66-4d94-abaf-69ea6a447921/index.html#r65c1) | [NRPS-like](https://docs.antismash.secondarymetabolites.org/glossary/#nrps-like) | 1 | 53974 |  |  |
| [Region 65.2](https://fungismash.secondarymetabolites.org/upload/fungi-b676eb42-ec66-4d94-abaf-69ea6a447921/index.html#r65c2) | T1PKS,NRPS-like | 108532 | 205009 | Low | [hypothemycin](https://mibig.secondarymetabolites.org/go/BGC0000077.3) |
| [Region 67.1](https://fungismash.secondarymetabolites.org/upload/fungi-b676eb42-ec66-4d94-abaf-69ea6a447921/index.html#r67c1) | [T1PKS](https://docs.antismash.secondarymetabolites.org/glossary/#t1pks) | 20427 | 88251 | Low | [emodin/monodictyphenone/shamixanthone](https://mibig.secondarymetabolites.org/go/BGC0000101.5) |

Tab. S5 Analysis results of secondary metabolic biosynthesis gene clusters of *Gnomoniopsis quercus* (Continued)

| [Region 75.1](https://fungismash.secondarymetabolites.org/upload/fungi-b676eb42-ec66-4d94-abaf-69ea6a447921/index.html#r75c1) | terpene,T1PKS | 132984 | 190232 | Medium | [HEx-tc1 terpene](https://mibig.secondarymetabolites.org/go/BGC0002216.3) |
| --- | --- | --- | --- | --- | --- |
| [Region 76.1](https://fungismash.secondarymetabolites.org/upload/fungi-b676eb42-ec66-4d94-abaf-69ea6a447921/index.html#r76c1) | [NRPS-like](https://docs.antismash.secondarymetabolites.org/glossary/#nrps-like) | 76408 | 140540 |  |  |
| [Region 77.1](https://fungismash.secondarymetabolites.org/upload/fungi-b676eb42-ec66-4d94-abaf-69ea6a447921/index.html#r77c1) | NRPS-like,T1PKS | 124313 | 189349 |  |  |
| [Region 81.1](https://fungismash.secondarymetabolites.org/upload/fungi-b676eb42-ec66-4d94-abaf-69ea6a447921/index.html#r81c1) | [NRPS-like](https://docs.antismash.secondarymetabolites.org/glossary/#nrps-like) | 121950 | 180889 |  |  |
| [Region 82.1](https://fungismash.secondarymetabolites.org/upload/fungi-b676eb42-ec66-4d94-abaf-69ea6a447921/index.html#r82c1) | [terpene](https://docs.antismash.secondarymetabolites.org/glossary/#terpene) | 78311 | 109848 |  |  |
| [Region 82.2](https://fungismash.secondarymetabolites.org/upload/fungi-b676eb42-ec66-4d94-abaf-69ea6a447921/index.html#r82c2) | [NRPS-like](https://docs.antismash.secondarymetabolites.org/glossary/#nrps-like) | 140955 | 180849 |  |  |
| [Region 87.1](https://fungismash.secondarymetabolites.org/upload/fungi-b676eb42-ec66-4d94-abaf-69ea6a447921/index.html#r87c1) | [T1PKS](https://docs.antismash.secondarymetabolites.org/glossary/#t1pks) | 25113 | 95578 |  |  |
| [Region 88.1](https://fungismash.secondarymetabolites.org/upload/fungi-b676eb42-ec66-4d94-abaf-69ea6a447921/index.html#r88c1) | [terpene](https://docs.antismash.secondarymetabolites.org/glossary/#terpene) | 149614 | 172058 |  |  |
| [Region 89.1](https://fungismash.secondarymetabolites.org/upload/fungi-b676eb42-ec66-4d94-abaf-69ea6a447921/index.html#r89c1) | [terpene-precursor](https://docs.antismash.secondarymetabolites.org/glossary/#terpene-precursor) | 18757 | 50028 |  |  |
| [Region 90.1](https://fungismash.secondarymetabolites.org/upload/fungi-b676eb42-ec66-4d94-abaf-69ea6a447921/index.html#r90c1) | [NRPS-like](https://docs.antismash.secondarymetabolites.org/glossary/#nrps-like) | 56639 | 119901 |  |  |
| [Region 97.1](https://fungismash.secondarymetabolites.org/upload/fungi-b676eb42-ec66-4d94-abaf-69ea6a447921/index.html#r97c1) | [terpene](https://docs.antismash.secondarymetabolites.org/glossary/#terpene) | 102442 | 134540 |  |  |
| [Region 104.1](https://fungismash.secondarymetabolites.org/upload/fungi-b676eb42-ec66-4d94-abaf-69ea6a447921/index.html#r104c1) | [terpene](https://docs.antismash.secondarymetabolites.org/glossary/#terpene) | 65099 | 97065 |  |  |
| [Region 115.1](https://fungismash.secondarymetabolites.org/upload/fungi-b676eb42-ec66-4d94-abaf-69ea6a447921/index.html#r115c1) | [terpene](https://docs.antismash.secondarymetabolites.org/glossary/#terpene) | 85220 | 116792 |  |  |
| [Region 116.1](https://fungismash.secondarymetabolites.org/upload/fungi-b676eb42-ec66-4d94-abaf-69ea6a447921/index.html#r116c1) | [other](https://docs.antismash.secondarymetabolites.org/glossary/#other) | 9111 | 70025 |  |  |
| [Region 117.1](https://fungismash.secondarymetabolites.org/upload/fungi-b676eb42-ec66-4d94-abaf-69ea6a447921/index.html#r117c1) | [T1PKS](https://docs.antismash.secondarymetabolites.org/glossary/#t1pks) | 6269 | 75799 |  |  |
| [Region 121.1](https://fungismash.secondarymetabolites.org/upload/fungi-b676eb42-ec66-4d94-abaf-69ea6a447921/index.html#r121c1) | T1PKS,indole | 1 | 104530 | Low | [flavoglaucin](https://mibig.secondarymetabolites.org/go/BGC0002234.3) |
| [Region 133.1](https://fungismash.secondarymetabolites.org/upload/fungi-b676eb42-ec66-4d94-abaf-69ea6a447921/index.html#r133c1) | [T1PKS](https://docs.antismash.secondarymetabolites.org/glossary/#t1pks) | 57191 | 110469 | Medium | [scytalone/T3HN](https://mibig.secondarymetabolites.org/go/BGC0002161.2) |
| [Region 136.1](https://fungismash.secondarymetabolites.org/upload/fungi-b676eb42-ec66-4d94-abaf-69ea6a447921/index.html#r136c1) | [NRPS-like](https://docs.antismash.secondarymetabolites.org/glossary/#nrps-like) | 1 | 46775 |  |  |
| [Region 149.1](https://fungismash.secondarymetabolites.org/upload/fungi-b676eb42-ec66-4d94-abaf-69ea6a447921/index.html#r149c1) | [terpene](https://docs.antismash.secondarymetabolites.org/glossary/#terpene) | 1 | 30147 |  |  |
| [Region 151.1](https://fungismash.secondarymetabolites.org/upload/fungi-b676eb42-ec66-4d94-abaf-69ea6a447921/index.html#r151c1) | [NRPS-like](https://docs.antismash.secondarymetabolites.org/glossary/#nrps-like) | 29997 | 89910 |  |  |
| [Region 154.1](https://fungismash.secondarymetabolites.org/upload/fungi-b676eb42-ec66-4d94-abaf-69ea6a447921/index.html#r154c1) | [T1PKS](https://docs.antismash.secondarymetabolites.org/glossary/#t1pks) | 37482 | 86957 |  |  |
| [Region 188.1](https://fungismash.secondarymetabolites.org/upload/fungi-b676eb42-ec66-4d94-abaf-69ea6a447921/index.html#r188c1) | [terpene](https://docs.antismash.secondarymetabolites.org/glossary/#terpene) | 18818 | 50448 | Low | [squalestatin S1](https://mibig.secondarymetabolites.org/go/BGC0001839.3) |
| [Region 202.1](https://fungismash.secondarymetabolites.org/upload/fungi-b676eb42-ec66-4d94-abaf-69ea6a447921/index.html#r202c1) | [NRPS-like](https://docs.antismash.secondarymetabolites.org/glossary/#nrps-like) | 1 | 52801 |  |  |
| [Region 222.1](https://fungismash.secondarymetabolites.org/upload/fungi-b676eb42-ec66-4d94-abaf-69ea6a447921/index.html#r222c1) | [terpene](https://docs.antismash.secondarymetabolites.org/glossary/#terpene) | 1 | 27241 |  |  |
| [Region 230.1](https://fungismash.secondarymetabolites.org/upload/fungi-b676eb42-ec66-4d94-abaf-69ea6a447921/index.html#r230c1) | T1PKS,indole,terpene | 1 | 40556 |  |  |
| [Region 236.1](https://fungismash.secondarymetabolites.org/upload/fungi-b676eb42-ec66-4d94-abaf-69ea6a447921/index.html#r236c1) | NRPS-like,T1PKS | 1 | 36696 |  |  |
| [Region 284.1](https://fungismash.secondarymetabolites.org/upload/fungi-b676eb42-ec66-4d94-abaf-69ea6a447921/index.html#r284c1) | [NRPS-like](https://docs.antismash.secondarymetabolites.org/glossary/#nrps-like) | 1 | 17624 |  |  |
| [Region 287.1](https://fungismash.secondarymetabolites.org/upload/fungi-b676eb42-ec66-4d94-abaf-69ea6a447921/index.html#r287c1) | [terpene](https://docs.antismash.secondarymetabolites.org/glossary/#terpene) | 1 | 17041 |  |  |

Tab. S6 Analysis results of secondary metabolic biosynthesis gene clusters of *Gnomoniopsis ledongensis*

| **Region** | **Type** | **From** | **To** | **Similarity Confidence** | **Most similar known cluster** |
| --- | --- | --- | --- | --- | --- |
| [Region 1.1](https://fungismash.secondarymetabolites.org/upload/fungi-84c041ef-d570-41a9-87b2-b10a33adf869/index.html#r1c1) | [terpene-precursor](https://docs.antismash.secondarymetabolites.org/glossary/#terpene-precursor) | 742885 | 774113 |  |  |
| [Region 2.1](https://fungismash.secondarymetabolites.org/upload/fungi-84c041ef-d570-41a9-87b2-b10a33adf869/index.html#r2c1) | T1PKS,indole | 147782 | 217847 |  |  |
| [Region 2.2](https://fungismash.secondarymetabolites.org/upload/fungi-84c041ef-d570-41a9-87b2-b10a33adf869/index.html#r2c2) | [terpene](https://docs.antismash.secondarymetabolites.org/glossary/#terpene) | 244377 | 275557 |  |  |
| [Region 5.1](https://fungismash.secondarymetabolites.org/upload/fungi-84c041ef-d570-41a9-87b2-b10a33adf869/index.html#r5c1) | [terpene](https://docs.antismash.secondarymetabolites.org/glossary/#terpene) | 237013 | 268137 |  |  |
| [Region 5.2](https://fungismash.secondarymetabolites.org/upload/fungi-84c041ef-d570-41a9-87b2-b10a33adf869/index.html#r5c2) | [NRPS-like](https://docs.antismash.secondarymetabolites.org/glossary/#nrps-like) | 356696 | 420054 |  |  |
| [Region 9.1](https://fungismash.secondarymetabolites.org/upload/fungi-84c041ef-d570-41a9-87b2-b10a33adf869/index.html#r9c1) | [NRPS-like](https://docs.antismash.secondarymetabolites.org/glossary/#nrps-like) | 272798 | 336343 |  |  |
| [Region 10.1](https://fungismash.secondarymetabolites.org/upload/fungi-84c041ef-d570-41a9-87b2-b10a33adf869/index.html#r10c1) | terpene,T1PKS | 664333 | 727957 | Low | [Fumagillin](https://mibig.secondarymetabolites.org/go/BGC0001067.5) |
| [Region 11.1](https://fungismash.secondarymetabolites.org/upload/fungi-84c041ef-d570-41a9-87b2-b10a33adf869/index.html#r11c1) | [NRPS](https://docs.antismash.secondarymetabolites.org/glossary/#nrps) | 142907 | 213487 |  |  |
| [Region 11.2](https://fungismash.secondarymetabolites.org/upload/fungi-84c041ef-d570-41a9-87b2-b10a33adf869/index.html#r11c2) | [T1PKS](https://docs.antismash.secondarymetabolites.org/glossary/#t1pks) | 466540 | 567200 | Low | [azasperpyranone A/azasperpyranone B/azasperpyranone C/azasperpyranone D/azasperpyranone E/azasperpyranone F/azasperpyranone G/azasperpyranone H](https://mibig.secondarymetabolites.org/go/BGC0002267.2) |
| [Region 14.1](https://fungismash.secondarymetabolites.org/upload/fungi-84c041ef-d570-41a9-87b2-b10a33adf869/index.html#r14c1) | NRPS,indole | 1 | 54263 |  |  |
| [Region 17.1](https://fungismash.secondarymetabolites.org/upload/fungi-84c041ef-d570-41a9-87b2-b10a33adf869/index.html#r17c1) | [NRPS-like](https://docs.antismash.secondarymetabolites.org/glossary/#nrps-like) | 365995 | 429266 |  |  |
| [Region 18.1](https://fungismash.secondarymetabolites.org/upload/fungi-84c041ef-d570-41a9-87b2-b10a33adf869/index.html#r18c1) | [T1PKS](https://docs.antismash.secondarymetabolites.org/glossary/#t1pks) | 419082 | 486847 |  |  |
| [Region 19.1](https://fungismash.secondarymetabolites.org/upload/fungi-84c041ef-d570-41a9-87b2-b10a33adf869/index.html#r19c1) | NRPS-like,T1PKS | 269497 | 352167 |  |  |
| [Region 22.1](https://fungismash.secondarymetabolites.org/upload/fungi-84c041ef-d570-41a9-87b2-b10a33adf869/index.html#r22c1) | NRPS-like,T1PKS | 16048 | 118876 | Low | [hypothemycin](https://mibig.secondarymetabolites.org/go/BGC0000077.3) |
| [Region 22.2](https://fungismash.secondarymetabolites.org/upload/fungi-84c041ef-d570-41a9-87b2-b10a33adf869/index.html#r22c2) | [NRPS-like](https://docs.antismash.secondarymetabolites.org/glossary/#nrps-like) | 165942 | 229221 |  |  |
| [Region 24.1](https://fungismash.secondarymetabolites.org/upload/fungi-84c041ef-d570-41a9-87b2-b10a33adf869/index.html#r24c1) | [phosphonate](https://docs.antismash.secondarymetabolites.org/glossary/#phosphonate) | 46669 | 63073 |  |  |
| [Region 28.1](https://fungismash.secondarymetabolites.org/upload/fungi-84c041ef-d570-41a9-87b2-b10a33adf869/index.html#r28c1) | [isocyanide](https://docs.antismash.secondarymetabolites.org/glossary/#isocyanide) | 251140 | 313411 |  |  |
| [Region 30.1](https://fungismash.secondarymetabolites.org/upload/fungi-84c041ef-d570-41a9-87b2-b10a33adf869/index.html#r30c1) | [terpene](https://docs.antismash.secondarymetabolites.org/glossary/#terpene) | 89694 | 125069 |  |  |
| [Region 31.1](https://fungismash.secondarymetabolites.org/upload/fungi-84c041ef-d570-41a9-87b2-b10a33adf869/index.html#r31c1) | [T1PKS](https://docs.antismash.secondarymetabolites.org/glossary/#t1pks) | 300004 | 366864 |  |  |
| [Region 32.1](https://fungismash.secondarymetabolites.org/upload/fungi-84c041ef-d570-41a9-87b2-b10a33adf869/index.html#r32c1) | [NRPS](https://docs.antismash.secondarymetabolites.org/glossary/#nrps) | 63929 | 138146 |  |  |
| [Region 32.2](https://fungismash.secondarymetabolites.org/upload/fungi-84c041ef-d570-41a9-87b2-b10a33adf869/index.html#r32c2) | [T1PKS](https://docs.antismash.secondarymetabolites.org/glossary/#t1pks) | 220047 | 288123 | High | [alternapyrone](https://mibig.secondarymetabolites.org/go/BGC0000012.3) |
| [Region 34.1](https://fungismash.secondarymetabolites.org/upload/fungi-84c041ef-d570-41a9-87b2-b10a33adf869/index.html#r34c1) | NRPS-like,T1PKS | 33178 | 106014 |  |  |
| [Region 34.2](https://fungismash.secondarymetabolites.org/upload/fungi-84c041ef-d570-41a9-87b2-b10a33adf869/index.html#r34c2) | [T1PKS](https://docs.antismash.secondarymetabolites.org/glossary/#t1pks) | 301433 | 369125 | Low | [depudecin](https://mibig.secondarymetabolites.org/go/BGC0000046.4) |
| [Region 35.1](https://fungismash.secondarymetabolites.org/upload/fungi-84c041ef-d570-41a9-87b2-b10a33adf869/index.html#r35c1) | [terpene](https://docs.antismash.secondarymetabolites.org/glossary/#terpene) | 184466 | 215768 |  |  |
| [Region 35.2](https://fungismash.secondarymetabolites.org/upload/fungi-84c041ef-d570-41a9-87b2-b10a33adf869/index.html#r35c2) | [NRPS](https://docs.antismash.secondarymetabolites.org/glossary/#nrps) | 221382 | 291354 |  |  |
| [Region 37.1](https://fungismash.secondarymetabolites.org/upload/fungi-84c041ef-d570-41a9-87b2-b10a33adf869/index.html#r37c1) | [T1PKS](https://docs.antismash.secondarymetabolites.org/glossary/#t1pks) | 264050 | 332219 |  |  |
| [Region 43.1](https://fungismash.secondarymetabolites.org/upload/fungi-84c041ef-d570-41a9-87b2-b10a33adf869/index.html#r43c1) | [T3PKS](https://docs.antismash.secondarymetabolites.org/glossary/#t3pks) | 61346 | 122903 |  |  |
| [Region 43.2](https://fungismash.secondarymetabolites.org/upload/fungi-84c041ef-d570-41a9-87b2-b10a33adf869/index.html#r43c2) | NRP-metallophore,NRPS | 201302 | 279876 |  |  |
| [Region 45.1](https://fungismash.secondarymetabolites.org/upload/fungi-84c041ef-d570-41a9-87b2-b10a33adf869/index.html#r45c1) | [NRPS-like](https://docs.antismash.secondarymetabolites.org/glossary/#nrps-like) | 219107 | 283057 | High | [choline](https://mibig.secondarymetabolites.org/go/BGC0002276.2) |
| [Region 48.1](https://fungismash.secondarymetabolites.org/upload/fungi-84c041ef-d570-41a9-87b2-b10a33adf869/index.html#r48c1) | [NI-siderophore](https://docs.antismash.secondarymetabolites.org/glossary/#ni-siderophore) | 247711 | 291869 |  |  |
| [Region 51.1](https://fungismash.secondarymetabolites.org/upload/fungi-84c041ef-d570-41a9-87b2-b10a33adf869/index.html#r51c1) | [T1PKS](https://docs.antismash.secondarymetabolites.org/glossary/#t1pks) | 119030 | 188992 | Low | [gregatin A](https://mibig.secondarymetabolites.org/go/BGC0002185.2) |
| [Region 55.1](https://fungismash.secondarymetabolites.org/upload/fungi-84c041ef-d570-41a9-87b2-b10a33adf869/index.html#r55c1) | T1PKS,indole | 1 | 57548 |  |  |
| [Region 56.1](https://fungismash.secondarymetabolites.org/upload/fungi-84c041ef-d570-41a9-87b2-b10a33adf869/index.html#r56c1) | [NRPS](https://docs.antismash.secondarymetabolites.org/glossary/#nrps) | 26352 | 98593 |  |  |

Tab. S6 Analysis results of secondary metabolic biosynthesis gene clusters of *Gnomoniopsis ledongensis*	(Continued)

| **Region** | **Type** | **From** | **To** | **Similarity Confidence** | **Most similar known cluster** |
| --- | --- | --- | --- | --- | --- |
| [Region 56.2](https://fungismash.secondarymetabolites.org/upload/fungi-84c041ef-d570-41a9-87b2-b10a33adf869/index.html#r56c2) | [terpene](https://docs.antismash.secondarymetabolites.org/glossary/#terpene) | 257944 | 279845 |  |  |
| [Region 62.1](https://fungismash.secondarymetabolites.org/upload/fungi-84c041ef-d570-41a9-87b2-b10a33adf869/index.html#r62c1) | [T1PKS](https://docs.antismash.secondarymetabolites.org/glossary/#t1pks) | 18443 | 87786 |  |  |
| [Region 67.1](https://fungismash.secondarymetabolites.org/upload/fungi-84c041ef-d570-41a9-87b2-b10a33adf869/index.html#r67c1) | NRPS-like,T1PKS | 122625 | 187875 |  |  |
| [Region 69.1](https://fungismash.secondarymetabolites.org/upload/fungi-84c041ef-d570-41a9-87b2-b10a33adf869/index.html#r69c1) | [T1PKS](https://docs.antismash.secondarymetabolites.org/glossary/#t1pks) | 1 | 56038 | Medium | [scytalone/T3HN](https://mibig.secondarymetabolites.org/go/BGC0002161.2) |
| [Region 70.1](https://fungismash.secondarymetabolites.org/upload/fungi-84c041ef-d570-41a9-87b2-b10a33adf869/index.html#r70c1) | [terpene](https://docs.antismash.secondarymetabolites.org/glossary/#terpene) | 25566 | 57198 |  |  |
| [Region 72.1](https://fungismash.secondarymetabolites.org/upload/fungi-84c041ef-d570-41a9-87b2-b10a33adf869/index.html#r72c1) | [T1PKS](https://docs.antismash.secondarymetabolites.org/glossary/#t1pks) | 148476 | 184308 |  |  |
| [Region 73.1](https://fungismash.secondarymetabolites.org/upload/fungi-84c041ef-d570-41a9-87b2-b10a33adf869/index.html#r73c1) | [terpene](https://docs.antismash.secondarymetabolites.org/glossary/#terpene) | 43641 | 79659 |  |  |
| [Region 76.1](https://fungismash.secondarymetabolites.org/upload/fungi-84c041ef-d570-41a9-87b2-b10a33adf869/index.html#r76c1) | [NRPS-like](https://docs.antismash.secondarymetabolites.org/glossary/#nrps-like) | 40205 | 103527 |  |  |
| [Region 77.1](https://fungismash.secondarymetabolites.org/upload/fungi-84c041ef-d570-41a9-87b2-b10a33adf869/index.html#r77c1) | [NRPS-like](https://docs.antismash.secondarymetabolites.org/glossary/#nrps-like) | 6477 | 71644 |  |  |
| [Region 77.2](https://fungismash.secondarymetabolites.org/upload/fungi-84c041ef-d570-41a9-87b2-b10a33adf869/index.html#r77c2) | [terpene](https://docs.antismash.secondarymetabolites.org/glossary/#terpene) | 91787 | 124240 | High | [clavaric acid](https://mibig.secondarymetabolites.org/go/BGC0001248.3) |
| [Region 80.1](https://fungismash.secondarymetabolites.org/upload/fungi-84c041ef-d570-41a9-87b2-b10a33adf869/index.html#r80c1) | [terpene-precursor](https://docs.antismash.secondarymetabolites.org/glossary/#terpene-precursor) | 1 | 21813 |  |  |
| [Region 84.1](https://fungismash.secondarymetabolites.org/upload/fungi-84c041ef-d570-41a9-87b2-b10a33adf869/index.html#r84c1) | [NRPS](https://docs.antismash.secondarymetabolites.org/glossary/#nrps) | 66072 | 129962 |  |  |
| [Region 87.1](https://fungismash.secondarymetabolites.org/upload/fungi-84c041ef-d570-41a9-87b2-b10a33adf869/index.html#r87c1) | NRPS-like,T1PKS | 1 | 51770 |  |  |
| [Region 89.1](https://fungismash.secondarymetabolites.org/upload/fungi-84c041ef-d570-41a9-87b2-b10a33adf869/index.html#r89c1) | [terpene](https://docs.antismash.secondarymetabolites.org/glossary/#terpene) | 81035 | 112126 | Medium | [HEx-tc1 terpene](https://mibig.secondarymetabolites.org/go/BGC0002216.3) |
| [Region 93.1](https://fungismash.secondarymetabolites.org/upload/fungi-84c041ef-d570-41a9-87b2-b10a33adf869/index.html#r93c1) | [T1PKS](https://docs.antismash.secondarymetabolites.org/glossary/#t1pks) | 46041 | 114128 | Low | [trichoxide](https://mibig.secondarymetabolites.org/go/BGC0002233.3) |
| [Region 108.1](https://fungismash.secondarymetabolites.org/upload/fungi-84c041ef-d570-41a9-87b2-b10a33adf869/index.html#r108c1) | [terpene](https://docs.antismash.secondarymetabolites.org/glossary/#terpene) | 13679 | 45645 |  |  |
| [Region 113.1](https://fungismash.secondarymetabolites.org/upload/fungi-84c041ef-d570-41a9-87b2-b10a33adf869/index.html#r113c1) | [NRPS-like](https://docs.antismash.secondarymetabolites.org/glossary/#nrps-like) | 1 | 62455 |  |  |
| [Region 114.1](https://fungismash.secondarymetabolites.org/upload/fungi-84c041ef-d570-41a9-87b2-b10a33adf869/index.html#r114c1) | [other](https://docs.antismash.secondarymetabolites.org/glossary/#other) | 27036 | 82807 |  |  |
| [Region 116.1](https://fungismash.secondarymetabolites.org/upload/fungi-84c041ef-d570-41a9-87b2-b10a33adf869/index.html#r116c1) | [T1PKS](https://docs.antismash.secondarymetabolites.org/glossary/#t1pks) | 1 | 51045 |  |  |
| [Region 126.1](https://fungismash.secondarymetabolites.org/upload/fungi-84c041ef-d570-41a9-87b2-b10a33adf869/index.html#r126c1) | T1PKS,NRPS | 1 | 59962 |  |  |
| [Region 129.1](https://fungismash.secondarymetabolites.org/upload/fungi-84c041ef-d570-41a9-87b2-b10a33adf869/index.html#r129c1) | [NRPS-like](https://docs.antismash.secondarymetabolites.org/glossary/#nrps-like) | 1 | 44663 |  |  |
| [Region 133.1](https://fungismash.secondarymetabolites.org/upload/fungi-84c041ef-d570-41a9-87b2-b10a33adf869/index.html#r133c1) | [terpene](https://docs.antismash.secondarymetabolites.org/glossary/#terpene) | 26965 | 52828 |  |  |
| [Region 134.1](https://fungismash.secondarymetabolites.org/upload/fungi-84c041ef-d570-41a9-87b2-b10a33adf869/index.html#r134c1) | terpene,T1PKS | 1301 | 50855 |  |  |
| [Region 148.1](https://fungismash.secondarymetabolites.org/upload/fungi-84c041ef-d570-41a9-87b2-b10a33adf869/index.html#r148c1) | [T1PKS](https://docs.antismash.secondarymetabolites.org/glossary/#t1pks) | 1 | 29965 | High | [betaenone C/probetaenone I/stemphyloxin II/compound 4/compound 6/compound 7/compound 9/compound 3/dehydroprobetaenone I/compound 5](https://mibig.secondarymetabolites.org/go/BGC0002165.3) |
| [Region 177.1](https://fungismash.secondarymetabolites.org/upload/fungi-84c041ef-d570-41a9-87b2-b10a33adf869/index.html#r177c1) | [NRPS-like](https://docs.antismash.secondarymetabolites.org/glossary/#nrps-like) | 1 | 12737 |  |  |

Tab. S7 Analysis results of secondary metabolic biosynthesis gene clusters of *Gnomoniopsis conghuaensis*

| **Region** | **Type** | **From** | **To** | **Similarity Confidence** | **Most similar known cluster** |
| --- | --- | --- | --- | --- | --- |
| [Region 1.1](https://fungismash.secondarymetabolites.org/upload/fungi-ce3691ee-6846-4a3a-89fb-9351fcfff587/index.html#r1c1) | [terpene-precursor](https://docs.antismash.secondarymetabolites.org/glossary/#terpene-precursor) | 54203 | 85398 |  |  |
| [Region 1.2](https://fungismash.secondarymetabolites.org/upload/fungi-ce3691ee-6846-4a3a-89fb-9351fcfff587/index.html#r1c2) | [NRPS-like](https://docs.antismash.secondarymetabolites.org/glossary/#nrps-like) | 457779 | 522094 |  |  |
| [Region 2.1](https://fungismash.secondarymetabolites.org/upload/fungi-ce3691ee-6846-4a3a-89fb-9351fcfff587/index.html#r2c1) | [T1PKS](https://docs.antismash.secondarymetabolites.org/glossary/#t1pks) | 10150 | 78647 |  |  |
| [Region 3.1](https://fungismash.secondarymetabolites.org/upload/fungi-ce3691ee-6846-4a3a-89fb-9351fcfff587/index.html#r3c1) | [terpene](https://docs.antismash.secondarymetabolites.org/glossary/#terpene) | 584889 | 616368 |  |  |
| [Region 4.1](https://fungismash.secondarymetabolites.org/upload/fungi-ce3691ee-6846-4a3a-89fb-9351fcfff587/index.html#r4c1) | NRPS,T1PKS,terpene | 1 | 53037 |  |  |
| [Region 6.1](https://fungismash.secondarymetabolites.org/upload/fungi-ce3691ee-6846-4a3a-89fb-9351fcfff587/index.html#r6c1) | [T1PKS](https://docs.antismash.secondarymetabolites.org/glossary/#t1pks) | 107463 | 175296 |  |  |
| [Region 7.1](https://fungismash.secondarymetabolites.org/upload/fungi-ce3691ee-6846-4a3a-89fb-9351fcfff587/index.html#r7c1) | [NRPS](https://docs.antismash.secondarymetabolites.org/glossary/#nrps) | 554460 | 624416 |  |  |
| [Region 8.1](https://fungismash.secondarymetabolites.org/upload/fungi-ce3691ee-6846-4a3a-89fb-9351fcfff587/index.html#r8c1) | NRPS-like,T1PKS | 306469 | 379018 |  |  |
| [Region 10.1](https://fungismash.secondarymetabolites.org/upload/fungi-ce3691ee-6846-4a3a-89fb-9351fcfff587/index.html#r10c1) | [T1PKS](https://docs.antismash.secondarymetabolites.org/glossary/#t1pks) | 554715 | 593423 | Low | [scytalone/T3HN](https://mibig.secondarymetabolites.org/go/BGC0002161.2) |
| [Region 12.1](https://fungismash.secondarymetabolites.org/upload/fungi-ce3691ee-6846-4a3a-89fb-9351fcfff587/index.html#r12c1) | [other](https://docs.antismash.secondarymetabolites.org/glossary/#other) | 447491 | 511480 |  |  |
| [Region 14.1](https://fungismash.secondarymetabolites.org/upload/fungi-ce3691ee-6846-4a3a-89fb-9351fcfff587/index.html#r14c1) | [nitropropanoic_acid](https://docs.antismash.secondarymetabolites.org/glossary/#nitropropanoic_acid) | 241177 | 263280 |  |  |
| [Region 15.1](https://fungismash.secondarymetabolites.org/upload/fungi-ce3691ee-6846-4a3a-89fb-9351fcfff587/index.html#r15c1) | NRP-metallophore,NRPS | 162439 | 241099 |  |  |
| [Region 15.2](https://fungismash.secondarymetabolites.org/upload/fungi-ce3691ee-6846-4a3a-89fb-9351fcfff587/index.html#r15c2) | [T3PKS](https://docs.antismash.secondarymetabolites.org/glossary/#t3pks) | 317627 | 379180 |  |  |
| [Region 16.1](https://fungismash.secondarymetabolites.org/upload/fungi-ce3691ee-6846-4a3a-89fb-9351fcfff587/index.html#r16c1) | terpene,T1PKS | 327546 | 402045 |  |  |
| [Region 20.1](https://fungismash.secondarymetabolites.org/upload/fungi-ce3691ee-6846-4a3a-89fb-9351fcfff587/index.html#r20c1) | [terpene](https://docs.antismash.secondarymetabolites.org/glossary/#terpene) | 43135 | 74688 |  |  |
| [Region 24.1](https://fungismash.secondarymetabolites.org/upload/fungi-ce3691ee-6846-4a3a-89fb-9351fcfff587/index.html#r24c1) | [T1PKS](https://docs.antismash.secondarymetabolites.org/glossary/#t1pks) | 150430 | 218297 |  |  |
| [Region 26.1](https://fungismash.secondarymetabolites.org/upload/fungi-ce3691ee-6846-4a3a-89fb-9351fcfff587/index.html#r26c1) | [NRPS](https://docs.antismash.secondarymetabolites.org/glossary/#nrps) | 97519 | 168252 |  |  |
| [Region 26.2](https://fungismash.secondarymetabolites.org/upload/fungi-ce3691ee-6846-4a3a-89fb-9351fcfff587/index.html#r26c2) | [NRPS-like](https://docs.antismash.secondarymetabolites.org/glossary/#nrps-like) | 288208 | 343348 |  |  |
| [Region 27.1](https://fungismash.secondarymetabolites.org/upload/fungi-ce3691ee-6846-4a3a-89fb-9351fcfff587/index.html#r27c1) | [NRPS-like](https://docs.antismash.secondarymetabolites.org/glossary/#nrps-like) | 202396 | 266118 |  |  |
| [Region 36.1](https://fungismash.secondarymetabolites.org/upload/fungi-ce3691ee-6846-4a3a-89fb-9351fcfff587/index.html#r36c1) | [NRPS](https://docs.antismash.secondarymetabolites.org/glossary/#nrps) | 117098 | 182789 |  |  |
| [Region 37.1](https://fungismash.secondarymetabolites.org/upload/fungi-ce3691ee-6846-4a3a-89fb-9351fcfff587/index.html#r37c1) | [T1PKS](https://docs.antismash.secondarymetabolites.org/glossary/#t1pks) | 75080 | 142687 |  |  |
| [Region 38.1](https://fungismash.secondarymetabolites.org/upload/fungi-ce3691ee-6846-4a3a-89fb-9351fcfff587/index.html#r38c1) | [T1PKS](https://docs.antismash.secondarymetabolites.org/glossary/#t1pks) | 251360 | 297118 |  |  |
| [Region 39.1](https://fungismash.secondarymetabolites.org/upload/fungi-ce3691ee-6846-4a3a-89fb-9351fcfff587/index.html#r39c1) | [T1PKS](https://docs.antismash.secondarymetabolites.org/glossary/#t1pks) | 218779 | 287341 |  |  |
| [Region 47.1](https://fungismash.secondarymetabolites.org/upload/fungi-ce3691ee-6846-4a3a-89fb-9351fcfff587/index.html#r47c1) | T1PKS,NRPS | 3647 | 102990 | Medium | [CJ-16,173/CJ-15,696/citridone B/citridone A/citridone B'](https://mibig.secondarymetabolites.org/go/BGC0002268.3) |
| [Region 49.1](https://fungismash.secondarymetabolites.org/upload/fungi-ce3691ee-6846-4a3a-89fb-9351fcfff587/index.html#r49c1) | [NRPS-like](https://docs.antismash.secondarymetabolites.org/glossary/#nrps-like) | 19238 | 83202 | High | [choline](https://mibig.secondarymetabolites.org/go/BGC0002276.2) |
| [Region 50.1](https://fungismash.secondarymetabolites.org/upload/fungi-ce3691ee-6846-4a3a-89fb-9351fcfff587/index.html#r50c1) | NRPS,T1PKS | 1 | 62423 |  |  |
| [Region 56.1](https://fungismash.secondarymetabolites.org/upload/fungi-ce3691ee-6846-4a3a-89fb-9351fcfff587/index.html#r56c1) | [NRPS](https://docs.antismash.secondarymetabolites.org/glossary/#nrps) | 52349 | 116265 |  |  |
| [Region 57.1](https://fungismash.secondarymetabolites.org/upload/fungi-ce3691ee-6846-4a3a-89fb-9351fcfff587/index.html#r57c1) | [terpene](https://docs.antismash.secondarymetabolites.org/glossary/#terpene) | 176364 | 208563 |  |  |
| [Region 58.1](https://fungismash.secondarymetabolites.org/upload/fungi-ce3691ee-6846-4a3a-89fb-9351fcfff587/index.html#r58c1) | [terpene](https://docs.antismash.secondarymetabolites.org/glossary/#terpene) | 1 | 27380 | Low | [Fumagillin](https://mibig.secondarymetabolites.org/go/BGC0001067.5) |
| [Region 64.1](https://fungismash.secondarymetabolites.org/upload/fungi-ce3691ee-6846-4a3a-89fb-9351fcfff587/index.html#r64c1) | [T1PKS](https://docs.antismash.secondarymetabolites.org/glossary/#t1pks) | 146827 | 211044 |  |  |
| [Region 84.1](https://fungismash.secondarymetabolites.org/upload/fungi-ce3691ee-6846-4a3a-89fb-9351fcfff587/index.html#r84c1) | [NRPS-like](https://docs.antismash.secondarymetabolites.org/glossary/#nrps-like) | 1 | 40218 |  |  |
| [Region 88.1](https://fungismash.secondarymetabolites.org/upload/fungi-ce3691ee-6846-4a3a-89fb-9351fcfff587/index.html#r88c1) | [terpene](https://docs.antismash.secondarymetabolites.org/glossary/#terpene) | 33638 | 66107 | High | [clavaric acid](https://mibig.secondarymetabolites.org/go/BGC0001248.3) |
| [Region 88.2](https://fungismash.secondarymetabolites.org/upload/fungi-ce3691ee-6846-4a3a-89fb-9351fcfff587/index.html#r88c2) | [NRPS-like](https://docs.antismash.secondarymetabolites.org/glossary/#nrps-like) | 103707 | 162612 |  |  |
| [Region 92.1](https://fungismash.secondarymetabolites.org/upload/fungi-ce3691ee-6846-4a3a-89fb-9351fcfff587/index.html#r92c1) | [T1PKS](https://docs.antismash.secondarymetabolites.org/glossary/#t1pks) | 45201 | 134641 | Low | [tricholignan A](https://mibig.secondarymetabolites.org/go/BGC0001854.4) |
| [Region 93.1](https://fungismash.secondarymetabolites.org/upload/fungi-ce3691ee-6846-4a3a-89fb-9351fcfff587/index.html#r93c1) | T1PKS,NRPS-like | 53415 | 139339 | High | [BAB/BAA](https://mibig.secondarymetabolites.org/go/BGC0002240.3) |
| [Region 101.1](https://fungismash.secondarymetabolites.org/upload/fungi-ce3691ee-6846-4a3a-89fb-9351fcfff587/index.html#r101c1) | [terpene-precursor](https://docs.antismash.secondarymetabolites.org/glossary/#terpene-precursor) | 7119 | 38417 |  |  |

Tab. S7 Analysis results of secondary metabolic biosynthesis gene clusters of *Gnomoniopsis conghuaensis*	(Continued)

| **Region** | **Type** | **From** | **To** | **Similarity Confidence** | **Most similar known cluster** |
| --- | --- | --- | --- | --- | --- |
| [Region 118.1](https://fungismash.secondarymetabolites.org/upload/fungi-ce3691ee-6846-4a3a-89fb-9351fcfff587/index.html#r118c1) | [terpene](https://docs.antismash.secondarymetabolites.org/glossary/#terpene) | 69492 | 102657 |  |  |
| [Region 119.1](https://fungismash.secondarymetabolites.org/upload/fungi-ce3691ee-6846-4a3a-89fb-9351fcfff587/index.html#r119c1) | NRPS-like,T1PKS | 66579 | 107106 |  |  |
| [Region 121.1](https://fungismash.secondarymetabolites.org/upload/fungi-ce3691ee-6846-4a3a-89fb-9351fcfff587/index.html#r121c1) | [terpene](https://docs.antismash.secondarymetabolites.org/glossary/#terpene) | 71625 | 103277 | Low | [squalestatin S1](https://mibig.secondarymetabolites.org/go/BGC0001839.3) |
| [Region 124.1](https://fungismash.secondarymetabolites.org/upload/fungi-ce3691ee-6846-4a3a-89fb-9351fcfff587/index.html#r124c1) | [isocyanide](https://docs.antismash.secondarymetabolites.org/glossary/#isocyanide) | 17728 | 80078 |  |  |
| [Region 130.1](https://fungismash.secondarymetabolites.org/upload/fungi-ce3691ee-6846-4a3a-89fb-9351fcfff587/index.html#r130c1) | [NRPS](https://docs.antismash.secondarymetabolites.org/glossary/#nrps) | 1 | 57703 |  |  |
| [Region 144.1](https://fungismash.secondarymetabolites.org/upload/fungi-ce3691ee-6846-4a3a-89fb-9351fcfff587/index.html#r144c1) | [T1PKS](https://docs.antismash.secondarymetabolites.org/glossary/#t1pks) | 1 | 53475 | High | [alternapyrone](https://mibig.secondarymetabolites.org/go/BGC0000012.3) |
| [Region 150.1](https://fungismash.secondarymetabolites.org/upload/fungi-ce3691ee-6846-4a3a-89fb-9351fcfff587/index.html#r150c1) | [terpene](https://docs.antismash.secondarymetabolites.org/glossary/#terpene) | 1 | 20260 |  |  |
| [Region 151.1](https://fungismash.secondarymetabolites.org/upload/fungi-ce3691ee-6846-4a3a-89fb-9351fcfff587/index.html#r151c1) | [T1PKS](https://docs.antismash.secondarymetabolites.org/glossary/#t1pks) | 11127 | 80319 |  |  |
| [Region 157.1](https://fungismash.secondarymetabolites.org/upload/fungi-ce3691ee-6846-4a3a-89fb-9351fcfff587/index.html#r157c1) | [terpene](https://docs.antismash.secondarymetabolites.org/glossary/#terpene) | 30817 | 61401 |  |  |
| [Region 160.1](https://fungismash.secondarymetabolites.org/upload/fungi-ce3691ee-6846-4a3a-89fb-9351fcfff587/index.html#r160c1) | NRPS-like,T1PKS | 1 | 70608 |  |  |
| [Region 161.1](https://fungismash.secondarymetabolites.org/upload/fungi-ce3691ee-6846-4a3a-89fb-9351fcfff587/index.html#r161c1) | [terpene](https://docs.antismash.secondarymetabolites.org/glossary/#terpene) | 24482 | 56203 |  |  |
| [Region 168.1](https://fungismash.secondarymetabolites.org/upload/fungi-ce3691ee-6846-4a3a-89fb-9351fcfff587/index.html#r168c1) | T1PKS,NRPS-like | 1 | 51326 |  |  |
| [Region 171.1](https://fungismash.secondarymetabolites.org/upload/fungi-ce3691ee-6846-4a3a-89fb-9351fcfff587/index.html#r171c1) | [T1PKS](https://docs.antismash.secondarymetabolites.org/glossary/#t1pks) | 20802 | 62872 |  |  |
| [Region 190.1](https://fungismash.secondarymetabolites.org/upload/fungi-ce3691ee-6846-4a3a-89fb-9351fcfff587/index.html#r190c1) | [indole](https://docs.antismash.secondarymetabolites.org/glossary/#indole) | 1 | 21343 |  |  |
| [Region 191.1](https://fungismash.secondarymetabolites.org/upload/fungi-ce3691ee-6846-4a3a-89fb-9351fcfff587/index.html#r191c1) | [T1PKS](https://docs.antismash.secondarymetabolites.org/glossary/#t1pks) | 1 | 37780 | Low | [clavatol](https://mibig.secondarymetabolites.org/go/BGC0002196.2) |
| [Region 196.1](https://fungismash.secondarymetabolites.org/upload/fungi-ce3691ee-6846-4a3a-89fb-9351fcfff587/index.html#r196c1) | [indole](https://docs.antismash.secondarymetabolites.org/glossary/#indole) | 1 | 23699 |  |  |
| [Region 209.1](https://fungismash.secondarymetabolites.org/upload/fungi-ce3691ee-6846-4a3a-89fb-9351fcfff587/index.html#r209c1) | [T1PKS](https://docs.antismash.secondarymetabolites.org/glossary/#t1pks) | 1 | 26751 |  |  |
| [Region 214.1](https://fungismash.secondarymetabolites.org/upload/fungi-ce3691ee-6846-4a3a-89fb-9351fcfff587/index.html#r214c1) | [indole](https://docs.antismash.secondarymetabolites.org/glossary/#indole) | 2409 | 22824 |  |  |
| [Region 216.1](https://fungismash.secondarymetabolites.org/upload/fungi-ce3691ee-6846-4a3a-89fb-9351fcfff587/index.html#r216c1) | [T1PKS](https://docs.antismash.secondarymetabolites.org/glossary/#t1pks) | 1 | 21471 |  |  |
| [Region 237.1](https://fungismash.secondarymetabolites.org/upload/fungi-ce3691ee-6846-4a3a-89fb-9351fcfff587/index.html#r237c1) | [NRPS](https://docs.antismash.secondarymetabolites.org/glossary/#nrps) | 1 | 14302 |  |  |
| [Region 241.1](https://fungismash.secondarymetabolites.org/upload/fungi-ce3691ee-6846-4a3a-89fb-9351fcfff587/index.html#r241c1) | [T1PKS](https://docs.antismash.secondarymetabolites.org/glossary/#t1pks) | 1 | 13041 |  |  |
| [Region 252.1](https://fungismash.secondarymetabolites.org/upload/fungi-ce3691ee-6846-4a3a-89fb-9351fcfff587/index.html#r252c1) | [terpene](https://docs.antismash.secondarymetabolites.org/glossary/#terpene) | 1 | 11470 |  |  |

Tab. S8 Analysis results of secondary metabolic biosynthesis gene clusters of *Gnomoniopsis loropetali*

| **Region** | **Type** | **From** | **To** | **Similarity Confidence** | **Most similar known cluster** |
| --- | --- | --- | --- | --- | --- |
| [Region 1.1](https://fungismash.secondarymetabolites.org/upload/fungi-3b570f3f-03a8-421b-8111-89db1af995c0/index.html#r1c1) | [terpene-precursor](https://docs.antismash.secondarymetabolites.org/glossary/#terpene-precursor) | 257089 | 288271 |  |  |
| [Region 1.2](https://fungismash.secondarymetabolites.org/upload/fungi-3b570f3f-03a8-421b-8111-89db1af995c0/index.html#r1c2) | [NRPS-like](https://docs.antismash.secondarymetabolites.org/glossary/#nrps-like) | 1208610 | 1273797 |  |  |
| [Region 1.3](https://fungismash.secondarymetabolites.org/upload/fungi-3b570f3f-03a8-421b-8111-89db1af995c0/index.html#r1c3) | [terpene](https://docs.antismash.secondarymetabolites.org/glossary/#terpene) | 1277516 | 1311640 | High | [clavaric acid](https://mibig.secondarymetabolites.org/go/BGC0001248.3) |
| [Region 2.1](https://fungismash.secondarymetabolites.org/upload/fungi-3b570f3f-03a8-421b-8111-89db1af995c0/index.html#r2c1) | [T1PKS](https://docs.antismash.secondarymetabolites.org/glossary/#t1pks) | 82515 | 150765 |  |  |
| [Region 3.1](https://fungismash.secondarymetabolites.org/upload/fungi-3b570f3f-03a8-421b-8111-89db1af995c0/index.html#r3c1) | [NRPS-like](https://docs.antismash.secondarymetabolites.org/glossary/#nrps-like) | 260228 | 324148 | High | [choline](https://mibig.secondarymetabolites.org/go/BGC0002276.2) |
| [Region 5.1](https://fungismash.secondarymetabolites.org/upload/fungi-3b570f3f-03a8-421b-8111-89db1af995c0/index.html#r5c1) | NRPS,T1PKS | 472100 | 621225 | Low | [leucinostatin A/leucinostatin B](https://mibig.secondarymetabolites.org/go/BGC0001358.4) |
| [Region 5.2](https://fungismash.secondarymetabolites.org/upload/fungi-3b570f3f-03a8-421b-8111-89db1af995c0/index.html#r5c2) | [NRPS](https://docs.antismash.secondarymetabolites.org/glossary/#nrps) | 747852 | 822096 |  |  |
| [Region 6.1](https://fungismash.secondarymetabolites.org/upload/fungi-3b570f3f-03a8-421b-8111-89db1af995c0/index.html#r6c1) | [NRPS-like](https://docs.antismash.secondarymetabolites.org/glossary/#nrps-like) | 298023 | 361755 |  |  |
| [Region 6.2](https://fungismash.secondarymetabolites.org/upload/fungi-3b570f3f-03a8-421b-8111-89db1af995c0/index.html#r6c2) | [T1PKS](https://docs.antismash.secondarymetabolites.org/glossary/#t1pks) | 621757 | 694228 |  |  |
| [Region 7.1](https://fungismash.secondarymetabolites.org/upload/fungi-3b570f3f-03a8-421b-8111-89db1af995c0/index.html#r7c1) | [T3PKS](https://docs.antismash.secondarymetabolites.org/glossary/#t3pks) | 488552 | 550093 |  |  |
| [Region 9.1](https://fungismash.secondarymetabolites.org/upload/fungi-3b570f3f-03a8-421b-8111-89db1af995c0/index.html#r9c1) | [T1PKS](https://docs.antismash.secondarymetabolites.org/glossary/#t1pks) | 763320 | 828969 | Low | [Pestheic Acid/RES-1214-2](https://mibig.secondarymetabolites.org/go/BGC0000121.3) |
| [Region 14.1](https://fungismash.secondarymetabolites.org/upload/fungi-3b570f3f-03a8-421b-8111-89db1af995c0/index.html#r14c1) | [fungal-RiPP](https://docs.antismash.secondarymetabolites.org/glossary/#fungal-ripp) | 55702 | 116893 |  |  |
| [Region 14.2](https://fungismash.secondarymetabolites.org/upload/fungi-3b570f3f-03a8-421b-8111-89db1af995c0/index.html#r14c2) | [T1PKS](https://docs.antismash.secondarymetabolites.org/glossary/#t1pks) | 140240 | 207823 |  |  |
| [Region 14.3](https://fungismash.secondarymetabolites.org/upload/fungi-3b570f3f-03a8-421b-8111-89db1af995c0/index.html#r14c3) | [T1PKS](https://docs.antismash.secondarymetabolites.org/glossary/#t1pks) | 460254 | 528326 |  |  |
| [Region 14.4](https://fungismash.secondarymetabolites.org/upload/fungi-3b570f3f-03a8-421b-8111-89db1af995c0/index.html#r14c4) | T1PKS,NRPS-like | 651578 | 724470 |  |  |
| [Region 14.5](https://fungismash.secondarymetabolites.org/upload/fungi-3b570f3f-03a8-421b-8111-89db1af995c0/index.html#r14c5) | [terpene](https://docs.antismash.secondarymetabolites.org/glossary/#terpene) | 729900 | 754464 |  |  |
| [Region 16.1](https://fungismash.secondarymetabolites.org/upload/fungi-3b570f3f-03a8-421b-8111-89db1af995c0/index.html#r16c1) | [T1PKS](https://docs.antismash.secondarymetabolites.org/glossary/#t1pks) | 19618 | 88295 | Low | [trichoxide](https://mibig.secondarymetabolites.org/go/BGC0002233.3) |
| [Region 16.2](https://fungismash.secondarymetabolites.org/upload/fungi-3b570f3f-03a8-421b-8111-89db1af995c0/index.html#r16c2) | [NRPS-like](https://docs.antismash.secondarymetabolites.org/glossary/#nrps-like) | 207009 | 270541 |  |  |
| [Region 17.1](https://fungismash.secondarymetabolites.org/upload/fungi-3b570f3f-03a8-421b-8111-89db1af995c0/index.html#r17c1) | [T1PKS](https://docs.antismash.secondarymetabolites.org/glossary/#t1pks) | 1 | 55709 | Medium | [scytalone/T3HN](https://mibig.secondarymetabolites.org/go/BGC0002161.2) |
| [Region 17.2](https://fungismash.secondarymetabolites.org/upload/fungi-3b570f3f-03a8-421b-8111-89db1af995c0/index.html#r17c2) | T1PKS,NRPS | 237263 | 354284 |  |  |
| [Region 18.1](https://fungismash.secondarymetabolites.org/upload/fungi-3b570f3f-03a8-421b-8111-89db1af995c0/index.html#r18c1) | [T1PKS](https://docs.antismash.secondarymetabolites.org/glossary/#t1pks) | 43714 | 115764 |  |  |
| [Region 21.1](https://fungismash.secondarymetabolites.org/upload/fungi-3b570f3f-03a8-421b-8111-89db1af995c0/index.html#r21c1) | [terpene](https://docs.antismash.secondarymetabolites.org/glossary/#terpene) | 11559 | 42783 |  |  |
| [Region 21.2](https://fungismash.secondarymetabolites.org/upload/fungi-3b570f3f-03a8-421b-8111-89db1af995c0/index.html#r21c2) | [indole](https://docs.antismash.secondarymetabolites.org/glossary/#indole) | 95352 | 126919 |  |  |
| [Region 23.1](https://fungismash.secondarymetabolites.org/upload/fungi-3b570f3f-03a8-421b-8111-89db1af995c0/index.html#r23c1) | [T1PKS](https://docs.antismash.secondarymetabolites.org/glossary/#t1pks) | 197465 | 265979 |  |  |
| [Region 24.1](https://fungismash.secondarymetabolites.org/upload/fungi-3b570f3f-03a8-421b-8111-89db1af995c0/index.html#r24c1) | NRPS-like,T1PKS | 72835 | 182745 | Low | [hypothemycin](https://mibig.secondarymetabolites.org/go/BGC0000077.3) |
| [Region 24.2](https://fungismash.secondarymetabolites.org/upload/fungi-3b570f3f-03a8-421b-8111-89db1af995c0/index.html#r24c2) | [NRPS-like](https://docs.antismash.secondarymetabolites.org/glossary/#nrps-like) | 289585 | 353031 |  |  |
| [Region 25.1](https://fungismash.secondarymetabolites.org/upload/fungi-3b570f3f-03a8-421b-8111-89db1af995c0/index.html#r25c1) | [T1PKS](https://docs.antismash.secondarymetabolites.org/glossary/#t1pks) | 36948 | 104945 | Low | [flavoglaucin](https://mibig.secondarymetabolites.org/go/BGC0002234.3) |
| [Region 25.2](https://fungismash.secondarymetabolites.org/upload/fungi-3b570f3f-03a8-421b-8111-89db1af995c0/index.html#r25c2) | [terpene](https://docs.antismash.secondarymetabolites.org/glossary/#terpene) | 286637 | 318827 |  |  |
| [Region 25.3](https://fungismash.secondarymetabolites.org/upload/fungi-3b570f3f-03a8-421b-8111-89db1af995c0/index.html#r25c3) | [T1PKS](https://docs.antismash.secondarymetabolites.org/glossary/#t1pks) | 484303 | 528835 |  |  |
| [Region 26.1](https://fungismash.secondarymetabolites.org/upload/fungi-3b570f3f-03a8-421b-8111-89db1af995c0/index.html#r26c1) | [T1PKS](https://docs.antismash.secondarymetabolites.org/glossary/#t1pks) | 204534 | 272428 |  |  |
| [Region 26.2](https://fungismash.secondarymetabolites.org/upload/fungi-3b570f3f-03a8-421b-8111-89db1af995c0/index.html#r26c2) | [NRPS](https://docs.antismash.secondarymetabolites.org/glossary/#nrps) | 425540 | 489430 |  |  |
| [Region 27.1](https://fungismash.secondarymetabolites.org/upload/fungi-3b570f3f-03a8-421b-8111-89db1af995c0/index.html#r27c1) | [terpene](https://docs.antismash.secondarymetabolites.org/glossary/#terpene) | 67417 | 99521 |  |  |
| [Region 28.1](https://fungismash.secondarymetabolites.org/upload/fungi-3b570f3f-03a8-421b-8111-89db1af995c0/index.html#r28c1) | [T1PKS](https://docs.antismash.secondarymetabolites.org/glossary/#t1pks) | 257005 | 324988 | High | [alternapyrone](https://mibig.secondarymetabolites.org/go/BGC0000012.3) |
| [Region 29.1](https://fungismash.secondarymetabolites.org/upload/fungi-3b570f3f-03a8-421b-8111-89db1af995c0/index.html#r29c1) | [terpene](https://docs.antismash.secondarymetabolites.org/glossary/#terpene) | 288196 | 320749 |  |  |
| [Region 32.1](https://fungismash.secondarymetabolites.org/upload/fungi-3b570f3f-03a8-421b-8111-89db1af995c0/index.html#r32c1) | NRPS-like,NRPS | 100777 | 186208 |  |  |
| [Region 33.1](https://fungismash.secondarymetabolites.org/upload/fungi-3b570f3f-03a8-421b-8111-89db1af995c0/index.html#r33c1) | [T1PKS](https://docs.antismash.secondarymetabolites.org/glossary/#t1pks) | 143024 | 211724 |  |  |
| [Region 34.1](https://fungismash.secondarymetabolites.org/upload/fungi-3b570f3f-03a8-421b-8111-89db1af995c0/index.html#r34c1) | [terpene](https://docs.antismash.secondarymetabolites.org/glossary/#terpene) | 11938 | 43618 |  |  |
| [Region 34.2](https://fungismash.secondarymetabolites.org/upload/fungi-3b570f3f-03a8-421b-8111-89db1af995c0/index.html#r34c2) | [other](https://docs.antismash.secondarymetabolites.org/glossary/#other) | 222713 | 283627 |  |  |
| [Region 35.1](https://fungismash.secondarymetabolites.org/upload/fungi-3b570f3f-03a8-421b-8111-89db1af995c0/index.html#r35c1) | [terpene-precursor](https://docs.antismash.secondarymetabolites.org/glossary/#terpene-precursor) | 227673 | 258950 |  |  |
| [Region 35.2](https://fungismash.secondarymetabolites.org/upload/fungi-3b570f3f-03a8-421b-8111-89db1af995c0/index.html#r35c2) | [terpene](https://docs.antismash.secondarymetabolites.org/glossary/#terpene) | 343529 | 374599 | Medium | [HEx-tc1 terpene](https://mibig.secondarymetabolites.org/go/BGC0002216.3) |

Tab. S8 Analysis results of secondary metabolic biosynthesis gene clusters of *Gnomoniopsis loropetali*	(Continued)

| **Region** | **Type** | **From** | **To** | **Similarity Confidence** | **Most similar known cluster** |
| --- | --- | --- | --- | --- | --- |
| [Region 37.1](https://fungismash.secondarymetabolites.org/upload/fungi-3b570f3f-03a8-421b-8111-89db1af995c0/index.html#r37c1) | NRP-metallophore,NRPS | 61877 | 140529 |  |  |
| [Region 37.2](https://fungismash.secondarymetabolites.org/upload/fungi-3b570f3f-03a8-421b-8111-89db1af995c0/index.html#r37c2) | [T1PKS](https://docs.antismash.secondarymetabolites.org/glossary/#t1pks) | 301492 | 370421 |  |  |
| [Region 38.1](https://fungismash.secondarymetabolites.org/upload/fungi-3b570f3f-03a8-421b-8111-89db1af995c0/index.html#r38c1) | NRPS-like,T1PKS | 1 | 49363 |  |  |
| [Region 41.1](https://fungismash.secondarymetabolites.org/upload/fungi-3b570f3f-03a8-421b-8111-89db1af995c0/index.html#r41c1) | [T1PKS](https://docs.antismash.secondarymetabolites.org/glossary/#t1pks) | 116436 | 184927 | High | [alternariol](https://mibig.secondarymetabolites.org/go/BGC0001284.4) |
| [Region 42.1](https://fungismash.secondarymetabolites.org/upload/fungi-3b570f3f-03a8-421b-8111-89db1af995c0/index.html#r42c1) | [terpene](https://docs.antismash.secondarymetabolites.org/glossary/#terpene) | 50858 | 82509 |  |  |
| [Region 42.2](https://fungismash.secondarymetabolites.org/upload/fungi-3b570f3f-03a8-421b-8111-89db1af995c0/index.html#r42c2) | NRPS,T1PKS | 118640 | 191303 |  |  |
| [Region 43.1](https://fungismash.secondarymetabolites.org/upload/fungi-3b570f3f-03a8-421b-8111-89db1af995c0/index.html#r43c1) | [isocyanide](https://docs.antismash.secondarymetabolites.org/glossary/#isocyanide) | 26262 | 88475 |  |  |
| [Region 51.1](https://fungismash.secondarymetabolites.org/upload/fungi-3b570f3f-03a8-421b-8111-89db1af995c0/index.html#r51c1) | [T1PKS](https://docs.antismash.secondarymetabolites.org/glossary/#t1pks) | 87802 | 155791 |  |  |
| [Region 57.1](https://fungismash.secondarymetabolites.org/upload/fungi-3b570f3f-03a8-421b-8111-89db1af995c0/index.html#r57c1) | [NRPS-like](https://docs.antismash.secondarymetabolites.org/glossary/#nrps-like) | 38131 | 102400 |  |  |
| [Region 59.1](https://fungismash.secondarymetabolites.org/upload/fungi-3b570f3f-03a8-421b-8111-89db1af995c0/index.html#r59c1) | [NI-siderophore](https://docs.antismash.secondarymetabolites.org/glossary/#ni-siderophore) | 203733 | 239760 |  |  |
| [Region 60.1](https://fungismash.secondarymetabolites.org/upload/fungi-3b570f3f-03a8-421b-8111-89db1af995c0/index.html#r60c1) | [terpene](https://docs.antismash.secondarymetabolites.org/glossary/#terpene) | 193736 | 226708 |  |  |
| [Region 63.1](https://fungismash.secondarymetabolites.org/upload/fungi-3b570f3f-03a8-421b-8111-89db1af995c0/index.html#r63c1) | [terpene](https://docs.antismash.secondarymetabolites.org/glossary/#terpene) | 94498 | 128562 |  |  |
| [Region 70.1](https://fungismash.secondarymetabolites.org/upload/fungi-3b570f3f-03a8-421b-8111-89db1af995c0/index.html#r70c1) | [terpene](https://docs.antismash.secondarymetabolites.org/glossary/#terpene) | 34746 | 66414 | Low | [squalestatin S1](https://mibig.secondarymetabolites.org/go/BGC0001839.3) |
| [Region 82.1](https://fungismash.secondarymetabolites.org/upload/fungi-3b570f3f-03a8-421b-8111-89db1af995c0/index.html#r82c1) | [NRPS-like](https://docs.antismash.secondarymetabolites.org/glossary/#nrps-like) | 1 | 41709 |  |  |
| [Region 82.2](https://fungismash.secondarymetabolites.org/upload/fungi-3b570f3f-03a8-421b-8111-89db1af995c0/index.html#r82c2) | [terpene](https://docs.antismash.secondarymetabolites.org/glossary/#terpene) | 69312 | 101910 |  |  |
| [Region 95.1](https://fungismash.secondarymetabolites.org/upload/fungi-3b570f3f-03a8-421b-8111-89db1af995c0/index.html#r95c1) | [NRPS-like](https://docs.antismash.secondarymetabolites.org/glossary/#nrps-like) | 1 | 59888 | Low | [cytoglobosin X](https://mibig.secondarymetabolites.org/go/BGC0002546.3) |
| [Region 99.1](https://fungismash.secondarymetabolites.org/upload/fungi-3b570f3f-03a8-421b-8111-89db1af995c0/index.html#r99c1) | T1PKS,terpene | 1 | 66735 | Low | [depudecin](https://mibig.secondarymetabolites.org/go/BGC0000046.4) |
| [Region 120.1](https://fungismash.secondarymetabolites.org/upload/fungi-3b570f3f-03a8-421b-8111-89db1af995c0/index.html#r120c1) | [indole](https://docs.antismash.secondarymetabolites.org/glossary/#indole) | 1 | 13985 |  |  |

Tab. S9 Analysis results of secondary metabolic biosynthesis gene clusters of *Gnomoniopsis qixiensis*

| **Region** | **Type** | **From** | **To** | **Similarity Confidence** | **Most similar known cluster** |
| --- | --- | --- | --- | --- | --- |
| [Region 1.1](https://fungismash.secondarymetabolites.org/upload/fungi-9aafede0-8f80-4b4d-8f47-bbbefff8e76d/index.html#r1c1) | [NRPS-like](https://docs.antismash.secondarymetabolites.org/glossary/#nrps-like) | 1240653 | 1304229 |  |  |
| [Region 2.1](https://fungismash.secondarymetabolites.org/upload/fungi-9aafede0-8f80-4b4d-8f47-bbbefff8e76d/index.html#r2c1) | [T1PKS](https://docs.antismash.secondarymetabolites.org/glossary/#t1pks) | 119035 | 187458 |  |  |
| [Region 2.2](https://fungismash.secondarymetabolites.org/upload/fungi-9aafede0-8f80-4b4d-8f47-bbbefff8e76d/index.html#r2c2) | [T1PKS](https://docs.antismash.secondarymetabolites.org/glossary/#t1pks) | 579723 | 647689 | High | [alternapyrone](https://mibig.secondarymetabolites.org/go/BGC0000012.3) |
| [Region 2.3](https://fungismash.secondarymetabolites.org/upload/fungi-9aafede0-8f80-4b4d-8f47-bbbefff8e76d/index.html#r2c3) | [NRPS-like](https://docs.antismash.secondarymetabolites.org/glossary/#nrps-like) | 648543 | 710960 |  |  |
| [Region 2.4](https://fungismash.secondarymetabolites.org/upload/fungi-9aafede0-8f80-4b4d-8f47-bbbefff8e76d/index.html#r2c4) | [NRPS-like](https://docs.antismash.secondarymetabolites.org/glossary/#nrps-like) | 1374819 | 1438276 |  |  |
| [Region 3.1](https://fungismash.secondarymetabolites.org/upload/fungi-9aafede0-8f80-4b4d-8f47-bbbefff8e76d/index.html#r3c1) | [T1PKS](https://docs.antismash.secondarymetabolites.org/glossary/#t1pks) | 734430 | 800545 | Medium | [3'-methoxy-1,2-dehydropenicillide/pestalotiollide B/pestalotiollide C/1,2-dehydropenicillide/3-methoxy-1,2-dehydropenicillide/1,2-epoxy-3,4-didehydropenicillide](https://mibig.secondarymetabolites.org/go/BGC0002244.3) |
| [Region 4.1](https://fungismash.secondarymetabolites.org/upload/fungi-9aafede0-8f80-4b4d-8f47-bbbefff8e76d/index.html#r4c1) | [NI-siderophore](https://docs.antismash.secondarymetabolites.org/glossary/#ni-siderophore) | 304660 | 348850 |  |  |
| [Region 4.2](https://fungismash.secondarymetabolites.org/upload/fungi-9aafede0-8f80-4b4d-8f47-bbbefff8e76d/index.html#r4c2) | [terpene](https://docs.antismash.secondarymetabolites.org/glossary/#terpene) | 872607 | 904155 |  |  |
| [Region 4.3](https://fungismash.secondarymetabolites.org/upload/fungi-9aafede0-8f80-4b4d-8f47-bbbefff8e76d/index.html#r4c3) | [NRPS-like](https://docs.antismash.secondarymetabolites.org/glossary/#nrps-like) | 1266533 | 1325989 |  |  |
| [Region 5.1](https://fungismash.secondarymetabolites.org/upload/fungi-9aafede0-8f80-4b4d-8f47-bbbefff8e76d/index.html#r5c1) | [T1PKS](https://docs.antismash.secondarymetabolites.org/glossary/#t1pks) | 11537 | 81708 |  |  |
| [Region 5.2](https://fungismash.secondarymetabolites.org/upload/fungi-9aafede0-8f80-4b4d-8f47-bbbefff8e76d/index.html#r5c2) | [terpene](https://docs.antismash.secondarymetabolites.org/glossary/#terpene) | 295405 | 325989 |  |  |
| [Region 6.1](https://fungismash.secondarymetabolites.org/upload/fungi-9aafede0-8f80-4b4d-8f47-bbbefff8e76d/index.html#r6c1) | [terpene-precursor](https://docs.antismash.secondarymetabolites.org/glossary/#terpene-precursor) | 521933 | 553207 |  |  |
| [Region 7.1](https://fungismash.secondarymetabolites.org/upload/fungi-9aafede0-8f80-4b4d-8f47-bbbefff8e76d/index.html#r7c1) | isocyanide,T1PKS,indole,terpene | 137784 | 237475 |  |  |
| [Region 7.2](https://fungismash.secondarymetabolites.org/upload/fungi-9aafede0-8f80-4b4d-8f47-bbbefff8e76d/index.html#r7c2) | [T1PKS](https://docs.antismash.secondarymetabolites.org/glossary/#t1pks) | 836375 | 904535 |  |  |
| [Region 8.1](https://fungismash.secondarymetabolites.org/upload/fungi-9aafede0-8f80-4b4d-8f47-bbbefff8e76d/index.html#r8c1) | [T1PKS](https://docs.antismash.secondarymetabolites.org/glossary/#t1pks) | 86935 | 158196 | Low | [monacolin K](https://mibig.secondarymetabolites.org/go/BGC0000098.3) |
| [Region 8.2](https://fungismash.secondarymetabolites.org/upload/fungi-9aafede0-8f80-4b4d-8f47-bbbefff8e76d/index.html#r8c2) | [T1PKS](https://docs.antismash.secondarymetabolites.org/glossary/#t1pks) | 455254 | 523750 |  |  |
| [Region 8.3](https://fungismash.secondarymetabolites.org/upload/fungi-9aafede0-8f80-4b4d-8f47-bbbefff8e76d/index.html#r8c3) | NRPS-like,terpene | 795007 | 858513 |  |  |
| [Region 8.4](https://fungismash.secondarymetabolites.org/upload/fungi-9aafede0-8f80-4b4d-8f47-bbbefff8e76d/index.html#r8c4) | [T1PKS](https://docs.antismash.secondarymetabolites.org/glossary/#t1pks) | 910198 | 967191 | High | [betaenone C/probetaenone I/stemphyloxin II/compound 4/compound 6/compound 7/compound 9/compound 3/dehydroprobetaenone I/compound 5](https://mibig.secondarymetabolites.org/go/BGC0002165.3) |
| [Region 9.1](https://fungismash.secondarymetabolites.org/upload/fungi-9aafede0-8f80-4b4d-8f47-bbbefff8e76d/index.html#r9c1) | [terpene](https://docs.antismash.secondarymetabolites.org/glossary/#terpene) | 249355 | 281295 |  |  |
| [Region 9.2](https://fungismash.secondarymetabolites.org/upload/fungi-9aafede0-8f80-4b4d-8f47-bbbefff8e76d/index.html#r9c2) | NRPS,T1PKS | 397881 | 478197 |  |  |
| [Region 9.3](https://fungismash.secondarymetabolites.org/upload/fungi-9aafede0-8f80-4b4d-8f47-bbbefff8e76d/index.html#r9c3) | [T1PKS](https://docs.antismash.secondarymetabolites.org/glossary/#t1pks) | 511802 | 578673 |  |  |
| [Region 11.1](https://fungismash.secondarymetabolites.org/upload/fungi-9aafede0-8f80-4b4d-8f47-bbbefff8e76d/index.html#r11c1) | [other](https://docs.antismash.secondarymetabolites.org/glossary/#other) | 165140 | 226054 |  |  |
| [Region 11.2](https://fungismash.secondarymetabolites.org/upload/fungi-9aafede0-8f80-4b4d-8f47-bbbefff8e76d/index.html#r11c2) | [terpene](https://docs.antismash.secondarymetabolites.org/glossary/#terpene) | 347378 | 381267 |  |  |
| [Region 13.1](https://fungismash.secondarymetabolites.org/upload/fungi-9aafede0-8f80-4b4d-8f47-bbbefff8e76d/index.html#r13c1) | [terpene](https://docs.antismash.secondarymetabolites.org/glossary/#terpene) | 307680 | 338839 |  |  |
| [Region 13.2](https://fungismash.secondarymetabolites.org/upload/fungi-9aafede0-8f80-4b4d-8f47-bbbefff8e76d/index.html#r13c2) | [indole](https://docs.antismash.secondarymetabolites.org/glossary/#indole) | 385371 | 416729 |  |  |
| [Region 15.1](https://fungismash.secondarymetabolites.org/upload/fungi-9aafede0-8f80-4b4d-8f47-bbbefff8e76d/index.html#r15c1) | NRPS,T1PKS | 210335 | 283611 | Low | [cytoglobosin X](https://mibig.secondarymetabolites.org/go/BGC0002546.3) |
| [Region 16.1](https://fungismash.secondarymetabolites.org/upload/fungi-9aafede0-8f80-4b4d-8f47-bbbefff8e76d/index.html#r16c1) | T1PKS,NRPS | 409846 | 520422 | Low | [trichoxide](https://mibig.secondarymetabolites.org/go/BGC0002233.3) |
| [Region 16.2](https://fungismash.secondarymetabolites.org/upload/fungi-9aafede0-8f80-4b4d-8f47-bbbefff8e76d/index.html#r16c2) | [terpene](https://docs.antismash.secondarymetabolites.org/glossary/#terpene) | 658033 | 689609 |  |  |

Tab. S9 Analysis results of secondary metabolic biosynthesis gene clusters of *Gnomoniopsis qixiensis*	(Continued)

| **Region** | **Type** | **From** | **To** | **Similarity Confidence** | **Most similar known cluster** |
| --- | --- | --- | --- | --- | --- |
| [Region 17.1](https://fungismash.secondarymetabolites.org/upload/fungi-9aafede0-8f80-4b4d-8f47-bbbefff8e76d/index.html#r17c1) | T1PKS,NRPS-like | 467963 | 559833 |  |  |
| [Region 17.2](https://fungismash.secondarymetabolites.org/upload/fungi-9aafede0-8f80-4b4d-8f47-bbbefff8e76d/index.html#r17c2) | NRPS-like,NRP-metallophore,NRPS | 640435 | 759857 |  |  |
| [Region 18.1](https://fungismash.secondarymetabolites.org/upload/fungi-9aafede0-8f80-4b4d-8f47-bbbefff8e76d/index.html#r18c1) | [terpene](https://docs.antismash.secondarymetabolites.org/glossary/#terpene) | 262847 | 294313 |  |  |
| [Region 18.2](https://fungismash.secondarymetabolites.org/upload/fungi-9aafede0-8f80-4b4d-8f47-bbbefff8e76d/index.html#r18c2) | [phosphonate](https://docs.antismash.secondarymetabolites.org/glossary/#phosphonate) | 384149 | 400489 |  |  |
| [Region 18.3](https://fungismash.secondarymetabolites.org/upload/fungi-9aafede0-8f80-4b4d-8f47-bbbefff8e76d/index.html#r18c3) | T1PKS,T3PKS | 448356 | 518898 |  |  |
| [Region 18.4](https://fungismash.secondarymetabolites.org/upload/fungi-9aafede0-8f80-4b4d-8f47-bbbefff8e76d/index.html#r18c4) | NRPS-like,T1PKS | 583740 | 680891 | Low | [hypothemycin](https://mibig.secondarymetabolites.org/go/BGC0000077.3) |
| [Region 18.5](https://fungismash.secondarymetabolites.org/upload/fungi-9aafede0-8f80-4b4d-8f47-bbbefff8e76d/index.html#r18c5) | [NRPS-like](https://docs.antismash.secondarymetabolites.org/glossary/#nrps-like) | 698500 | 754746 |  |  |
| [Region 19.1](https://fungismash.secondarymetabolites.org/upload/fungi-9aafede0-8f80-4b4d-8f47-bbbefff8e76d/index.html#r19c1) | [NRPS-like](https://docs.antismash.secondarymetabolites.org/glossary/#nrps-like) | 658600 | 692027 | High | [choline](https://mibig.secondarymetabolites.org/go/BGC0002276.2) |
| [Region 20.1](https://fungismash.secondarymetabolites.org/upload/fungi-9aafede0-8f80-4b4d-8f47-bbbefff8e76d/index.html#r20c1) | [terpene](https://docs.antismash.secondarymetabolites.org/glossary/#terpene) | 439881 | 471523 | Low | [squalestatin S1](https://mibig.secondarymetabolites.org/go/BGC0001839.3) |
| [Region 21.1](https://fungismash.secondarymetabolites.org/upload/fungi-9aafede0-8f80-4b4d-8f47-bbbefff8e76d/index.html#r21c1) | [NRPS-like](https://docs.antismash.secondarymetabolites.org/glossary/#nrps-like) | 418245 | 481664 |  |  |
| [Region 22.1](https://fungismash.secondarymetabolites.org/upload/fungi-9aafede0-8f80-4b4d-8f47-bbbefff8e76d/index.html#r22c1) | [T1PKS](https://docs.antismash.secondarymetabolites.org/glossary/#t1pks) | 336047 | 404077 |  |  |
| [Region 25.1](https://fungismash.secondarymetabolites.org/upload/fungi-9aafede0-8f80-4b4d-8f47-bbbefff8e76d/index.html#r25c1) | [T1PKS](https://docs.antismash.secondarymetabolites.org/glossary/#t1pks) | 180457 | 248341 |  |  |
| [Region 31.1](https://fungismash.secondarymetabolites.org/upload/fungi-9aafede0-8f80-4b4d-8f47-bbbefff8e76d/index.html#r31c1) | [NRPS-like](https://docs.antismash.secondarymetabolites.org/glossary/#nrps-like) | 166518 | 229995 |  |  |
| [Region 34.1](https://fungismash.secondarymetabolites.org/upload/fungi-9aafede0-8f80-4b4d-8f47-bbbefff8e76d/index.html#r34c1) | [T1PKS](https://docs.antismash.secondarymetabolites.org/glossary/#t1pks) | 37799 | 104498 | Medium | [scytalone/T3HN](https://mibig.secondarymetabolites.org/go/BGC0002161.2) |
| [Region 36.1](https://fungismash.secondarymetabolites.org/upload/fungi-9aafede0-8f80-4b4d-8f47-bbbefff8e76d/index.html#r36c1) | [NRPS-like](https://docs.antismash.secondarymetabolites.org/glossary/#nrps-like) | 121000 | 185287 |  |  |
| [Region 36.2](https://fungismash.secondarymetabolites.org/upload/fungi-9aafede0-8f80-4b4d-8f47-bbbefff8e76d/index.html#r36c2) | [T3PKS](https://docs.antismash.secondarymetabolites.org/glossary/#t3pks) | 279260 | 340782 |  |  |
| [Region 37.1](https://fungismash.secondarymetabolites.org/upload/fungi-9aafede0-8f80-4b4d-8f47-bbbefff8e76d/index.html#r37c1) | [terpene](https://docs.antismash.secondarymetabolites.org/glossary/#terpene) | 245110 | 276195 | High | [HEx-tc1 terpene](https://mibig.secondarymetabolites.org/go/BGC0002216.3) |
| [Region 41.1](https://fungismash.secondarymetabolites.org/upload/fungi-9aafede0-8f80-4b4d-8f47-bbbefff8e76d/index.html#r41c1) | [NRPS](https://docs.antismash.secondarymetabolites.org/glossary/#nrps) | 234142 | 308344 |  |  |
| [Region 43.1](https://fungismash.secondarymetabolites.org/upload/fungi-9aafede0-8f80-4b4d-8f47-bbbefff8e76d/index.html#r43c1) | [T1PKS](https://docs.antismash.secondarymetabolites.org/glossary/#t1pks) | 46128 | 113692 |  |  |
| [Region 45.1](https://fungismash.secondarymetabolites.org/upload/fungi-9aafede0-8f80-4b4d-8f47-bbbefff8e76d/index.html#r45c1) | [terpene](https://docs.antismash.secondarymetabolites.org/glossary/#terpene) | 38335 | 70083 |  |  |
| [Region 47.1](https://fungismash.secondarymetabolites.org/upload/fungi-9aafede0-8f80-4b4d-8f47-bbbefff8e76d/index.html#r47c1) | NRPS,NRPS-like | 71116 | 162293 |  |  |
| [Region 48.1](https://fungismash.secondarymetabolites.org/upload/fungi-9aafede0-8f80-4b4d-8f47-bbbefff8e76d/index.html#r48c1) | NRPS-like,T1PKS | 1 | 46459 |  |  |
| [Region 49.1](https://fungismash.secondarymetabolites.org/upload/fungi-9aafede0-8f80-4b4d-8f47-bbbefff8e76d/index.html#r49c1) | [terpene](https://docs.antismash.secondarymetabolites.org/glossary/#terpene) | 153629 | 185001 |  |  |
| [Region 52.1](https://fungismash.secondarymetabolites.org/upload/fungi-9aafede0-8f80-4b4d-8f47-bbbefff8e76d/index.html#r52c1) | [terpene-precursor](https://docs.antismash.secondarymetabolites.org/glossary/#terpene-precursor) | 46428 | 77630 |  |  |
| [Region 53.1](https://fungismash.secondarymetabolites.org/upload/fungi-9aafede0-8f80-4b4d-8f47-bbbefff8e76d/index.html#r53c1) | [terpene](https://docs.antismash.secondarymetabolites.org/glossary/#terpene) | 95853 | 127974 |  |  |
| [Region 55.1](https://fungismash.secondarymetabolites.org/upload/fungi-9aafede0-8f80-4b4d-8f47-bbbefff8e76d/index.html#r55c1) | [NRPS](https://docs.antismash.secondarymetabolites.org/glossary/#nrps) | 79818 | 143635 |  |  |
| [Region 57.1](https://fungismash.secondarymetabolites.org/upload/fungi-9aafede0-8f80-4b4d-8f47-bbbefff8e76d/index.html#r57c1) | [terpene](https://docs.antismash.secondarymetabolites.org/glossary/#terpene) | 213353 | 247789 |  |  |
| [Region 60.1](https://fungismash.secondarymetabolites.org/upload/fungi-9aafede0-8f80-4b4d-8f47-bbbefff8e76d/index.html#r60c1) | [nitropropanoic_acid](https://docs.antismash.secondarymetabolites.org/glossary/#nitropropanoic_acid) | 53488 | 75047 |  |  |
| [Region 62.1](https://fungismash.secondarymetabolites.org/upload/fungi-9aafede0-8f80-4b4d-8f47-bbbefff8e76d/index.html#r62c1) | [T1PKS](https://docs.antismash.secondarymetabolites.org/glossary/#t1pks) | 1 | 47550 |  |  |
| [Region 63.1](https://fungismash.secondarymetabolites.org/upload/fungi-9aafede0-8f80-4b4d-8f47-bbbefff8e76d/index.html#r63c1) | [T1PKS](https://docs.antismash.secondarymetabolites.org/glossary/#t1pks) | 30661 | 96437 | High | [neosartorin](https://mibig.secondarymetabolites.org/go/BGC0001988.3) |
| [Region 67.1](https://fungismash.secondarymetabolites.org/upload/fungi-9aafede0-8f80-4b4d-8f47-bbbefff8e76d/index.html#r67c1) | T1PKS,NRPS-like | 35284 | 117575 | High | [BAB/BAA](https://mibig.secondarymetabolites.org/go/BGC0002240.3) |
| [Region 71.1](https://fungismash.secondarymetabolites.org/upload/fungi-9aafede0-8f80-4b4d-8f47-bbbefff8e76d/index.html#r71c1) | terpene,T1PKS | 36057 | 114980 | Medium | [HEx-tc1 terpene](https://mibig.secondarymetabolites.org/go/BGC0002216.3) |
| [Region 73.1](https://fungismash.secondarymetabolites.org/upload/fungi-9aafede0-8f80-4b4d-8f47-bbbefff8e76d/index.html#r73c1) | T1PKS,NRPS-like | 27043 | 98878 |  |  |
| [Region 75.1](https://fungismash.secondarymetabolites.org/upload/fungi-9aafede0-8f80-4b4d-8f47-bbbefff8e76d/index.html#r75c1) | [NRPS-like](https://docs.antismash.secondarymetabolites.org/glossary/#nrps-like) | 1 | 57861 |  |  |
| [Region 75.2](https://fungismash.secondarymetabolites.org/upload/fungi-9aafede0-8f80-4b4d-8f47-bbbefff8e76d/index.html#r75c2) | [terpene](https://docs.antismash.secondarymetabolites.org/glossary/#terpene) | 80097 | 112589 | High | [clavaric acid](https://mibig.secondarymetabolites.org/go/BGC0001248.3) |
| [Region 77.1](https://fungismash.secondarymetabolites.org/upload/fungi-9aafede0-8f80-4b4d-8f47-bbbefff8e76d/index.html#r77c1) | [T1PKS](https://docs.antismash.secondarymetabolites.org/glossary/#t1pks) | 77202 | 118524 |  |  |
| [Region 78.1](https://fungismash.secondarymetabolites.org/upload/fungi-9aafede0-8f80-4b4d-8f47-bbbefff8e76d/index.html#r78c1) | [T1PKS](https://docs.antismash.secondarymetabolites.org/glossary/#t1pks) | 60297 | 112558 | Low | [depudecin](https://mibig.secondarymetabolites.org/go/BGC0000046.4) |

Tab. S9 Analysis results of secondary metabolic biosynthesis gene clusters of *Gnomoniopsis qixiensis*	(Continued)

| **Region** | **Type** | **From** | **To** | **Similarity Confidence** | **Most similar known cluster** |
| --- | --- | --- | --- | --- | --- |
| [Region 87.1](https://fungismash.secondarymetabolites.org/upload/fungi-9aafede0-8f80-4b4d-8f47-bbbefff8e76d/index.html#r87c1) | [NRPS-like](https://docs.antismash.secondarymetabolites.org/glossary/#nrps-like) | 40699 | 78713 | Low | [sorbicillin/2'3'-dihydrosorbicillin/sorbicillinol/oxosorbicillinol/bisvertinolone (spelled bisvertinolon in paper)/dihydrobisvertinolone/tetrahydrobisvertinolone/bisorbicillinol/2'3'-dihydrosorbicillinol](https://mibig.secondarymetabolites.org/go/BGC0002190.2) |
| [Region 89.1](https://fungismash.secondarymetabolites.org/upload/fungi-9aafede0-8f80-4b4d-8f47-bbbefff8e76d/index.html#r89c1) | [T1PKS](https://docs.antismash.secondarymetabolites.org/glossary/#t1pks) | 1 | 41071 |  |  |
